# Improved power and precision with whole genome sequencing data in genome-wide association studies of inflammatory biomarkers

**DOI:** 10.1038/s41598-019-53111-7

**Published:** 2019-11-14

**Authors:** Julia Höglund, Nima Rafati, Mathias Rask-Andersen, Stefan Enroth, Torgny Karlsson, Weronica E. Ek, Åsa Johansson

**Affiliations:** Department of Immunology, Genetics and Pathology, Science for Life Laboratory, Uppsala University, Uppsala, Sweden

**Keywords:** Genome-wide association studies, Genetic variation

## Abstract

Genome-wide association studies (GWAS) have identified associations between thousands of common genetic variants and human traits. However, common variants usually explain a limited fraction of the heritability of a trait. A powerful resource for identifying trait-associated variants is whole genome sequencing (WGS) data in cohorts comprised of families or individuals from a limited geographical area. To evaluate the power of WGS compared to imputations, we performed GWAS on WGS data for 72 inflammatory biomarkers, in a kinship-structured cohort. When using WGS data, we identified 18 novel associations that were not detected when analyzing the same biomarkers with genotyped or imputed SNPs. Five of the novel top variants were low frequency variants with a minor allele frequency (MAF) of <5%. Our results suggest that, even when applying a GWAS approach, we gain power and precision using WGS data, presumably due to more accurate determination of genotypes. The lack of a comparable dataset for replication of our results is a limitation in our study. However, this further highlights that there is a need for more genetic epidemiological studies based on WGS data.

## Introduction

Over the past decade, genome-wide association studies (GWAS) have successfully identified associations of thousands of single-nucleotide polymorphisms (SNPs) with human traits and diseases^1^. Most of the associated alleles discovered so far are common, with a minor allele frequency (MAF) above 5%^2^. Many SNPs are also located outside coding regions, which complicates the identification of causal mechanisms, functional variants and relevant genes. Additionally, identified SNPs collectively only explain a limited fraction of the heritability^3–5^. A number of hypotheses for this “hidden heritability” have been proposed, such that part of the heritability is due to rare variants^6^, that there is a non-negligible fraction of unmapped or untagged common variants^7^, or that variants with very low effect sizes have not been captured in current GWAS. The extent to which rare and low-frequency coding variants (<5%) influence traits and diseases is still not completely understood^2^. Rare variants may not be present on currently available SNP arrays nor be well tagged by the available SNPs^8^ on the array due to the low linkage disequilibrium (LD) between common SNPs and rare variants^9^. Performing GWAS with imputed or genotyped variants is therefore not ideal for detecting associations with rare variants. Additionally, rare variants are often specific to individual populations^7,10–12^, or even families, making them hard to detect with standard GWAS in unrelated participants.

However, the limitations in association studies can be partly reduced. Firstly, a powerful approach to identify complex trait- and disease-associated rare variants is to use populations that comprise of families or individuals from a limited geographical area^13,14^. Secondly, whole genome sequencing (WGS) data can be used to better capture rare and low frequency variants and variants not in LD with SNPs on a genotyping array. WGS is superior to imputation when it comes to determining genotypes of rare variants with high accuracy^8^. Simulation studies have shown that the mapping precision for rare variants increases considerably when using WGS data in a GWAS approach, making it an efficient way of detecting and fine-mapping rare variants simultaneously^15^. Hence, by shifting from genotyped and imputed data to WGS data, a standard GWAS can be performed with a likely increase in both variant capture and precision. Yet, few GWAS have been performed using WGS data to date. During the last years, WGS has been performed in a variety of different populations^7,11,12,16^. A recent study within a small kinship-structured cohort (similar to ours), tested for the burden of rare variants from WGS data on six cardiometabolic traits^17^. The authors found novel signals that neither were captured with low-depth sequencing, nor with genome-wide genotyping with dense imputation in the same samples^17^, supporting the notion that WGS data in kinship-structured cohorts can improve power to identify genetic associations. A number of studies have also performed GWAS in cohorts where the variants were imputed from unique reference panels based on WGS of a subset of the participants of the same cohorts, or performed GWAS on low coverage (~4×) WGS data^18–22^. For example, in a study on circulating lipid levels and five inflammatory biomarkers^22^, WGS of 2,120 Sardinians was performed to assess the impact of the variants common in the Sardinian cohort but rare in the 1000 Genomes Project. In total, 14 signals were found, including two new loci that would have been missed if data had been imputed using the 1000 Genomes reference panel, further underlining the advantages of large-scale sequencing.

Biomarkers are often strongly genetically regulated^23,24^ and have been shown to be less polygenic in comparison to complex traits and diseases, which increases the power to study the effect in a smaller cohort where WGS data is available. When used for diagnosis, an ideal biomarker should be uniquely present or overexpressed in the tissue of interest and not be influenced by confounding factors, such as genetic variants^23,24^. However, genetic factors commonly have a considerable effect on biomarker levels and introduce noise when biomarkers are used for diagnosis. Better characterization of the genetic contribution to variation in biomarker levels is therefore of great importance.

In this project, we used a GWAS approach to test for associations with no fewer than 72 inflammatory plasma protein biomarkers, in order to investigate the gain in precision and rare variant-capture with WGS data compared to genotyped/imputed SNPs. In total, 1005^25^ individuals with high coverage WGS data from the kinship-structured population-based Northern Swedish population health study (NSPHS)^26^ were included. This cohort has also been genotyped^27^ and imputed, making this a valuable opportunity to compare the relative performance of WGS and genotyping/imputation in relation to the same phenotype measurements. This study is one of few that uses WGS data with a GWAS approach in order to capture a greater number of low frequency variants associated with inflammatory protein biomarkers, and to further characterize the genetic structure underlying these associations, aiming to extend our knowledge of the genetic contribution to these biomarkers.

## Results

A total of 1005 individuals with WGS data and biomarker data were included in this study. The age of the participants ranged from 14 to 94 years with a median of 52 years, and 50.8% of the participants were females. The biomarkers were measured at two timepoints. At the first timepoint, biomarkers from Olink’s Oncology I and Cardiovascular I panels were measured of which 31 are inflammatory (from now on called ONC_CVD). At the second timepoint, 95 inflammatory biomarkers from the Inflammatory I panel, were measured (from now on called INF). After quality control (QC), 72 unique biomarkers remained of which 42 were only from the INF panel, while one had been measured only on ONC_CVD. As many as 29 biomarkers were included on both the INF and ONC_CVD panels and they were considered technical replicates. The average number of individual measurements per biomarker was 915 (median 929, range: 430–957) in ONC_CVD and 829 (median 871, range: 424–892) in INF.

### Genome-wide association for biomarker levels

**Figure 1:**
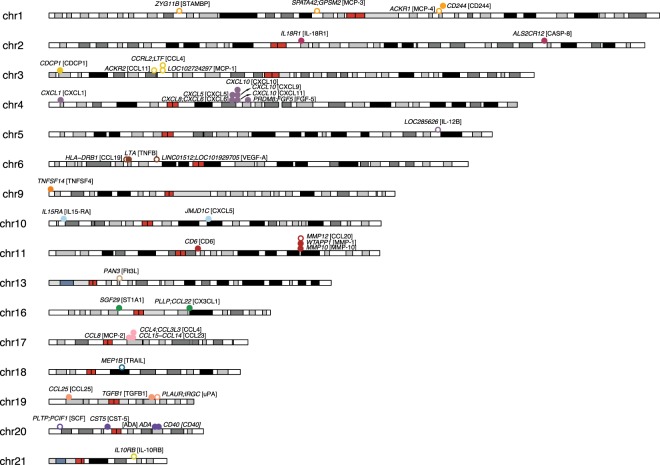
Results of GWAS analysis of the abundance of the 42 significant plasma proteins. Each dot represents a locus with a significant association. A non-filled dot represents an association in *trans* (on another chromosome than the gene encoding the biomarker) and the filled dots an association in *cis*. The dots are labelled with the names of the genes/locus that the top variant is located in in italics and the associated biomarker in brackets. Two genes are shown if it is intergenic. Red color depicts the centromere.

In the WGS data, a total of 16,890,549 biallelic single-nucleotide variants (SNVs) were called. A MAF threshold of 0.15% was chosen in order to reach enough statistical power in the GWAS (Supplementary Fig. S1). After filtering on MAF and Hardy-Weinberg equilibrium (HWE), 12,210,410 SNVs remained for downstream analyses. For the 72 individual biomarkers analyzed, 5,812 genome-wide significant (P < 1.62 × 10^−8^) associations were identified, and for 41 (56.9%) of the biomarkers, there was at least one associated SNV (Fig. 1, Table 1, Supplementary Table S1). For CCL4 and CXCL5, two independent associations each were identified, making it a total of 43 independent associations.

**Table 1:** Location and annotation of significant top GWAS hits from WGS data.

Biomarker	SNV	P-value	Effect, beta (SE)	Effect allele (ref)	MAF (effect allele)	chr:position^†^	Gene	Type	Location**
ADA	**rs11555566**	4.91 × 10^−18^	1.46 (0.17)	C (T)	0.019	20:43255220	*ADA*	missense	*cis*
CASP-8	rs116010659*	3.623 × 10^−09^	0.46 (0.07)	T (C)	0.165	2:202178477	*ALS2CR12*	intronic	*cis*
CCL11	rs2228467	2.19 × 10^−09^	0.63 (0.11)	C (T)	0.070	3:42906116	*ACKR2*	missense	*trans*
CCL19	rs149941420	4.28 × 10^−18^	0.61 (0.07)	G (T)	0.160	6:32556454	*HLA-DRB1*	intronic	*trans*
CCL20	rs17368659*	1.40 × 10^−09^	0.42 (0.07)	G (T)	0.160	11:102742761	*MMP-12*	intronic	*trans*
CCL23	**rs712048**	1.28 × 10^−12^	−0.64 (0.09)	A (C)	0.087	17:34326215	*CCL14-CCL15*	ncRNA_intronic	*cis*
CCL25	rs2032887	1.09 × 10^−37^	0.72 (0.06)	G (A)	0.301	19:8121360	*CCL25*	missense	*cis*
CCL4	rs113010081	4.19 × 10^−38^	0.80 (0.06)	C (T)	0.232	3:46457412	*CCRL2;LTF*	intergenic	*trans*
CCL4	rs4141329*	1.55 × 10^−14^	−0.38 (0.05)	C (A)	0.472	17:34490448	*CCL4;CCL3L3*	intergenic	*cis*
CD244	**rs71517284**	1.16 × 10^−13^	0.41 (0.06)	C (T)	0.378	1:160802681	*CD244*	intronic	*cis*
CD40	rs4239702*	1.01 × 10^−49^	−0.84 (0.06)	T (C)	0.273	20:44749251	*CD40*	intronic	*cis*
CD6	rs11230563	5.23 × 10^−31^	−0.79 (0.07)	T (C)	0.168	11:60776209	*CD6*	missense	*cis*
CDCP1	**rs78521038**	2.62 × 10^−12^	−0.44 (0.06)	A (G)	0.225	3:45176513	*CDCP1*	intronic	*cis*
CST-5	rs4239743	8.61 × 10^−21^	0.60 (0.06)	C (A)	0.499	20:23859017	*CST5*	intronic	*cis*
CX3CL1	rs9921681*	3.37 × 10^−10^	0.33 (0.05)	T (C)	0.309	16:57374418	*PLLP;CCL22*	intergenic	*cis*
CXCL1	rs3117604	2.46 × 10^−19^	0.50 (0.06)	C (T)	0.331	4:74734668	*CXCL1*	upstream	*cis*
CXCL10	rs11548618*	5.07 × 10^−47^	2.11 (0.15)	A (G)	0.035	4:76943947	*CXCL10*	missense	*cis*
CXCL11	rs11548618*	3.44 × 10^−13^	−1.05 (0.14)	A (G)	0.035	4:76943947	*CXCL10*	missense	*cis*
CXCL5	rs425535*	1.09 × 10^−34^	1.01 (0.08)	T (C)	0.103	4:74863997	*CXCL5*	synonymous	*cis*
CXCL5	rs10740118*	6.09 × 10^−27^	0.40 (0.05)	C (G)	0.443	10:65101207	*JMJD1C*	intronic	*trans*
CXCL6	rs111903579	6.71 × 10^−58^	0.81 (0.05)	T (C)	0.445	4:74700432	*CXCL8;CXCL6*	intergenic	*cis*
CXCL9	rs11548618*	6.19 × 10^−10^	−0.90 (0.15)	A (G)	0.035	4:76943947	*CXCL10*	missense	*cis*
FGF-5	**rs16998073**	1.50 × 10^−11^	0.44 (0.07)	T (A)	0.335	4:81184341	*PRDM8;FGF5*	intergenic	*cis*
Flt3L	rs111595024*	1.01 × 10^−16^	1.76 (0.21)	A (G)	0.015	13:28761592	*PAN3*	intronic	*trans*
IL-10RB	rs8178528	5.46 × 10^−35^	−0.64 (0.05)	A (G)	0.425	21:34660980	*IL10RB*	intronic	*cis*
IL-12B	rs10043720	7.99 × 10^−31^	−0.68 (0.06)	A (G)	0.262	5:158767333	*LOC285626*	ncRNA_intronic	*cis*
IL-15RA	rs3136630	2.64 × 10^−19^	−0.56 (0.06)	T (C)	0.312	10:5997820	*IL15RA*	intronic	*cis*
IL-18R1	rs10190555	2.37 × 10^−72^	1.08 (0.06)	A (G)	0.233	2:102994056	*IL18R1*	intronic	*cis*
TGFB1	rs1800472*	1.35 × 10^−12^	−0.88 (0.12)	A (G)	0.040	19:41847860	*TGFB1*	missense	*cis*
MCP-1	rs1800024*	1.26 × 10^−09^	0.58 (0.10)	T (C)	0.075	3:46412559	*LOC102724297*	ncRNA_intronic	*trans*
MCP-2	rs1133763	2.693 × 10^−53^	−1.30 (0.08)	C (A)	0.104	17:32647831	*CCL8*	missense	*cis*
MCP-3	**rs11102571**	1.34 × 10^−09^	0.50 (0.08)	C (G)	0.112	1:109407135	*SPATA42;GPSM2*	intergenic	*trans*
MCP-4	rs12075	1.25 × 10^−45^	−0.72 (0.05)	G (A)	0.474	1:159175354	*ACKR1*	missense	*trans*
MMP-1	rs471994*	5.02 × 10^−19^	−0.47 (0.05)	A (G)	0.390	11:102697731	*WTAPP1*	ncRNA_intronic	*cis*
MMP-10	rs17359286*	1.17 × 10^−08^	−0.51 (0.09)	T (G)	0.081	11:102643718	*MMP-10*	synonymous	*cis*
SCF	rs6073958*	1.20 × 10^−09^	0.037 (0.06)	C (T)	0.199	20:44551855	*PLTP;PCIF1*	intergenic	*trans*
ST1A1	**rs138534121**	2.51 × 10^−13^	0.78 (0.11)	G (A)	0.064	16:28595989	*SGF29*	intronic	*cis*
STAMBP	**1:53206258**^§^	3.32 × 10^−09^	0.92 (0.16)	T (G)	0.026	1:53206258	*ZYG11B*	intronic	*trans*
TNFB	**rs2229092**	2.70 × 10^−29^	−1.77 (0.16)	C (A)	0.027	6:31540757	*LTA*	missense	*cis*
TNFSF14	rs344560	3.72 × 10^−17^	−0.88 (0.10)	T (C)	0.065	19:665020	*TNFSF14*	missense	*cis*
TRAIL	rs144242131*	1.02 × 10^−12^	1.98 (0.28)	A (G)	0.007	18:29769910	*MEP1B*	upstream	*trans*
uPA	**rs346058**	7.11 × 10^−09^	−0.71 (0.12)	T (A)	0.046	19:44202855	*PLAUR;IRGC*	intergenic	*trans*
VEGF-A	rs6921438	1.63 × 10^−12^	0.35 (0.05)	G (A)	0.434	6:43925607	*LINC01512;* *LOC101929705*	intergenic	*cis*

The raw p-values (not adjusted for multiple testing) are shown. If one biomarker had been measured twice (i.e. been measured on both INF and ONC_CVD), the SNV with the most significant p-value is presented. Novel variants are shown in bold. Additional information can be found in Supplementary Table S2.

^§^Does not have an rs-id,

^†^In hg19 coordinates

^*^Variant is from ONC_CVD. Either the p-value was lower, or no significant association was found in INF.

^**^In *cis:* within 1 Mb of the gene encoding the biomarker; in *trans:* on another chromosome of the gene encoding the biomarker.

We identified 11 biomarkers that had significant associations in both ONC_CVD and INF, representing 1,418 SNV-biomarker associations. Seven of the biomarkers had significant associations only when analyzing the measurements from ONC_CVD, but not when analyzing the same biomarker measured on the INF panel. However, these variants had p-values just below the genome-wide threshold (ranging from 1.17 × 10^−8^ to 3.43 × 10^−13^) in ONC_CVD and p-values just above the genome-wide threshold in INF (Supplementary Table S2). Here, the larger sample size in ONC_CVD (90–100 more individuals) probably increased the power enough to reach genome-wide significance.

**Figure 2:**
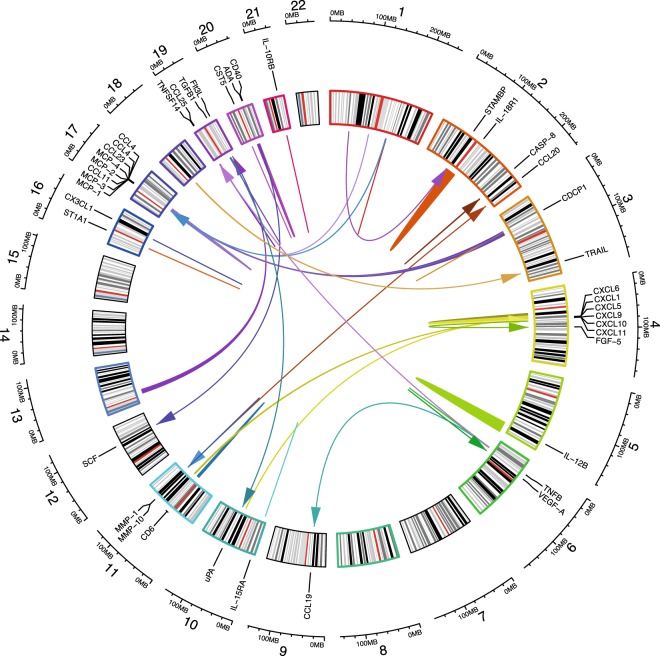
Circular representation of the GWAS hits. The numbers in the outer circle correspond to the chromosomes. Each biomarker is labelled at the position of the gene coding it on the cytoband. The colored lines/arrows represent the significant hits. The breadth of the line represents the size of the region associated with respective biomarker.

Most biomarkers (67.44%) with at least one significant hit identified, had an association in *cis* (i.e., within 1 Mb of the gene encoding the biomarker) or even within the gene encoding the biomarker itself. The rest of the associations were in *trans*, all located on another chromosome than the gene encoding the biomarker (Fig. 2). Adjusting for the most significant SNV resulted in 15 biomarkers having a secondary, significant signal close to the primary signal, and adjusting for both the primary and secondary SNV resulted in seven biomarkers having a tertiary signal (Table 2, with more extensive variant data in Supplementary Tables S3 and S4). In the conditional analyses, only the SNVs that were located within each associated region (see methods) were analyzed. Due to the reduced number of variants analyzed in the conditional analyses, as compared to the primary GWAS, the power to analyze rarer variants increased and we therefore did not have a MAF threshold in the conditional analyses. Here, we then identified four variants with MAF < 0.15%

**Table 2:** Location and annotation of top GWAS hits after having conditioned on the most significant hit.

Biomarker	SNV	Conditional signal	P-value	P adj.	Effect, beta (SE)	Effect allele (ref)	MAF (effect allele)	chr:position^†^	Gene	Type	Location**
CCL23	rs72831705	secondary	9.52 × 10^−11^	1.07 × 10^−05^	0.44 (0.07)	T (C)	0.153	17:34321277	*CCL15-CCL14*	ncRNA_intronic	*cis*
CCL23	rs854671	tertiary	1.48 × 10^−08^	1.67 × 10^−03^	0.23 (0.05)	C (T)	0.475	17:34361300	*CCL23;* *CCL18*	intergenic	*cis*
CCL4	3:51599851^§^*	secondary	3.53 × 10^−07^	2.10 × 10^−02^	3.09 (0.61)	A (C)	0.00098	3:51599851	*RAD54L2*	intronic	*trans*
CCL4	rs188700215*	secondary	1.01 × 10^−07^	5.91 × 10^−03^	−5.19 (0.97)	A (G)	0.00098	17:30092085	*MIR365B;COPRS*	intergenic	*trans*
CCL4	rs201079256*	tertiary	1.02 × 10^−13^	5.97 × 10^−09^	−0.36 (0.05)	T (C)	0.465	17:34522125	*CCL3L1;* *CCL3L3*	downstream	*cis*
CD40	rs6063068*	secondary	7.41 × 10^−08^	6.27 × 10^−03^	3.28 (0.61)	T (A)	0.00098	20:45717496	*EYA2*	intronic	*cis*
CD40	rs182282247*	tertiary	9.99 × 10^−08^	8.45 × 10^−03^	0.82 (0.15)	A (G)	0.021	20:44730041	*NCOA5;* *CD40*	intergenic	*cis*
CST-5	rs6138152	secondary	3.87 × 10^−07^	3.13 × 10^−02^	0.36 (0.07)	G (A)	0.211	20:23850130	*CST2;CST5*	intergenic	*cis*
CST-5	rs75823487	tertiary	4.56 × 10^−07^	3.69 × 10^−02^	4.25 (0.84)	T (C)	0.0015	20:29478349	*MIR663AHG;* *LINC01597*	intergenic	*trans*
CXCL1	rs10938101*	secondary	7.53 × 10^−07^	6.54 × 10^−03^	−0.24 (0.05)	T (G)	0.461	4:74688772	*CXCL8;CXCL6*	intergenic	*cis*
CXCL6	rs181216093*	secondary	5.27 × 10^−09^	8.42 × 10^−04^	−1.21 (0.21)	T (C)	0.009	4:74661204	*CXCL8;CXCL6*	intergenic	*cis*
IL-15RA	rs144173272	secondary	1.49 × 10^−11^	1.86 × 10^−06^	−1.70 (0.25)	T (C)	0.013	10:6008255	*IL15RA*	missense	*cis*
IL-15RA	rs35095871	tertiary	3.06 × 10^−07^	3.81 × 10^−02^	0.41 (0.08)	G (A)	0.102	10:5700416	*ASB13*	intronic	*cis*
IL-18R1	rs12999517	secondary	4.89 × 10^−19^	1.31 × 10^−13^	−0.47 (0.05)	C (T)	0.172	2:102959260	*IL1RL1*	intronic	*cis*
MCP-2	rs74832623	secondary	6.47 × 10^−32^	7.79 × 10^−27^	−1.17 (0.10)	G (A)	0.045	17:32535173	*LINC01989;CCL2*	intergenic	*cis*
MCP-2	rs12601658	tertiary	7.15 × 10^−12^	8.61 × 10^−07^	−0.32 (0.05)	A (T)	0.244	17:32533423	*LINC01989;* *CCL2*	intergenic	*cis*
MMP-1	rs470358*	secondary	9.38 × 10^−09^	1.56 × 10^−03^	0.29 (0.05)	T (C)	0.397	11:102668702	*WTAPP1*	ncRNA_intronic	*cis*
SCF	rs6104417*	secondary	2.66 × 10^−07^	2.53 × 10^−02^	0.23 (0.05)	C (T)	0.4995	20:44632542	*ZNF335;MMP9*	intergenic	*trans*
ST1A1	rs4149383	secondary	5.38 × 10^−07^	4.56 × 10^−02^	0.54 (0.11)	A (G)	0.061	16:28620320	*SULT1A1*	UTR5	*cis*
TNFB	rs746868	secondary	1.42 × 10^−07^	2.18 × 10^−02^	0.26 (0.05)	C (G)	0.061	6:31540429	*TNFB*	intronic	*cis*
TNFB	6:27190519^§^	tertiary	4.06 × 10^−08^	6.22 × 10^−03^	1.77 (0.32)	T (G)	0.005	6:27190519	*MIR3142;* *PRSS16*	intergenic	*trans*
TNFSF14	rs2291668	secondary	4.71 × 10^−07^	7.36 × 10^−03^	0.27 (0.05)	A (G)	0.281	19:6669934	*TNFSF14*	synonymous	*cis*
TRAIL	18:21026109^§^*	secondary	1.01 × 10^−07^	1.28 × 10^−02^	−5.19 (0.97)	A (G)	0.00049	18:21026109	*TMEM241;RIOK3*	intergenic	*trans*

The raw p-values (not adjusted for multiple testing) are shown. The adjusted p-values are based on the number of SNVs tested in each region which means that each SNV does not need to reach genome wide significance. If one biomarker had been measured twice (i.e. been measured on both INF and ONC_CVD), the SNV with the most significant p-value is presented. Additional information can be found in Supplementary Table S4.

^§^Does not have an rs-id,

^†^In hg19 coordinates.

^*^Variant is from ONC_CVD. Either the p-value was lower, or no significant association was found in INF.

^**^In *cis:* within 1 Mb of the gene encoding the biomarker; in *trans:* on another chromosome of the gene encoding the biomarker.

**Figure 3:**
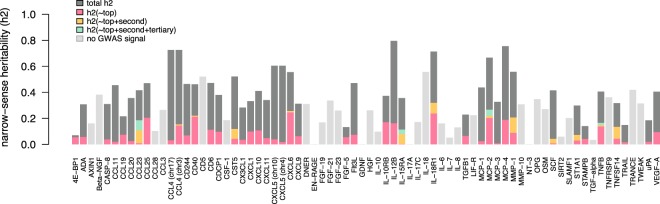
Narrow-sense heritability estimates of the top variants. The total heritability estimate is shown in dark grey. The contribution of the top variant is shown in pink, the contribution of the first conditional top variant (secondary hit) in yellow and the second conditional (tertiary hit) in green. Light grey depicts biomarkers with no significant GWAS signal.

In general, the biomarkers without a genome-wide significant association had heritability estimates below 0.3, i.e. less than 30% of the variation in biomarker abundance is due to genetic factors (Supplementary Table S5). For many GWAS-associated biomarkers, the heritability was still fairly high, with the top SNVs and the top conditional SNVs accounting for a total of 5–20% of the total variance in biomarker abundance in most cases (Fig. 3).

### Comparison with our previous GWAS using genotyped/imputed data suggests novel loci for many biomarkers

Twenty of the biomarkers (ADA, CASP-8, CCL11, CCL20, CCL23, CD244, CDCP1, CST5, CX3CL1, CXCL1, CXCL11, CXCL9, FGF-5, MCP-3, ST1A1, STAMBP, TGFB1, TNFB, TNFSF14, uPA) with significant associations in the present study, did not have any significant associations in our previous GWAS when using genotyped/imputed SNP data^25,28^ (Supplementary Figs S2–S20). The abundance of two of these biomarkers (CXCL9 and CXC11) had an associated variant that in our previous studies was identified to be associated only with CXCL10 and is most likely a false positive finding for CXCL9 and CXCL11 (discussed more thoroughly in Supplementary, including Supplementary Figs S21–S25). The remaining novel biomarker associations represented 18 unique loci that were not found to be associated with the levels of the same biomarker using genotyped/imputed data in the same cohort. Of these, 15 loci (see overlap with GWAS catalog below) have not been reported in any previous study of the same biomarkers, thus making them novel loci. In the novel loci, six top variants are considered to be low-frequency variants (MAF < 5%). Additional to the novel loci, four biomarkers (CD6, CXCL5, CCL4, MMP-10) had associations driven by top variants that are only in moderate in LD (R^2^ < 0.8) with the top variants from our previous studies, and might therefore be considered independent associations (Supplementary Figs S26–S30). Another 19 loci overlapped between the present study and our previous studies with SNP data^25,28^, for which nine loci had the same top variant. The remaining ten overlapping loci had different top variants, although these variants were in high LD (R^2^ > 0.8). The top variants in the overlapping loci were more strongly associated (more significant p-value) in the present study than in our previous GWAS, except for two biomarkers (MMP-10 and TRAIL), for which more significant GWAS top variants were found in the previous studies (Tables 3 and 4).

**Table 3:** Top GWAS hits with WGS data in comparison to the significant genotyped/imputed associations identified by Ahsan *et al*.^25^.

Biomarker	WGS top variant	chr:pos^†^	MAF WGS (effect allele)	MAF imputed (effect allele)	Effect allele (ref)	Genotype quality (sd) WGS	Imputation quality for WGS variant	P present	P Ahsan^25^ for WGS top variant	Ahsan top variant (R^2§^)^25^	P top variant Ahsan^25^
CCL19	rs149941420*	6:32556454	0.160	0.125	G (T)	90.92 (13.34)	0.846	4.269 × 10^−18^	5.429 × 10^−12^	rs2395201 (0.277)	5.951 × 10^−17^
CCL4	rs113010081	3:46457412	0.232	0.201	C (T)	92.44 (11.21)	0.996	4.188 × 10^−38^	3.124 × 10^−23^	rs113341849 (0.992)	3.326 × 10^−26^
CCL4	rs4141329	17:34490448	0.472	0.483	C (A)	94.92 (10.96)	0.712	1.550 × 10^−14^	n.s.^‡^	rs113877493 (0.095)	9.181 × 10^−10^
CD40	rs4239702	20:44749251	0.273	0.261	T (C)	97.09 (6.08)	0.996	1.014 × 10^−49^	3.288 × 10^−18^	rs4810485 (0.911)	4.697 × 10^−19^
CXCL10	rs11548618	4:76943947	0.035	0.035	A (G)	92.21 (10.60)	1	5.072 × 10^−47^	2.132 × 10^−37^	rs11548618 (1)	2.132 × 10^−37^
CXCL5	rs425535	4:74863997	0.103	0.100	T (C)	92.63 (10.84)	0.989	1.091 × 10^−34^	2.081 × 10^−25^	rs425535 (1)	2.081 × 10^−25^
CXCL5	rs7088799	10:65016174	0.443	0.446	G (T)	97.22 (6.00)	0.999	7.357 × 10^−16^	4.598 × 10^−11^	rs7896910 (0.735)	2.932 × 10^−11^
CXCL6	rs111903579	4:74700432	0.446	NA**	T (C)	85.04 (18.31)	NA**	6.708 × 10^−58^	NA**	rs16850073 (1)	1.976 × 10^−32^
Flt3L	rs111595024	13:28768589	0.015	NA**	G (A)	50.08 (21.35)	NA**	1.008 × 10^−16^	NA**	rs145096717 (0.967)	3.045 × 10^−14^
MCP-1	rs1800024	3:46412559	0.075	0.077	T (C)	93.57 (9.38)	0.998	1.257 × 10^−09^	n.s. ^‡^	rs2888526 (0.979)	2.399 × 10^−09^
MMP-1	rs471994	11:102697731	0.389	0.395	A (G)	94.33 (10.16)	1	5.017 × 10^−19^	1.736 × 10^−15^	rs471994 (1)	1.736 × 10^−15^
MMP-10	rs17359286	11:102643718	0.081	0.058	T (G)	93.56 (9.98)	0.999	1.171 × 10^−08^	n.s. ^‡^	rs486055 (0.583)	9.246 × 10^−10^
SCF	rs6073958	20:44551855	0.199	0.196	C (T)	96.37 (7.18)	0.997	1.204 × 10^−09^	2.325 × 10^−09^	rs6073958 (1)	2.325 × 10^−09^
TRAIL	rs144242131	18:29769910	0.007	0.007	A (G)	90.74 (12.23)	0.999	1.020 × 10^−12^	1.387 × 10^−16^	rs144242131 (1)	1.387 × 10^−16^

Results from ONC_CVD are compared*. The p-values for the top variants from the present study are shown (P present) as well as the p-values for the same variant in the imputed data (P Ahsan for WGS top variant). The most significant SNV from the previous study and corresponding p-value is also shown, and its LD (R^2^) with the most significant SNV from the present study. The comparisons have been filtered the same way as the present study: only biallelic variants and variants not located in a spanning deletion are compared.

^†^In hg19 coordinates.

^‡^Not significant in Ahsan *et al*. (P > 4.2e-09).

^§^R^2^ with WGS top variant.

^*^All top variants and P-values are from the analyses of the ONC_CVD panel, except for CCL19 that is from INF.

^**^Did not pass imputation QC or were not present in the reference panel used for the imputations.

**Table 4:** Top GWAS hits from WGS data in comparison to the significant genotyped/imputed associations identified by Enroth *et al*.^28^.

Biomarker	WGS top variant	chr:pos^†^	MAF WGS (effect allele)	MAF imputed (effect allele)	Effect allele (ref)	Genotype quality (sd) WGS	Imputation quality of WGS top variant	P present	P Enroth^28^ for WGS top variant	Enroth top variant (R^2§^)^28^	P top variant Enroth^28^
CCL19	rs149941420	6:32556454	0.160	0.125	G (T)	90.92 (13.34)	0.846	4.269 × 10^−18^	n.s. ^‡^	rs9968904 (0.979)	5.744 × 10^−13^
CCL25	rs2032887	19:8121360	0.301	0.302	G (A)	92.07 (12.10)	1	1.089 × 10^−37^	4.368 × 10^−35^	rs2032887 (1)	4.368 × 10^−35^
CCL4	rs113010081	3:46457412	0.232	0.201	C (T)	92.44 (11.21)	0.996	4.188 × 10^−38^	7.834 × 10^−24^	rs113341849 (0.992)	7.834 × 10^−24^
CD40	rs1569723	20:44742064	0.256	0.257	C (A)	95.30 (8.69)	1	5.242 × 10^−43^	6.608 × 10^−21^	rs4810485 (0.997)	4.960 × 10^−21^
CD6	rs11230563	11:60776209	0.168	0.164	T (C)	90.08 (13.40)	1	5.235 × 10^−31^	1.115 × 10^−18^	rs11230556 (0.729)	9.259 × 10^−21^
CXCL10	rs11548618	4:76943947	0.035	0.035	A (G)	92.21 (10.60)	1	5.072 × 10^−47^	5.396 × 10^−37^	rs11548618 (1)	5.396 × 10^−37^
CXCL5	rs352045	4:74864687	0.103	0.100	T (G)	89.77 (13.54)	0.995	6.091 × 10^−27^	1.164 × 10^−19^	rs2564594 (0.974)	7.826 × 10^−20^
CXCL5	rs10740118	10:65101207	0.443	0.445	C (G)	96.52 (7.24)	0.999	2.927 × 10^−13^	5.033 × 10^−10^	rs12770839 (0.698)	8.833 × 10^−12^
CXCL6	rs111903579*	4:74700432	0.446	NA**	T (C)	85.04 (18.31)	NA**	6.708 × 10^−58^	NA**	rs6831029 (0.813)	1.126 × 10^−26^
Flt3L	rs145096717	13:28761592	0.015	0.002	A (G)	94.67 (8.47)	0.668	2.086 × 10^−16^	3.519 × 10^−14^	rs145096717 (1)	3.519 × 10^−14^
IL-10RB	rs8178528	21:34660980	0.425	0.423	A (G)	95.79 (8.07)	0.968	5.461 × 10^−35^	n.s. ^‡^	rs2843697 (0.951)	1.098 × 10^−16^
IL-12B	rs10043720	5:158767333	0.272	0.269	A (G)	91.15 (12.75)	0.998	7.988 × 10^−31^	1.424 × 10^−17^	rs10076557 (1)	4.247 × 10^−18^
IL-15RA	rs3136630	10:5997820	0.312	0.312	T (C)	91.43 (12.75)	1	2.644 × 10^−19^	1.659 × 10^−11^	rs3136630 (1)	1.659 × 10^−11^
IL-18R1	rs10190555	2:102994056	0.233	0.234	A (G)	94.92 (9.44)	0.999	2.373 × 10^−72^	1.155 × 10^−51^	rs2058660 (0.957)	5.500 × 10^−51^
MCP-2	rs1133763	17:32647831	0.104	0.109	C (A)	91.07 (12.04)	0.978	2.693 × 10^−53^	n.s. ^‡^	rs3138037 (1)	2.113 × 10^−48^
MCP-4	rs12075	1:159175354	0.474	0.473	G (A)	94.55 (10.13)	1	1.253 × 10^−45^	1.475 × 10^−43^	rs12075 (1)	1.475 × 10^−43^
VEGF-A	rs6921438	6:43925607	0.434	0.389	G (A)	95.10 (10.09)	0.770	8.294 × 10^−40^	n.s. ^‡^	rs7767396 (0.942)	8.048 × 10^−19^

Results from INF are compared*. The p-values for the top variants from the present study are shown (P present) as well as the p-values for the same variant in the imputed data (P Enroth for WGS top variant). The most significant SNV from the previous study and corresponding p-value is also shown, and its LD (R^2^) with the most significant SNV from the present study. The comparisons have been filtered the same way as the present study. Only biallelic variants and variants not located in a spanning deletion are compared.

^†^In hg19 coordinates.

^‡^Not significant in Enroth *et al*. (P > 4.79e-9).

^§^R^2^ with WGS top variant.

^*^For CXCL6 the variant is from ONC_CVD since this p-value was lower.

^**^Did not pass imputation QC or were not present in the reference panel used for the imputations.

### Replication in an independent cohort

Due to the lack of a similar dataset (WGS data and measured levels of the same inflammatory biomarkers) for replication, we could only test for replication for a subset of our results using GWAS results of circulating cytokines in a Finnish population^29^ (Supplementary Table S6). Of the cytokines that were analyzed in both studies, we fully replicated our primary results for CCL11, i.e., the same top SNV was found in both cohorts. One of the two independent associations with CCL4 was also fully replicated. We also replicated one of the three independent associations with MCP-1, and one of the two independent associations with CXCL1, although with an LD between the top variants of R^2^ = 0.75 and 0.72 for MCP-1 and CXCL1, respectively. For our second independent association with CCL4, our top variant was either monomorphic in the Finnish population or had not been analyzed. However, the most significant CCL4 associated SNV in the Finnish population is also genome-wide significant in our study (P = 3.52 × 10^−12^), even if not our most significant. Our results for MCP-3, SCF and CXCL9 did not replicate in the Finnish population (P > 0.05 in the Finnish cohort for our top SNVs) and our result for TNFB was only nominally significant (P = 0.027). On the other hand, the most significant SNVs for IL-7, IL-10, IL-18 and HGF in the Finnish population, were not genome-wide significant in our cohort, even if rs5745687 (for HGF) as well as rs385076, rs17229943, and rs71478720 (for IL-18) were nominally significant (P < 0.05).

### Colocalization with eQTL data in blood

Colocalization (the same top variant) with *cis*-eQTLs in peripheral blood was found for five of the top SNVs (Table 5), associated with the levels of three different biomarkers (CD40, CXCL5 and IL-15RA). For the other top variants from our biomarker GWAS, LD was calculated between each top variant and the most significant eQTL. Two top variants, associated with CXCL5, were colocalized (R^2^ > 0.8) with a variant associated with a *trans-*eQTL in blood. Two secondary hits associated with IL-18R1 and TNFSF14 respectively, and one tertiary hit associated with CCL23, were also found to be colocalized with a *cis*-eQTL. All overlapping variants had the same direction of effect except for the overlap with the *trans-*eQTL, where two SNVs (rs10740118 and rs7088799) in *JMJD1C* was associated with increased protein levels of *CXCL5*, but was in LD (R^2^ = 0.86) with a variant, rs10761779 that was associated with decreased RNA levels of *CXCL5*.

**Table 5:** Overlapping top SNVs from our biomarker GWAS with WGS data and top SNVs from the eQTL analyses by Westra *et al*.^51^.

Gene name	Biomarker	Top SNV (biomarker GWAS)	Annotation	Top SNV (eQTL)	LD	P (biomarker GWAS)	P (eQTL)
*CD40*	CD40 (INF)	rs1569723	intergenic (*NCOA5*;*CD40*)	rs1569723	1	5.24 × 10^−43^	1.06 × 10^−28^
*CD40*	CD40 (ONC_CVD)	rs4239702	intronic (*CD40*)	rs4239702	1	1.01 × 10^−49^	1.26 × 10^−34^
*CXCL5*	CXCL5 (INF)	rs352045 (*cis*)*	upstream (*CXCL5*)	rs352045	1	6.09 × 10^−27^	4.25 × 10^−111^
*CXCL5*	CXCL5 (ONC_CVD)	rs425535 (*cis*)*	exonic (*CXCL5*)	rs425535	1	1.09 × 10^−34^	4.50 × 10^−111^
*IL15RA*	IL-15RA	rs3136630	intronic (*IL15RA*)	rs3136630	1	2.64 × 10^−19^	5.21 × 10^−6^
*CXCL5*	CXCL5 (INF)	rs10740118 (*trans*)**	intronic (*JMJD1C*)	rs10761779	0.856	2.93 × 10^−13^	1.82 × 10^−7^
*CXCL5*	CXCL5 (ONC_CVD)	rs7088799 (*trans*)**	intronic (*JMJD1C*)	rs10761779	0.856	7.36 × 10^−16^	1.82 × 10^−7^
*IL18R1*	IL-18R1	rs12999517^†^	intronic (*IL1RL1*)	rs12999517	1	4.89 × 10^−19^	1.13 × 10^−39^
*TNFSF14*	TNFSF14	rs2291668^†^	synonymous (*TNFSF14*)	rs1077667	0.899	4.71 × 10^−07^	4.36 × 10^−47^
*CCL23*	CCL23	rs854671^‡^	intergenic (*CCL23*;*CCL18*)	rs854671	1	1.48 × 10^−08^	3.21 × 10^−27^

Linkage disequilibrium (R^2^) is presented for biomarkers that had different top SNVs in ONC_CVD and INF, but are both in LD with a top eQTL.

^†^From the conditional analysis, adjusted for the top variant.

^‡^From the second conditional analysis, adjusted for the top primary and secondary variant.

^*^Top variant in *cis*, within 1 Mb of the gene encoding the biomarker.

^**^Top variant in *trans*, on another chromosome than the gene encoding the biomarker.

### Colocalization with data from the GWAS catalog

The association signal for eight biomarkers (CCL19, CCL4, CD40, CD6, CXCL5, IL-12B, IL-18R1, TNFSF14) colocalized with association signals for one or several inflammatory diseases (Table 6, Supplementary Table S7). Here, we regard the signals to be colocalized when a top SNV or any SNVs in LD (R^2^ > 0.8) with a top SNV identified in our study was also the top SNV for an inflammatory disease in the GWAS catalog (v 1.0.2). In four cases, our top SNV had been associated with an inflammatory disease in previous GWAS: rs113010081/CCL4 with inflammatory bowel disease, rs1569723/CD40 with Crohn’s disease, rs4239702/CD40 with rheumatoid arthritis and rs11230563/CD6 with ulcerative colitis. The remaining top variants are in LD (R^2^ > 0.8) with previously inflammatory disease-associated top variants. For example, the top variants for CXCL5 found in this study (rs352045 in ONC_CVD and rs425535 in INF) are both in high LD (R^2^ = 0.944) with a variant previously associated with ulcerative colitis. Some of our top SNVs were also colocalized with associations for different blood-trait (Supplementary Table S8).

**Table 6:** Disease-associations for the inflammatory biomarkers.

Biomarker	Disease trait	Mapped trait	SNV (biomarker GWAS)	Annotation (biomarker GWAS)	Associated SNP (GWAS catalog)	LD^§^
CCL19	Asthma, Juvenile idiopathic arthritis, Rheumatoid arthritis	Asthma; systemic, polyarticular, rheumatoid factor negative, oligoarticular juvenile idiopathic arthritis; Rheumatoid arthritis	rs149941420	intronic (*HLA-DRB1*)	rs7775228	0.849
CCL4	Inflammatory bowel disease, Juvenile arthritis, Ulcerative colitis	Inflammatory bowel disease; systemic, polyarticular, rheumatoid factor negative, oligoarticular juvenile idiopathic arthritis; Ulcerative colitis	**rs113010081**	intergenic (*CCRL2*;*LTF*)	rs113010081	1
CD40	Chronic hepatitis B infection, Chronic inflammatory diseases, Crohn’s disease, Inflammatory bowel disease, Kawasaki disease, Multiple sclerosis, Rheumatoid arthritis, Systemic lupus erythematosus	Chronic hepatitis B infection; Ankylosing spondylitis; Psoriasis; Ulcerative colitis; Sclerosing cholangitis; Crohn’s disease; Inflammatory bowel disease; Mucocutaneous lymph node syndrome; Multiple sclerosis; Rheumatoid arthritis; Systemic lupus erythematosus	**rs1569723**, **rs4239702**	intergenic (*NCOA5*;*CD40*), intronic (*CD40*)	rs1569723, rs4239702, rs1883832, rs1569723, rs6074022, rs2425752, rs4810485, rs6032662	1, 1, 0.914, 0.914, 0.914, 0.843, 0.906, 0.914
CD6	Chronic inflammatory diseases, Crohn’s disease, Inflammatory bowel disease, Ulcerative colitis	Ankylosing spondylitis; Psoriasis; Ulcerative colitis; Sclerosing cholangitis; Crohn’s disease; Inflammatory bowel disease	**rs11230563**	missense (*CD6*)	rs11230563	1
CXCL5	Ulcerative colitis	Ulcerative colitis	rs352045, rs425535	upstream (*CXCL5*), exonic (*CXCL5*)	rs2457996	0.944
IL-12B	Ankylosing spondylitis, Chronic inflammatory diseases, Crohn’s disease	Ankylosing spondylitis; Psoriasis; Ulcerative colitis; Sclerosing cholangitis; Crohn’s disease	rs10043720	ncRNA intronic (LOC285626)	rs6556416, rs6556411, rs10045431	0.993, 1, 0.884
IL-18R1	Celiac disease, Crohn’s disease, Inflammatory bowel disease, Pediatric autoimmune diseases	Celiac disease; Crohn’s disease; Inflammatory bowel disease; Autoimmune thyroid disease; Type I diabetes mellitus; Common variable immunodeficiency, Chronic childhood arthritis; Ankylosing spondylitis; Psoriasis; Ulcerative colitis; Autoimmune disease; Systemic lupus erythematosus	rs10190555	intronic (*IL18R1*)	rs13015714, rs917997, rs990171, rs2058660, rs6708413, rs2075184	0.991, 0.954, 0,954, 0.954, 0.954, 0.954
TNFSF14	Multiple sclerosis	Multiple sclerosis	rs2291668 (secondary)	synonymous (*TNFSF14*)	rs1077667	0.879

Disease-associations with inflammatory diseases in the GWAS catalog are presented. If the variant has been reported in the catalog before, it is marked in bold. The other have not been previously reported, but are in strong LD with variants that have (R^2^ > 0.8). A more extensive Table is found in Supplementary material, Supplementary Table S6.

^§^R^2^ between our SNV and the previously associated variants from the GWAS catalog.

## Discussion

We performed a GWAS on 72 inflammatory biomarkers in a Swedish cohort using WGS data, and identified SNVs that were associated with the plasma levels for as many as 41 biomarkers. Of the biomarkers with at least one significant hit, 67.44% had an association within 1 Mb of the gene encoding the biomarker (in *cis*) and the rest (32.56%) had an association on another chromosome (in *trans*). Many of the biomarker levels are highly heritable and some top SNVs explained as much as 25% of the variability. When comparing the results to our previous GWA analyses^25,28^ using genotyped/imputed data, novel associations were identified for 18 biomarkers when WGS data was used, 15 of which has not been identified in any previous study. Additionally, in four of the biomarkers, for which the associated loci overlapped with our previous study, the top variants in the present and former studies were not correlated (R^2^ < 0.8), thus making these findings potentially independent associations.

We have previously used both mass spectrometry and the recently developed protein extension assay (PEA) to identify the genetic contribution to variation in protein levels in the NSPHS cohort, where we showed that more than 30% of the biomarkers are influenced by genetic variants^23,28,30,31^. In a recent study with a larger sample size^32^ (N = 3,394), but also based on genotyped/imputed data, we contributed to the identification of 79 genome-significant loci for 83 plasma protein biomarkers for cardiovascular disease. A more recent study by Sun *et al*.^33^ identified nearly two thousand genetic associations with almost 1,500 proteins, which increased the existing knowledge about the human plasma proteome by fourfold. With the use of WGS data, we can extend our knowledge even further. In the present study, we have shown that we can increase the power in identifying novel loci by using WGS data in GWAS, instead of using genotyped or imputed SNPs. We were able to identify associations for 58% of the biomarkers, which is a considerable higher fraction compared to the 30% identified in the same cohort using genotyped/imputed data.

In addition to a gain of power, we have also shown that we can increase the precision by using WGS data instead of genotype/imputed SNP data^25,28^. Overall, MAF agreed well between the genotyped/imputed dataset and the WGS dataset, for all associated SNVs. The MAF threshold in our previous studies was set to at least one chromosome in the dataset, which corresponds to a lower threshold than 0.15%. This means that even the rarest variants in the present study were included in the previous analyses with genotyped or imputed SNPs, even though there was no power to identify an association with such rare variants. For the 18 associations not found in our previous studies, the imputation quality was overall good, except for the SNVs that did not pass imputation QC (Table 7). In some cases, the associations were just below the significance threshold in the imputed data. Some such examples are ADA, CCL11 and TGFB1 where the top variants in the present study are suggestive hits in our previous study. These three associations are in regions with not many variants genotyped or imputed, but with many SNVs called in the WGS. For example, in the WGS analyses, a missense SNV (rs11555566) within *ADA*, which encodes adenosine deaminase, was strongly associated with the expression level of the corresponding adenosine deaminase protein (P = 4.9 × 10^−19^). This variant is quite rare in our cohort (MAF = 1.9%) and was not identified as genome-wide significant in the imputed data, even if imputation quality was suggested as good and the MAF was similar to the WGS data (Supplementary Fig. S2). In the cases of CD244, CDCP1 and ST1A1 on the other hand, the top SNVs from the present study were not imputed in our previous study (CD244), or did not pass imputation QC (CDCP1 and ST1A1). These can therefore all be considered novel associations. However, in the case of STAMBP, the genotype quality in the WGS data is also low, making this the most uncertain association. Excluding this variant, all novel top variants had a genotype quality of at least 75 in the WGS data, and are therefore considered quite robust.

**Table 7:** Location and annotation of novel top GWAS hits from the present WGS associations that were not reported in our previous studies with genotyped/imputed data.

Biomarker	chr:position^†^	SNV	MAF WGS (effect allele)	MAF imputed (effect allele)	Genotype quality (sd) WGS	Imputation quality	P WGS	P imputed^28^
ADA	20:43255220	rs11555566	0.019	0.019	86.50 (14.78)	1	4.91 × 10^−18^	1.35 × 10^−08^
CASP-8	2:202178477	rs116010659	0.165	0.141	90.00 (12.94)	0.968	3.63 × 10^−09^	7.55 × 10^−03^
CCL11	3:42906116	rs2228467	0.070	0.069	87.10 (14.11)	1	2.19 × 10^−09^	2.93 × 10^−08^
CCL20	11:102742761	rs17368659	0.160	0.154	96.12 (7.33)	0.999	1.40 × 10^−09^	9.42 × 10^−01^
CCL23	17:34326215	rs712048***	0.087	0.085	95.65 (7.46)	0.993	1.28 × 10^−12^	7.90 × 10^−11^***
CD244	1:160802681	rs71517284	0.378	NA**	91.14 (13.84)	NA**	1.16 × 10^−13^	NA**
CDCP1	3:45176513	rs78521038	0.225	NA**	94.40 (9.59)	NA**	2.62 × 10^−12^	NA**
CST-5	20:23859017	rs4239743	0.499	0.494	94.61 (9.83)	0.990	8.61 × 10^−21^	Biomarker not analysed^28^
CX3CL1	16:57374418	rs9921681	0.309	0.366	82.93 (18.48)	0.984	3.37 × 10^−10^	4.00 × 10^−06^
CXCL1	4:74734668	rs3117604	0.331	0.321	91.77 (12.80)	0.989	2.46 × 10^−19^	3.80 × 10^−01^
CXCL11*	4:76943947	rs11548618	0.035	0.034	92.21 (10.60)	1	3.44 × 10^−13^	8.96 × 10^−01^
CXCL9*	4:76943947	rs11548618	0.035	0.035	92.21 (10.60**)**	1	6.19 × 10^−10^	4.33 × 10^−01^
FGF-5	4:81184341	rs16998073	0.335	0.334	93.58 (11.26)	1	1.50 × 10^−11^	2.03 × 10^−06^
MCP-3	1:109407135	rs11102571	0.112	0.103	86.27 (21.81)	0.985	1.34 × 10^−09^	3.029 × 10^−06^
ST1A1	16:28595989	rs138534121	0.064	NA**	83.89 (16.79)	NA**	2.51 × 10^−13^	NA**
STAMBP^*^	1:53206258	1:53206258^§^	0.026	NA**	29.16 (20.59)	NA**	3.32 × 10^−09^	NA**
TGFB1	19:41847860	rs1800472	0.040	0.041	88.09 (14.06)	1	1.35 × 10^−12^	1.55 × 10^−08^
TNFB	6:31540757	rs2229092***	0.027	0.027	76.95 (17.90)	1	2.70 × 10^−29^	6.04 × 10^−21^***
TNFSF14	19:6665020	rs344560	0.065	0.066	91.63 (11.59)	1	3.72 × 10^−17^	7.18 × 10^−01^
uPA	19:44202855	rs346058	0.046	0.043	87.38 (14.18)	0.955	7.12 × 10^−09^	2.89 × 10^−03^

MAF is shown for both the WGS and imputed data set as well as genotype quality for the WGS data and imputation quality for the imputed data. The lowest p-value is shown from the WGS study and the p-value from combined analyses*** of the INF biomarkers published by Enroth *et al*.^28^. The genome-wide significant threshold used in the WGS study was 1.62 × 10^−8^ and in the previous study using genotyped/imputed data, a more stringent threshold of 4.79 × 10^−9^ were used, adjusting for the total number of markers analyzed rather than the total number of independent tests performed.

^§^Does not have an rs-id.

^†^In hg19 coordinates.

*Likely to be false positive findings (STAMBP) due to low genotype quality and CXCL9/ CXCL11 is discussed in the Supplementary (including Supplementary Figs S21–S25)

^**^Did not pass imputation QC or were not present in the reference panel used for the imputations.

^***^Two associations were not reported in our previous study due to the two-stage design (discovery and replication) even though the p-value was significant in the combined analyses.

We also compared our association signals for colocalization with data from the GWAS catalog. A total of eight biomarkers (CCL19, CCL4, CD40, CD6, CXCL5, IL-12B, IL-18R1, TNFSF14) had top variants previously associated with an inflammatory disease, or correlated (R^2^ > 0.8) with variants previously associated with an inflammatory disease, suggesting that variation in biomarker levels might mediate the disease association, although, we did not determine the direction of causality. For example, the minor allele of rs10190555 was found to be associated with higher levels of interleukin 18 receptor 1 (IL-18R1). The minor alleles of two SNPs in LD (R^2^ = 0.95) with rs10190555 (rs917997[T] and rs6708413[G]) are also associated with a higher risk of inflammatory bowel disease (IBD) and Crohn’s disease respectively. IL-18R1 is a cytokine receptor that binds interleukin 18 (IL-18) and is essential for IL-18 mediated signal transduction. IL18R1 has previously been found to have colocalized disease and eQTL association patterns in CD4 and CD8 cells for both ulcerative colitis and Crohn’s disease^34^. In that study, reduced transcript levels of *IL18R1* in CD4 and CD8 cells was associated with increased risk for IBD, and the SNP most strongly associated with expression was rs11123923. However, rs11123923 is only in weak LD with the top variant (rs10190555) from our study (R^2^ = 0.20). In our study, the association to IL-18R1 spans over a large region with strong LD. When adjusting for the top variant, a secondary top variant (rs12999517) reached genome-wide significance. This variant is intronic in *IL1RL1*, which encodes the interleukin-1 receptor-like 1 (IL1RL1, alternatively ST2) protein. In a previous functional study, the C allele in rs6543115, which is located in a distal *IL1RL1* promoter, was shown to confer susceptibility to ulcerative colitis as well as increase expression of the soluble ST2 isoform^35^. However, the top conditional variant (rs12999517) from our study is not in LD with the previously found variant, and is not in strong LD with any other variant previously associated with ulcerative colitis.

When adjusting for the effects of the top SNP (rs344560) associated with TNFSF14, the minor allele of rs2291668 was found to be significantly associated with increased TNFSF14 levels in our study. Interestingly, the secondary variant rs2291668 is found to explain significantly more of the variability in TNFSF14 levels (9.1%, as compared to 4.4% explained by rs344560 [likelihood-ratio test, p < 0.001, χ^2^ = 26.95, 1 d.f.]). The top SNP (rs344560) in the primary signal is a missense variant located in the gene *TNFSF14*, which encodes the biomarker. This variant was found to be associated with lower TNFSF14 levels. The missense variant is neither reported in the GWAS catalog, nor is it in LD with any previously reported variants. However, the conditional SNP, rs2291668, is correlated with rs1077667 that has previously been associated with multiple sclerosis. A possible explanation is that the missense variant affects the antibody affinity for the biomarker which in turn will give lower measured protein levels. These lower levels might give a strong association signal and it is not until the top missense variant is adjusted for, that the true regulatory variant is detected.

A subset of our top SNVs overlapped with eQTLs in blood. For example, the correlated variants rs352045 and rs425535 (top SNVs for CXCL5 in the INF and ONC_CVD respectively) are in high LD with an eQTL for *CXCL5* RNA levels in blood. Two variants, rs425535: a nonsynonymous exonic variant that lies within a splicing enhancer site, and rs352046: a promoter variant that is located within a transcription factor binding site for myeloid zinc finger proteins, have also previously been associated with *CXCL5* mRNA expression^38^ and CXCL5 protein levels in blood^39^. Both variants have been shown to be in almost complete LD (R^2^ = 0.94) to complete LD (R^2^ = 1) in both U.S and European populations^36,39^ and are in complete LD in our cohort. The minor allele for rs425535 was previously shown to be associated with significantly higher *CXCL5* plasma concentrations and the minor allele for rs352046 with higher *CXCL5* expression levels, which agrees with our results where the minor alleles of rs352045 and rs425535 were associated with higher CXCL5 levels (Supplementary Table S6). In our study, rs352046 was not identified as the top variant but instead rs352045 which is in almost complete LD with rs352046 (R^2^ = 0.97). Our top SNV is located only 137 bp from rs352046 and both are found within transcription factor binding sites. Neither the association with rs352045 nor rs352046 are reported in the GWAS catalog. However, both rs352045 and rs425535 are in high LD (R^2^ = 0.94) with a variant, rs2457996, that has previously been associated with ulcerative colitis. Ulcerative colitis is a sub type of IBD and *CXCL5* have previously been shown to play a role in IBDs, such as ulcerative colitis and Crohn’s disease. In a study by Z’Graggen *et al*.^40^, a preferential expression of *CXCL5* mRNA in the epithelium of the intestinal tissue from patients with IBD was observed. They also found a strong expression of *CXCL5* at protein level. *CXCL5*, which encodes an epithelial cell-derived neutrophil activating peptide (also called *ENA-78*), has previously been suggested as a possible candidate gene for inflammatory diseases^36,37^ Since the previously mentioned variants, rs425535 and rs352046, have been shown to be associated with higher *CXCL5* plasma concentrations and higher *CXCL5* expression levels respectively, this further indicates that these variants might play a role in the pathogenesis of IBD.

Despite the many results, our study has some limitations with one being the limited sample size. Standard GWAS of complex traits commonly includes hundreds of thousands of samples. However, by analyzing quantitative phenotypes that are less complex, such as biomarkers, we can gain power and the sample size can be dramatically reduced. Despite this, for some biomarkers a sample size of <1000 individuals are not enough to make a robust assessment, and further studies in larger cohorts, or meta-analyzes needs to be performed. Another limitation is the lack of reproducibility, given the nature of the study. It is a small kinship-structured cohort, which makes the results not generalizable to more mixed population or a population of another ancestry. While this population structure increases power to detect rare variants that might be more common in an isolated population, it also makes it harder to reproduce in another cohort. As of now, there are also only a limited number of cohorts that have been measuring the levels of the same inflammatory biomarkers and that have WGS data available. Even within our own data, we fail to replicate some results, both in the technical replicates as well as in the novel associations with regards to the results from genotyped/imputed data. The limitations mentioned above are most likely also dependent on differences in biomarker quantifications between the biomarker panels. As with imputation, variant calling can be more or less precise. Only a genotype probability is given, with additional quality measures. Caution should be taken, until the associations have been validated. This applies especially to the associations containing only a few variants, or variants not yet given an official identifier. Validation has to be performed in a similar cohort, in order to obtain higher confidence and better understanding of the results. This limitation was especially apparent in our validation in the Finnish population, with a possible reason for lack of replication being the discrepancy in population size (around 950 in NSPHS compared to up to 8,293 Finns). Other possible explanations for the lack of replication are the different LD structures, the different techniques used for protein quantification, and that one cohort being based on WGS and the other on genotyped and imputed data.

In summary, we have performed GWAS in a family-based cohort with WGS data in relation to inflammatory biomarkers. By analyzing only sequencing data, we seek to further extend our knowledge on the genetic contribution to these important biomarkers. The cost-efficient solution of sequencing a few individuals and creating a reference panel to be able to do dense genotyping is becoming a well-established method in genetic studies. The use of low-depth sequencing as a way of increasing power is also more common today. By using high coverage WGS data, we do see an increase in both power and precision despite our limited sample size, an increase which indeed appears promising. We compared our results to earlier studies using genotyped/imputed data as well as to previously published GWAS. This study found several new loci associated with inflammatory biomarkers and nearly 50% of the associations were only detected in the present study. The associations were also stronger, with lower p-values, compared to those identified with genotyped/imputed data, suggesting that genotypes are in general more accurately determined using WGS compared to imputed data. Our results demonstrate the need of deep coverage WGS data with deeper coverage to be able to fully understand the genetic structure of common diseases and complex traits.

## Methods

### Study cohort

The NSPHS was initiated in 2006 to provide a health survey of the population in the parish of Karesuando county of Norrbotten, Sweden, to study the medical consequences of lifestyle and genetics. Additional participants were recruited in a second phase from the neighboring village Soppero, in 2009. These parishes have about 2,000 inhabitants of which a total of 1,069 participated in the study, whereof 719 individuals participated from Karesuando (2006) while another 350 individuals participated from Soppero (2009). For each participant in the NSPHS, blood samples were taken and serum and plasma were separated and immediately frozen and stored at −70 °C^23^.

### Ethical considerations

The NSPHS was approved by the local ethics committee at the University of Uppsala (Regionala Etikprövningsnämnden, Uppsala, 2005:325, and extension of the project was approved 2016-03-09) in compliance with the declaration of Helsinki^41^. Informed consent to the study was given by all participants, including the examination of environmental and genetic cause of disease. If a person was not of age (<18 years), a legal guardian signed additionally. The procedure that was used to obtain informed consent and the respective informed consent form has recently been discussed in light of present ethical guidelines^42^.

### Genetic data

A total of 1,041 samples were successfully sequenced using Illumina short read technology (X-ten) to 30x coverage per individual. The library preparation, sequencing, and variant calling were performed as previously described^10^. Briefly, WGS data were aligned to the GR37 using bwa-mem v0.7.12^43^. The raw alignments were then processed according to GATK best practice^44^ using GATK v3.3. Variants were called by the GATK HaplotyeCaller 3.3 followed by variant quality score recalibration (VQSR). Sample quality control (QC) was then performed to remove genetic outliers and identify potentially contaminated samples and individuals with sex discordance errors. After QC, 1,021 unique samples with WGS data remained. Before analysis, the VCF-files were converted to PLINK-format with the PLINK software, version 1.90b4.9^45^. Only autosomes and biallelic single nucleotide variants (SNVs) were included in the analysis. If a position had more than two alleles, PLINK keeps the two most common variants and sets the third one to a missing genotype. SNVs within a deletion were also excluded (spanning deletion/overlapping deletion, denoted *). In the same process, the variants without an rs-id were renamed to chr:position. MAF and deviation from HWE information were assessed with the --freq and --hardy commands in PLINK. The GWA analyses were performed using the GenABEL package in R^46,47^. To make the files compatible with GenABEL, they were first transposed with the --recode transpose command in PLINK, and then imported into GenABEL. Variants were annotated with ANNOVAR v 2017.07.16^48^ using the refGene database.

### Biomarker data

Out of 1,021 individuals with WGS data, up to 1,011 individuals also have measured levels of any inflammatory biomarkers using the Proximity Extension Assay (PEA) technology provided by Olink (https://www.olink.com/products/inflammation/). Inflammatory biomarkers have been measured at three different timepoints within the cohort. At the first two timepoints the panels Oncology I (ONC I)^23^ and Cardiovascular I (CVD I)^25^ were measured. These include 31 inflammatory biomarkers (ONC_CVD). A total of 1,005^25^ samples were measured at the first two timepoints. At the third timepoint^28^, the panel INF I was used to measure biomarker levels (INF). Here, 92 biomarkers were measured in 903 individuals^28^. Of the inflammatory biomarkers from the ONC_CVD dataset, 30 had overlapping measurements in the INF panel, and thus served as technical replicates. The quality control of the biomarkers has been described previously^23,25,28^. After quality control, up to 957 individuals had available biomarker data in ONC_CVD and up to 892 from INF. Biomarkers with measurements in less than 400 individuals were excluded from downstream analyses.

### GWAS

The GenABEL package was used to perform GWAS adjusting for relatedness among individuals. GenABEL utilizes a genetic kinship matrix which was estimated with the ibs function. The kinship matrix was estimated based on the SNPs listed for the HumanHap300v2_A chip. This chip contains >300,000 SNPs that are selected to be tagSNPs, i.e. that are not in high LD with each other. This was done to remove non-informative variants in the construction of the kinship matrix. The phenotypic measurements and possible covariates, together with the kinship matrix, are passed to the polygenic function of GenABEL. The residuals from the polygenic model and the inverse covariance-matrix are then passed on to the mmscore, a linear mixed-effects model, which was used to perform the association analysis. All biomarker levels were rank-transformed to standard normal distributions with the rntransform function in GenABEL prior to the GWAS. All biomarker values were adjusted for sex, age and batch effect prior to, or in the GWA analyses. A Bonferroni adjusted p-value threshold was applied to account for the number of independent tests. To calculate the number of independent SNVs in the analysis, LD-pruning was performed in PLINK, using the --indep-pairwise function, with a window size of 10 Mb and variant jump count of 1. This resulted in a p-value cut-off of *p*_threshold_ = 0.05 / 3,078,707 independent SNVs = 1.62 × 10^−8^. A MAF threshold of 0.15% in the primary analyses and HWE cut-off of 5 × 10^−8^ was used. The MAF threshold was determined from simulation by assuming that the individuals with the most extreme biomarker levels were the only carriers of the minor allele at a given position. The minimum p-values were estimated depending on the number of individuals carrying one copy of the minor allele, as well as on sample size (Supplementary Fig. S1). Given a sample size of 700–1,000 individuals, which corresponds to the sample size for the biomarkers analyzed, a minimum of four individuals with one copy of the minor allele is needed to reach the genome wide significance threshold of our study. Since three copies in 1000 individuals corresponds to a frequency of 0.15%, we therefore used 0.15% as the MAF threshold in the primary GWA analyses of our study (more than three copies per 1000 individuals).

QQ-plots and Manhattan plots were produced with the qqman package in R^49^. Regional association plots were constructed using Locuszoom^50^. The 1000 G Nov 2014 EUR population was used for the coloration based on LD. If one biomarker had different top SNVs in the technical replicates, the LD coefficient (R^2^) was calculated using PLINK within the study population itself (NSPHS). To assess the size of the associated regions, an SNV clumping was performed in PLINK. A clump kb radius of 15 Mb (--clump-kb), a p-value threshold of 1 × 10^−8^ (--clump-p1) and an R^2^ cut-off set to default (--clump-r2 0.1) was used. This function clumps SNVs together based on empirical estimates of LD. The range (in bp) of the clumps was then calculated for each biomarker and used to define biomarker-based loci. We also performed conditional analysis, where the top SNV, for each marker with a significant hit, was used as covariate in the mmscore function to see whether there was more than one independent association for each biomarker. These clump-defined loci were then used to calculate a biomarker-based significance threshold for results in the conditional analyses. SNVs with a conditional p-value below 0.05/number of SNVs tested in the predefined locus, were considered as an independent association. If the biomarker had a significant secondary (conditional) signal, a third analysis was performed, adjusting for both the primary and secondary signal. No MAF cutoff was used in the conditional analyses.

### Narrow-sense heritability estimates

Narrow-sense heritability (h^2^) was estimated using the polygenic model in GenABEL. First, the heritability for the biomarker measurements was estimated only adjusting for age, sex, batch effects and kinship. Then, as an estimation of SNV heritability, the top variant was used as a covariate. The difference in heritability estimated between the models gives the variance explained by the top variant. If secondary and tertiary signals were present, they were added as additional covariates and SNV heritability was calculated for each separately. To test significance of heritability, the reported function minimum (twice the negative maximum log-likelihood) is compared to the reported function minimum in a polygenic model with a fixed heritability estimate set to zero. The difference gives a test approximately distributed as chi-squared with 1 degree of freedom.

### Colocalization with published GWAS data and comparison with previous biomarker studies

If the top SNVs of two different GWASes are in LD (R^2^ > 0.8), the phenotypes are considered to be colocalized. In the study cohort, the LD pattern was calculated between each top SNVs and all SNVs within 2 Mb, using PLINK. All variants that were in LD (R^2^ > 0.8) with a top SNV were extracted. These were used as query to test for colocalization with data from the GWAS catalog (The NHGRI-EBI Catalog of published genome-wide association studies, https://www.ebi.ac.uk/gwas/home) to find out whether the variants have already been published in earlier association studies of inflammatory biomarkers. Entries with p-values up to 1 × 10^−6^ in the GWAS catalog (version 1.0.2 – downloaded 2018-10-29) were included in the comparison. All entries for the top variants and variants in LD found in the catalog was extracted together with their metadata.

Our GWAS results were also tested for colocalization with the expression quantitative trait locus (eQTL) dataset from Westra *et al*.^51^. This dataset consists of both *cis-*eQTL and *trans*-eQTLs and is based on an eQTL meta-analysis in non-transformed peripheral blood samples. The names of the genes encoding the biomarkers were matched both in the *cis* and *trans* dataset to see if the top SNVs in this study had been reported as an eQTL. If not, LD was calculated between the top SNVs of our study and the most significant eQTL variant, using PLINK, also here within the study cohort (NSPHS).

Further replication was made using another population from Northern Europe. Circulation cytokines have been measured in Finnish populations by Ahola-Olli *et al*.^29^. We sought to replicate the results from the 19 inflammatory biomarkers (i.e. cytokines) that were present in both studies: bNGF, CCL11, CCL3, CCL4 (MIP1b), CXCL1 (GROa), CXCL10 (IP10), CXCL9 (MIG), HGF, IL-4, IL-5, IL-5, IL-7, IL-8, IL-10. IL-13, IL-18, MCP-1, MCP-3, SCF, TNFB and TRAIL. However, IL-4, IL-5 and IL-13 did not pass QC in our study, and thus, only 16 biomarkers could be compared. Only top SNVs were used in the replication.

Top variants were compared to the results from previous studies with genotyped/imputed data in the same cohort^25,28^. In the first study, the ONC_CVD biomarkers were analyzed^25^ in the whole cohort whereas in the second study^28^, the INF biomarkers were analyzed using a two-stage design by splitting the cohort into a discovery and replication cohort (genotyped using different arrays and imputed independently) followed by combined analyses for the SNVs that replicated. Even if the two previous studies were based on the same genotyping and imputation data, the quality control of the imputed genotypes where slightly different with one additional requirements of a genotype probability score of Info >0.9 in 95% of the individuals for the analyses of the INF biomarkers whereas the criteria of imputation quality >0.3 and HWE p-value >0.05/the number of SNPs within the sub cohorts based on different genotyping arrays as well as after combining the two sub cohorts. More information about genotyping, imputation and quality control of imputed variants is included in the previous articles^25,28^. In this comparison, the additional filters were also applied to the imputed data similar to the WGS data, i.e. only biallelic autosomal hits were compared. If an association was previously found with an indel or a variant in a spanning deletion, the biallelic variant with the lowest p-value after the indel or spanning deletion was used for comparison. For a subset of the non-overlapping hits, we also compared the WGS genotypes with the imputed dosage values form the previous studies.

## Supplementary information

Supplementary Information

## Data Availability

Summary statistics from the GWAS will be uploaded to the GWAS catalog.
