# Supplementary material for: Improved power and precision with whole genome sequencing data in genome-wide association studies of inflammatory biomarkers
Source: Sci Rep. 2019 Nov 14;9:16844. doi: 10.1038/s41598-019-53111-7 (PMC6856527; doi:10.1038/s41598-019-53111-7)
Supplement: Supplementary file 1 — Supplementary Information [file 41598_2019_53111_MOESM1_ESM.pdf]

Improved power and precision with whole genome sequencing data in genome-wide association studies of inflammatory biomarkers

Julia Höglund[1*], Nima Rafati[1], Mathias Rask-Andersen, PhD[1], Stefan Enroth, PhD[1], Torgny Karlsson, PhD[1], Weronica E. Ek, PhD[1], Åsa Johansson, PhD[1]

[1] Department of Immunology, Genetics and Pathology, Science for Life Laboratory, Uppsala University, Uppsala, Sweden

Supplementary material

Tables

Supplementary Table S1. Sequence depth and quality for top GWAS hits. All variants are from WGS data sequence at a mean coverage of 30x. VQSR tranche‡, genotype quality score and sequence depth are presented for each top variant as well as whether they are in a low complexity region (as defined in ref [3])

Biomarker	chr:position†	SNV	VSQRTrancheSNP‡	GQ‖ (sd)	DP¶ (sd)	Low complexity region
**ADA**	20:43255220	rs11555566	PASS	86.50 (14.78)	31.05 (5.69)	no
**CASP-8**	2:202178477	rs116010659	PASS	90.00 (12.94)	31.78 (5.96)	no
**CCL11**	3:42906116	rs2228467	PASS	87.10 (14.11)	31.06 (5.67)	no
**CCL19**	6:32556454	rs149941420	99.00to99.90	90.92 (13.34)	31.26 (7.82)	yes
**CCL20**	11:102742761	rs17368659	PASS	96.12 (7.33)	36.06 (5.39)	no
**CCL23**	17:34326215	rs712048	PASS	95.65 (13.34)	31.26 (7.82)	no
**CCL25**	19:8121360	rs2032887	PASS	92.07 (12.10)	32.41 (6.96)	no
**CCL4**	3:46457412	rs113010081	PASS	92.44 (11.21)	32.94 (5.82)	no
**CCL4**	17:34490448	rs4141329	99.00 to 99.90	94.92 (10.96)	40.53 (15.50)	yes
**CD244**	1:160802681	rs71517284	PASS	91.14 (13.84)	29.50 (6.50)	yes
**CD40**	20:44742064	rs1569723	PASS	95.30 (8.69)	35.04 (7.19)	no
**CD40**	20:44749251	rs4239702	PASS	97.09 (6.08)	38.00 (7.06)	no
**CD6**	11:60776209	rs11230563	PASS	90.08 (13.40)	31.98 (7.27)	no
**CDCP1**	3:45176513	rs78521038	PASS	94.40 (9.59)	34.58 (5.99)	no
**CST5**	20:23859017	rs4239743	99.00 to 99.90	94.61 (9.83)	33.35 (6.47)	yes
**CX3CL1**	16:57374418	rs9921681	PASS	82.93 (18.48)	26.98 (5.96)	no
**CXCL1**	4:74734668	rs3117604	PASS	91.77 (12.80)	32.77 (6.02)	no
**CXCL10**	4:76943947	rs11548618	PASS	92.21 (10.60)	33.55 (4.64)	no
**CXCL11**	4:76943947	rs11548618	PASS	92.21 (10.60)	33.55 (4.64)	no
**CXCL5**	4:74864687	rs352045	PASS	89.77 (13.54)	32.09 (7.90)	no
**CXCL5**	4:74863997	rs425535	PASS	92.63 (10.84)	34.10 (7.67)	no
**CXCL5**	10:65101207	rs10740118	PASS	96.52 (7.24)	36.52 (6.80)	no
**CXCL5**	10:65016174	rs7088799	PASS	97.22 (6.00)	37.53 (6.58)	no
**CXCL6**	4:74700432	rs111903579	99.00 to 99.90	85.04 (18.31)	24.95 (6.01)	yes
**CXCL9**	4:76943947	rs11548618	PASS	92.21 (10.60)	33.55 (4.64)	no
**FGF-5**	4:81184341	rs16998073	PASS	93.58 (11.26)	34.08 (6.47)	no
**Flt3L**	13:28761592	rs145096717	PASS	94.67 (8.47)	34.60 (4.02)	no
**Flt3L**	13:28768589	rs111595024	PASS	50.08 (21.35)	31.56 (8.06)	yes
**IL-10RB**	21:34660980	rs8178528	PASS	95.79 (8.07)	34.32 (6.48)	no
**IL-12B**	5:158767333	rs10043720	PASS	91.15 (12.75)	31.45 (7.14)	no
**IL-15RA**	10:5997820	rs3136630	PASS	91.43 (12.75)	31.05 (6.03)	no
**IL-18R1**	2:102994056	rs10190555	PASS	94.92 (9.44)	36.58 (8.67)	no
**MCP-1**	3:46412559	rs1800024	PASS	93.57 (9.38)	34.12 (4.75)	no
**MCP-2**	17:32647831	rs1133763	PASS	91.07 (12.04)	32.62 (5.42)	no
**MCP-3**	1:109407135	rs11102571	PASS	86.27 (21.81)	34.22 (6.01)	no
**MCP-4**	1:159175354	rs12075	PASS	94.55 (10.13)	33.67 (7.23)	no
**MMP-1**	11:102697731	rs471994	PASS	94.33 (10.16)	33.87 (6.60)	no
**MMP-10**	11:102643718	rs17359286	PASS	93.56 (9.98)	34.27 (5.14)	no
**SCF**	20:44551855	rs6073958	PASS	96.37 (7.18)	37.71 (5.89)	no
**ST1A1**	16:28595989	rs138534121	PASS	83.90 (16.79)	30.28 (6.49)	no
**STAMPB**	1:53206258	1:53206258§	99.00 to 99.90	29.16 (20.59)	30.23 (7.71)	yes

**TGFB1**	19:41847860	rs1800472	PASS	88.09 (14.06)	31.58 (5.95)	no
**TNFB**	6:31540757	rs2229092	PASS	76.95 (17.90)	28.01 (6.11)	no
**TNFSF14**	19:6665020	rs344560	PASS	91.63 (11.59)	33.60 (7.58)	no
**TRAIL**	18:29769910	rs144242131	PASS	90.74 (12.23)	33.48 (5.15)	no
**uPA**	19:44202855	rs346058	PASS	87.38 (14.18)	32.98 (6.05)	yes
**VEGF-A**	6:43925607	rs6921438	PASS	95.10 (10.09)	35.44 (6.63)	no

§ Does not have an rs-id,
† In hg19 coordinates.
‡ Variant Quality Score Recalibration Tranche
‖ Genotype quality
¶ Sequence depth

Supplementary Table S2. Results for biomarkers with significant results. The most significant variant and its p-value is presented for each panel. If the top variant is the same for both panels, the variant and its p-value is stated twice. For the biomarkers where a technical replicate is available (i.e when the biomarkers is present on both the ONC_CVD panel and the INF panel), the linkage disequilibrium between the top variants is shown.

**Biomarker**	**Most significant SNV in ONC_CVD**	**P in ONC_CVD**	**P in INF**	**Most significant SNV in INF**	**P in INF**	**P in ONC_CVD**	**R^2†**
**ADA**	-	-	-	rs11555566	4.91E-18	-	-
**CASP-8**	rs116010659	3.63E-09	6.94E-09	rs116010659	6.94E-09	3.63E-09	1
**CCL11**	-	-	-	rs2228467	2.19E-09	-	-
**CCL19**	-	-	-	rs149941420	4.27E-18	-	-
**CCL20***	rs17368659	1.40E-09	8.17E-02	20:57225811§	7.64E-08	1.46E-03	0.00149
**CCL23**	-	-	-	rs712048	1.28E-12	-	-
**CCL25**	-	-	-	rs2032887	1.09E-37	-	-
**CCL4**	rs113010081	1.50E-35	4.19E-38	rs113010081	4.19E-38	1.50E-35	1
**CCL4**	rs4141329	1.55E-14	1.07E-12	rs4141329	1.07E-12	1.55E-14	1
**CD244**	-	-	-	rs71517284	1.16E-13	-	-
**CD40**	rs4239702	1.01E-49	2.52E-41	rs1569723	5.24E-43	1.15E-49	0.913
**CD6**	-	-	-	rs11230563	5.23E-31	-	-
**CDCP1**	-	-	-	rs78521038	2.62E-12	-	-
**CST-5**	-	-	-	rs4239743	8.61E-21	-	-
**CX3CL1***	rs9921681	3.37E-10	7.31E-07	rs598366	8.45E-08	2.50E-06	0.486
**CXCL1**	rs3117604	3.87E-09	2.46E-19	rs3117604	2.46E-19	3.87E-09	1
**CXCL10**	rs11548618	5.64E-42	5.07E-47	rs11548618	5.07E-47	5.64E-42	1
**CXCL11***	rs11548618	3.44E-13	6.82E-01	rs10932734	1.01E-07	2.77E-04	0.000675
**CXCL5**	rs425535	1.09E-34	6.09E-27	rs352045	6.09E-27	1.09E-34	1
**CXCL5**	rs7088799	7.36E-16	2.93E-13	rs10740118	2.93E-13	7.36E-16	1
**CXCL6**	rs111903579	6.71E-58	1.52E-48	rs111903579	1.52E-48	6.71E-58	1
**CXCL9***	rs11548618	6.19E-10	7.78E-01	rs10462509	6.68E-08	1.12E-02	0.00178
**FGF-5**	-	-	-	rs16998073	1.50E-11	-	-
**Flt3L**	rs111595024	1.01E-16	3.49E-16	rs145096717	2.09E-16	1.26E-16	0.967
**IL-10RB**	-	-	-	rs8178528	5.46E-35	-	-
**IL-12B**	-	-	-	rs10043720	7.99E-31	-	-
**IL-15RA**	-	-	-	rs3136630	2.64E-19	-	-
**IL-18R1**	-	-	-	rs10190555	2.37E-72	-	-
**MCP-1***	rs1800024	1.26E-09	1.07E-07	rs10510751	8.73E-08	1.76E-09	0.979
**MCP-2**	-	-	-	rs1133763	2.69E-53	-	-
**MCP-3**	-	-	-	rs11102571	1.34E-09	-	-
**MCP-4**	-	-	-	rs12075	1.25E-45	-	-
**MMP-1**	rs471994	5.02E-19	4.87E-15	rs471994	4.87E-15	5.02E-19	1
**MMP-10***	rs17359286	1.17E-08	6.71E-08	rs486055	1.65E-08	1.02E-07	0.583
**SCF***	rs6073958	1.20E-09	5.47E-06	rs2868346	1.40E-07	4.02E-08	0.570
**ST1A1**	-	-	-	rs138534121	2.51E-13	-	-
**STAMBP**	-	-	-	1:53206258§	3.32E-09	-	-
**TGFB1**	rs1800472	1.35E-12	3.18E-11	rs1800472	3.18E-11	1.35E-12	1
**TNFB**	-	-	-	rs2229092	2.70E-29	-	-
**TNFSF14**	rs11793489	4.98E-07	1.02E-02	rs344560	3.72E-17	3.52E-03	0.00292
**TRAIL***	rs144242131	1.02E-12	-	-	-	-	-
**uPA**	-	-	-	rs346058	7.12E-09	-	-
**VEGF-A**	rs6921438	1.64E-12	8.29E-40	rs6921438	8.29E-40	1.64E-12	1

§Does not have an rs-id.
*Only had significant hits in the technical replicate (ONC_CVD)
†LD between the most significant SNVs from the two different biomarker panel

Supplementary Table S3. Results from biomarkers with significant conditional results. The most significant variant and its p-value is presented for each panel. If the top variant is the same for both panels, the p-value is stated twice. When a technical replicate is available, linkage disequilibrium (R^2) between the top variant and the technical replicate is shown.

Marker	Most significant SNV in ONC_CVD	P in ONC_CVD	P in INF	Most significant SNV in INF	P in INF	P in ONC_CVD	$R^{2\dagger}$
**CCL23**	-	-	-	rs72831705	9.52E-11	-	-
**CCL4**	3:51599851[§]	3.53E-07	5.28E-07	3:51599851[§]	5.28E-07	3.53E-07	1
**CCL4**	rs188700215	1.01E-07	8.01E-01	rs186218230	2.82E-05	3.29E-01	5.47E-05
**CD40**	rs6063068	7.41E-08	2.28E-06	rs193101079	3.31E-06	2.02E-07	0.4998
**CST-5**	-	-	-	rs6138152	3.87E-07	-	-
**CXCL1**	rs10938101	7.53E-07	4.43E-02	rs9991912	1.80E-04	1.62E-02	0.0405
**CXCL6**	rs181216093	5.27E-09	8.05E-09	4:73906728[§]	6.47E-09	1.02E-07	0.841
**IL-15RA**	-	-	-	rs144173272	1.49E-11	-	-
**IL-18R1**	-	-	-	rs12999517	4.89E-19	-	-
**MCP-2**	-	-	-	rs74832623	6.47E-32	-	-
**MMP-1**	rs470358	9.38E-09	8.84E-06	rs514921	6.28E-08	1.47E-08	0.1425
**SCF**	rs6104417	2.66E-06	-	no significant primary signal	-	-	-
**ST1A1**	-	-	-	rs4149383	5.38E-07	-	-
**TNFSF14**	no significant primary signal	-	-	rs2291668	4.71E-07	-	
**TRAIL[*]**	18:21026109[§]	1.01E-07	-	-	-	-	-

[§]Does not have an rs-id
[*]Only had significant hits in the technical replicate (ONC_CVD)
[†]LD between the most significant SNVs from the two different biomarker panels.

Supplementary Table S4. Sequence depth and quality for the top GWAS hits in the conditional analyses. All variants are from the WGS data sequence at a mean coverage of 30x. VQSR tranche[‡], genotype quality score and sequence depth are presented for each top variant as well as whether they are in a low complexity region (as defined in ref [3])

Biomarker	chr:position[†]	SNV	Conditional signal	VSQRTrancheSNP[‡]	GQ[‖] (sd)	DP[¶] (sd)	Low complexity region
**CCL23**	17:34321277	rs72831705	secondary	PASS	95.66 (7.92)	34.88 (3.67)	no
**CCL23**	17:34361300	rs854671	tertiary	PASS	66.08 (22.73)	29.67 (7.35)	no
**CCL4**	3:51599851	3:51599851[§]*	secondary	PASS	90.29 (12.52)	32.90 (5.57)	no
**CCL4**	17:30092085	rs188700215*	secondary	PASS	95.69 (8.53)	35.13 (6.55)	no
**CCL4**	17:34522125	rs201079256*	tertiary	99.00 to 99.90	94.97 (8.97)	34.38 (5.93)	yes
**CD40**	20:45717496	rs6063068*	secondary	PASS	90.27 (12.67)	31.77 (5.57)	no
**CD40**	20:44730041	rs182282247*	tertiary	PASS	89.43 (13.37)	32.14 (5.20)	no
**CST-5**	20:23850130	rs6138152	secondary	PASS	90.20 (11.87)	32.65 (4.92)	no
**CST-5**	20:29478349	rs75823487	tertiary	99.00 to 99.90	87.13 (13.89)	31.80 (5.80)	yes
**CXCL1**	4:74688772	rs10938101*	secondary	PASS	86.89 (13.47)	31.74 (5.09)	no
**CXCL6**	4:74661204	rs181216093*	secondary	PASS	52.91 (29.89)	18.20 (11.21)	no
**IL-15RA**	10:6008255	rs144173272	secondary	PASS	88.76 (15.44)	29.77 (6.83)	no
**IL-15RA**	10:5700416	rs35095871	tertiary	PASS	90.49 (12.79)	33.27 (6.40)	no
**IL-18R1**	2:102959260	rs12999517	secondary	PASS	87.85 (15.46)	34.58 (11.71)	no
**MCP-2**	17:32535173	rs74832623	secondary	PASS	97.30 (5.76)	38.33 (6.98)	no
**MCP-2**	17:32533423	rs12601658	tertiary	PASS	85.87 (14.24)	31.04 (5.28)	no
**MMP-1**	11:102668702	rs470358*	secondary	PASS	93.73 (10.38)	32.19 (6.67)	no
**SCF**	20:44632542	rs6104417*	secondary	PASS	94.36 (9.23)	34.61 (5.78)	no
**ST1A1**	16:28620320	rs4149383	secondary	99.00 to 99.90	93.70 (10.11)	33.89 (5.41)	yes
**TNFB**	6:31540429	rs746868	secondary	PASS	78.17 (22.34)	22.72 (6.34)	no
**TNFB**	6:27190519	6:27190519§	tertiary	PASS	94.40 (9.19)	36.18 (5.17)	no
**TNFSF14**	19:6669934	rs2291668	secondary	PASS	75.09 (25.37)	29.59 (8.99)	no
**TRAIL**	18:21026109	18:21026109[§]*	secondary	PASS	95.96 (7.87)	34.72 (6.27)	no

[§]Does not have an rs-id.
[†]In hg19 coordinates.
‡ Variant Quality Score Recalibration Tranche
‖ Genotype quality
¶ Sequence depth
*Variant is from ONC_CVD. Either the p-value was lower, or no significant association was found in INF

Supplementary Table S5. Narrow sense heritability (h2) for each biomarker. h2 is the overall heritability. h2 (~top) is the heritability conditioned of the top variant, h2 (~top+second) on the top variant and top from the conditional analysis when it has been performed. h2 (~top+second+tertiary) shows the heritability conditioned on the top variant, the top from the first conditional analysis and the second, when the biomarkers had significant tertiary peaks. The biomarkers with a non-significant heritability estimate are marked with "n.s".

Biomarker	h2	h2 (~top)	h2 (~top+second)	h2 (~top+second+tertiary)
**ADA**	0.308	0.250		
**AXIN1**	0.161			
**Beta-NGF**	0.382			
**CASP-8**	0.310*	0.272*		
**CCL11**	0.455	0.435		
**CCL19**	0.216	0.141		
**CCL20**	0.356*	0.330*		
**CCL23**	0.416	0.407	0.308	0.230
**CCL25**	0.470	0.265		
**CCL28**	0.103 n.s.			
**CCL3**	0.265			
**CCL4 (chr17)**	0.706*	0.674*	0.674*	0.615*
**CCL4 (chr3)**	0.707	0.561	0.571	
**CD244**	0.464	0.395		
**CD40**	0.462*	0.250*	0.241*	0.217*
**CD5**	0.521			
**CD6**	0.472	0.359		
**CDCP1**	0.380	0.282		
**CSF-1**	0.186			
**CST5**	0.522	0.479	0.403	0.443
**CX3CL1**	0.283*	0.248*		
**CXCL1**	0.332	0.233		
**CXCL1 CXCL10**	0.482*	0.443*	0.393*	
	0.410*	0.303*		
**CXCL11**	0.341*	0.293*		
**CXCL5 (chr10)**	0.605*	0.566*		
**CXCL5 (chr4)**	0.605*	0.587*		
**CXCL6**	0.556	0.310	0.299	
**CXCL9**	0.312*	0.249*		
**DNER**	0.310			
**EN-RAGE**	1.087e-08			
**FGF-19**	0.169			
**FGF-21**	0.337			
**FGF-23**	0.260			
**FGF-5**	0.133 n.s.	0.077 n.s.		
**Flt3L**	0.471*	0.397*		
**GDNF**	1.023e-08 n.s.			
**HGF**	0.262			
**IL-10**	0.098			
**IL-10RB**	0.373	0.202		
**IL-12B**	0.796	0.633		
**IL-15RA**	0.356	0.353	0.276	0.242
**IL-17A**	1.058e-08 n.s.			
**IL-17C**	0.172			
**IL-18**	0.557			
**IL-18R1**	0.713	0.475	0.393	
**IL-6**	0.161			
**IL-7**	0.052 n.s.			
**IL-8**	0.131			
**TGFB1**	0.229*	0.163*		
**LIF-R**	0.224			
**MCP-1**	0.437*	0.413*		
**MCP-2**	0.634	0.430	0.447	0.399

**MCP-3**	0.327	0.318		
**MCP-4**	0.755	0.566		
**MMP-1**	0.558*	0.468*	0.350*	
**MMP-10**	0.308			
**NT-3**	1.088e-08			
**OPG**	0.348			
**OSM**	0.271			
**SCF**	0.416*	0.408*	0.372*	
**SIRT2**	0.059 n.s.			
**SLAMF1**	0.203			
**ST1A1**	0.299	0.268	0.225	
**STAMPB**	0.055 n.s.	0.098 n.s.		
**TGF-alpha**	0.034 n.s.			
**TNFB**	0.406	0.268	0.258	0.241
**TNFRSF9**	0.362			
**TNFSF14**	0.317	0.273	0.182	
**TRAIL**	0.149 n.s.*	0.136*	0.134*	
**TRANCE**	0.419			
**TWEAK**	0.315			
**uPA**	0.185	0.165		
**VEGF-A**	0.405	0.310		

* The heritability estimates are from ONC_CVD

Table S6. Replication of top SNVs in NSPHS WGS GWAS to published genotypes associated to the same biomarker in a Finnish population[1]. Cytokines that were analyzed in both studies are presented.

Biomarker	Top SNV in NSPHS	chr:pos[†]	MAF	P NSPHS	P SNV from NSPHS in Ahola-Olli *et al*	Top SNV in Ahola-Olli *et al*	chr:pos[†]	P Ahola-Olli	R^2[××]
**bNGF**	no assoc**	-	-	-	-	no assoc**	-	-	-
**CCL11**	rs2228467	3:42906116	0.070	2.188E-09	2.271E-46	rs2228467	3:42906116	2.271E-46	1
**CCL3**	no assoc**	-	-	-	-	no assoc**	-	-	-
**CCL4**[ǀ]	rs113010081	3:46457412	0.232	4.187E-38	3.85E-140	rs113010081	3:46457412	3.85E-140	1
**CCL4**[ǀ]	rs4141329*[¦]	17:34490448	0.472	1.550E-14	-	rs113877493	17:34812273	1.62E-173	0.10
**CXCL1**[¶]	rs3117604	4:74734668	0.331	2.457E-19	2.126e-37	rs508977	4:74762383	7.558E-42	0.72
**CXCL1**[¶]	no second assoc	-	-	-	-	rs12075	1:159175354	1.244E-55	-
**CXCL10**[|]	rs11548618	4:76943947	0.035	5.072E-47	0.780	rs141053179	4:77589911	2.806E-15	0.03
**CXCL9**[‡]	rs11548618	4:76943947	0.035	6.194E-10	0.865	rs55876513	4:76883698	8.231E-11	0.00
**HGF**	no assoc**	-	-	-	-	rs3748034	4:3446091	1.810E-10	-
**HGF**	no assoc**	-	-	-	-	rs5745687[×]	7:81359051	2.751E-14	-
**IL-10**	no assoc**	-	-	-	-	rs4349809	6:43924830	5.771E-67	-
**IL-13**	did not pass QC	-	-	-	-	rs9472168	6:43928985	1.081E-65	-
**IL-18**	no assoc**	-	-	-	-	rs385076[×]	2:32489851	1.659E-22	-
**IL-18**	no assoc**	-	-	-	-	rs17229943[×]	5:68682536	1.617E-11	-
**IL-18**	no assoc**	-	-	-	-	rs71478720	11:112009605	3.708E-22	-
**IL-4**	did not pass QC	-	-	-	-	no assoc**	-	-	-
**IL-5**	did not pass QC	-	-	-	-	no assoc**	-	-	-
**IL-6**	no assoc**	-	-	-	-	no assoc**	-	-	-
**IL-7**	no assoc**	-	-	-	-	rs4320361	6:43928511	6.868E-39	-
**IL-8**	no assoc**	-	-	-	-	no assoc**	-	-	-
**MCP-1**	rs1800024*	3:46412559	0.075	1.257E-09	4.752e-19	rs138591554	3:46289206	7.943E-22	0.75
**MCP-1**	no second assoc	-	-	-	-	rs12075	1:159175354	1.435E-44	-
**MCP-3**	rs11102571	1:109407135	0.112	1.338E-09	0.651	no assoc**	-	-	-
**SCF**	rs6073958*	20:44551855	0.199	1.204E-09	0.028	rs1557570	1:169507844	2.739E-12	-
**TNFB**	rs2229092[¦]	6:31540757	0.027	2.699E-29	-	rs78655189	1:23046887	2.925E-32	-
**TRAIL**	rs144242131*	18:29769910	0.007	1.020E-12	1.78e-172	rs192989810	18:29783353	8.4E-175	0[§]

[†]In hg19 coordinates.
*Variant is from ONC_CVD. Either the p-value was lower, or no significant association was found in INF
[¦] not available / monomorphic in Ahola-Olli *et al*.
[ǀ] Called MIP1b in Ahola-Olli *et al.*
[¶]Called GROa in Ahola-Olli *et al.*
[|]Called IP10 in Ahola-Olli *et al.*
‡ Called MIG in Ahola-Olli *et al.*
[§]rs192989810 not available / monomorphic in NSPHS, LD taken from 1000G FIN
** no assoc - indicates that there was no genome-wide significant association in our cohort (P < 1.62e-08) or in the Finnish cohort (P < 1.2e-09)
[×]rs5745687, rs385076, rs17229943, rs71478720 were nominally associated with the same biomarker in our cohort (P = 3.6e-06, P= 0.00017, P= 3.1e-06, P = 2.7e-06, respectively)
[××] R^2 between the top variant found in the present study and Ahola-Olli *et al.*

Supplementary Table S7. Disease-associations for inflammatory diseases from the GWAS catalog. Each biomarker is presented with each GWAS variant as well as whether the variant was associated with a disease itself or in LD > 0.8 with a previously associated variant.

Disease trait	Biomarker	GWAS SNP	Effect	Chr	Pos (b37)	Associated SNP	OR	R^2	Effective allele of GWAS catalog SNP in our data	Effect of GWAS catalog SNP on biomarker level
**Ankylosing spondylitis**	IL-12B	rs10043720	-0.68	5	158,767,333	rs6556416-C	1.11	0.993	A	-0.68
**Asthma**	CCL19	rs149941420	0.61	6	32,556,454	rs7775228-A	1.17	0.849	C	0.60
**Celiac disease**	IL-18R1	rs10190555	1.08	2	102,994,056	rs13015714-C	1.28	0.991	G	1.08
**Celiac disease**	IL-18R1	rs10190555	1.08	2	102,994,056	rs917997-A	1.19	0.954	T	1.07
**Celiac disease**	IL-18R1	rs10190555	1.08	2	102,994,056	rs990171-A	1.20	0.954	A	1.07
**Chronic hepatitis B infection**	CD40	rs1569723	-0.84	20	44,742,064	rs1883832-T	1.19	1	T	-0.84
**Chronic hepatitis B infection**	CD40	rs4239702	-0.84	20	44,749,251	rs1883832-T	1.19	0.914	T	-0.86
**Chronic inflammatory diseases**	CD40	rs1569723	-0.84	20	44,742,064	rs1883832-?	-	1	T	-0.84
**Chronic inflammatory diseases**	CD40	rs4239702	-0.84	20	44,749,251	rs1883832-?	-	0.914	T	-0.86
**Chronic inflammatory diseases**	CD6	rs11230563	-0.78	11	60,776,209	rs11230563-?	-	1	T	-0.78
**Chronic inflammatory diseases**	IL-12B	rs10043720	-0.68	5	158,767,333	rs6556411-?	-	1	G	-0.68
**Crohn’s disease**	CD40	rs1569723	-0.84	20	44,742,064	rs1569723-?	-	1	C	-0.84

**Crohn's disease**	CD40	rs1569723	-0.84	20	44,742,064	rs6074022-G	1.08	1	C	-0.84
**Crohn's disease**	CD40	rs4239702	-0.84	20	44,749,251	rs1569723-?	-	0.914	C	-0.86
**Crohn's disease**	CD40	rs4239702	-0.84	20	44,749,251	rs6074022-G	1.08	0.914	C	-0.86
**Crohn's disease**	CD6	rs11230563	-0.78	11	60,776,209	rs11230563-G/C	1.08 / 1.09	1	T	-0.78
**Crohn's disease**	IL-12B	rs10043720	-0.68	5	158,767,333	rs10045431-C	1.11	0.884	A	-0.66
**Crohn's disease**	IL-18R1	rs10190555	1.08	2	102,994,056	rs2058660-G	1.19	0.954	G	1.07
**Crohn's disease**	IL-18R1	rs10190555	1.08	2	102,994,056	rs6708413-G	1.09	0.954	G	1.07
**Inflammatory bowel disease**	CCL4	rs113010081	0.80	3	46,457,412	rs113010081-G	1.10	1	C	0.80
**Inflammatory bowel disease**	CD40	rs1569723	-0.84	20	44,742,064	rs1569723-C	1.10	1	C	-0.84
**Inflammatory bowel disease**	CD40	rs1569723	-0.84	20	44,742,064	rs6074022-G	1.08	1	C	-0.84
**Inflammatory bowel disease**	CD40	rs4239702	-0.84	20	44,749,251	rs1569723-C	1.10	0.914	C	-0.86
**Inflammatory bowel disease**	CD40	rs4239702	-0.84	20	44,749,251	rs6074022-G	1.08	0.914	C	-0.86
**Inflammatory bowel disease**	CD6	rs11230563	-0.78	11	60,776,209	rs11230563-G	1.08	1	T	-0.78
**Inflammatory bowel disease**	CD6	rs11230563	-0.78	11	60,776,209	rs11230563-G	1.08	1	T	-0.78
**Inflammatory bowel disease**	IL-18R1	rs10190555	1.08	2	102,994,056	rs6708413-G	1.09	0.954	G	1.07

**Inflammatory bowel disease**	IL-18R1	rs10190555	1.08	2	102,994,056	rs917997-T	1.10	0.954	T	1.07
**Juvenile idiopathic arthritis**	CCL19	rs149941420	0.61	6	32,556,454	rs7775055-G	6.01	0.965	C	0.14
**Juvenile idiopathic arthritis**	CCL4	rs113010081	0.80	3	46,457,412	rs79893749-?	1.28	0.821	T	0.75
**Kawasaki syndrome**	CD40	rs1569723	-0.84	20	44,742,064	rs1569723-A	1.42	1	C	-0.84
**Kawasaki syndrome**	CD40	rs4239702	-0.84	20	44,749,251	rs1569723-A	1.42	0.914	C	-0.86
**Multiple sclerosis**	CD40	rs1569723	-0.84	20	44,742,064	rs2425752-A	1.11	0.926	T	-0.80
**Multiple sclerosis**	CD40	rs1569723	-0.84	20	44,742,064	rs4810485-A	1.11	0.992	T	-0.84
**Multiple sclerosis**	CD40	rs1569723	-0.84	20	44,742,064	rs6074022-G/C	1.20 / 1.15	1	C	-0.84
**Multiple sclerosis**	CD40	rs4239702	-0.84	20	44,749,251	rs2425752-A	1.11	0.843	T	-0.83
**Multiple sclerosis**	CD40	rs4239702	-0.84	20	44,749,251	rs4810485-A	1.11	0.906	T	-0.85
**Multiple sclerosis**	CD40	rs4239702	-0.84	20	44,749,251	rs6074022-G/C	1.20 / 1.15	0.914	C	-0.86
**Multiple sclerosis**	TNFSF14	rs2291668	0.25	19	6,669,934	rs1077667-G	1.16	0.879	T	0.23
**Pediatric autoimmune diseases**	IL-18R1	rs10190555	1.08	2	102,994,056	rs2075184-T	-	0.954	T	1.07
**Rheumatoid arthritis**	CCL19	rs149941420	0.61	6	32,556,454	rs12525220-A	3.02	0.965	A	0.59
**Rheumatoid arthritis**	CD40	rs1569723	-0.84	20	44,742,064	rs4239702-C	1.14	0.914	T	-0.80

**Rheumatoid arthritis**	CD40	rs1569723	-0.84	20	44,742,064	rs4810485-T/G	1.18 / 1.15	0.992	T	-0.84
**Rheumatoid arthritis**	CD40	rs4239702	-0.84	20	44,749,251	rs4239702-C	1.14	1	T	-0.84
**Rheumatoid arthritis**	CD40	rs4239702	-0.84	20	44,749,251	rs4810485-T/G	1.18 / 1.15	0.906	T	-0.85
**Rheumatoid arthritis**	CD40	rs1569723	-0.84	20	44,742,064	rs1883832-G	1.34	1	T	-0.84
**Rheumatoid arthritis**	CD40	rs1569723	-0.84	20	44,742,064	rs4810485-T/G	1.18 /1.15	0.992	T	-0.84
**Rheumatoid arthritis**	CD40	rs1569723	-0.84	20	44,742,064	rs6032662-?	1.17	1	C	-0.84
**Rheumatoid arthritis**	CD40	rs4239702	-0.84	20	44,749,251	rs1883832-G	1.34	0.914	T	-0.86
**Rheumatoid arthritis**	CD40	rs4239702	-0.84	20	44,749,251	rs4810485-T/G	1.18 / 1.15	0.906	T	-0.85
**Rheumatoid arthritis**	CD40	rs4239702	-0.84	20	44,749,251	rs6032662-?	1.17	0.914	C	-0.86
**Systemic lupus erythematosus**	CD40	rs1569723	-0.84	20	44,742,064	rs4810485-A	1.43	0.992	T	-0.84
**Systemic lupus erythematosus**	CD40	rs4239702	-0.84	20	44,749,251	rs4810485-A	1.43	0.906	T	-0.85
**Ulcerative colitis**	CD6	rs11230563	-0.78	11	60,776,209	rs11230563-G	1.08	1	T	-0.78
**Ulcerative colitis**	CXCL5	rs352045	0.95	4	74,864,687	rs2457996-A	1.11	0.944	C	0.96
**Ulcerative colitis**	CXCL5	rs425535	1.00	4	74,863,997	rs2457996-A	1.11	0.944	C	1.00

Supplementary Table S8. Comparison of top variants for each biomarker with published data in the GWAS catalog. Blood-trait-associations for the inflammatory biomarkers, where associations to protein levels and cell counts are presented. If the variant has been reported in the catalog before, it is marked in bold. The other variants have not been previously reported, but are in strong LD with variants that have.

Biomarker	Trait	Mapped trait	SNV	Annotation	Associated SNP (GWAS catalog)	R^2
**CCL11**	Blood protein levels; CCL2, eotaxin levels; Eosinophil, monocyte, myeloid white cell, sum eosinophil basophil counts; Eosinophil, granulocyte, monocyte, neutrophil percentages	Basophil, eosinophil, monocyte, myeloid white cell counts; Blood protein measurement; CCL17, CCL2, eotaxin measurement; Eosinophil, granulocyte, monocyte, neutrophil percentages	**rs2228467**	missense	rs2228467	1
**CCL19**	Blood protein levels	Blood protein, CCL19 measurements	rs149941420	intronic (*HLA-DRB1*)	rs116763083, rs139025277, rs7775228, rs114991247	0.965, 0.872, 0.849, 0.965
**CCL20***	Blood protein levels	Blood protein, MMP12 measurements	rs17368659	intronic (*MMP12*)	rs17368582, rs28381684	1, 1
**CCL23**	Blood protein levels	Blood protein measurements	**rs712048**	ncRNA_intronic (*CCL15-CCL14*)	rs712048	1
**CCL4**	CCL4 & blood protein levels	CCL4 measurement	**rs113010081**	intergenic (*CCRL2*;*LTF*)	rs113010081, rs113341849, rs79815064, rs6808835	1, 0.980, 0.970, 0.896
**CST-5**	Blood protein levels	CST5 measurement	**rs6138152**	intergenic (*CST2*;*CST5*)	rs6138152	1
**CXCL1**	Blood protein levels; Sum neutrophil eosinophil, white blood cell counts; Granulocyte, monocyte percentages	Blood protein, CXCL6 measurement; CXCL1 measurement; Leukocyte, neutrophil, eosinophil counts; Granulocyte, monocyte percentages	rs10938101, rs3117604	intergenic (*CXCL8*;*CXCL6*), upstream (*CXCL1*)	rs16850073, rs3117600, rs3117603	0.801, 0.858, 0.818
**CXCL10**	Blood protein levels	Blood protein, CXCL10 measurements	**rs11548618**	missense (*CXCL10*)	rs11548618	1
**CXCL5**	Blood protein levels; IL-10, IL-12p70 & VEGF levels; Mean platelet volume, Myeloid white cell, platelet, sum basophil neutrophil counts; Neutrophil percentages	Blood protein, CXCL5, IL-10, IL-12, VEGF measurements; Mean platelet volume; Myeloid white cell, neutrophil, basophil, platelet counts; Neutrophil percentages	rs352045, **rs425535**, **rs7088799**, rs10740118	upstream (*CXCL5*), missense (*CXCL5*), intronic (*JMJD1C*), intronic (*JMJD1C*)	rs425535, rs10761731, rs10761741, rs7080386, rs7075195, rs7896518, rs7080386	1, 1, 1, 0.993, 1, 1, 0.993
**CXCL6**	Eosinophil, granulocyte, monocyte, neutrophil percentages; Sum neutrophil eosinophil, white blood cell counts	CXCL6 measurements, Eosinophil, granulocyte, monocyte, neutrophil percentages; Leukocyte, neutrophil, eosinophil counts;	rs111903579	intergenic (*CXCL8*;*CXCL6*)	rs16850073, rs13148728	1, 0.810
**FGF-5**	Red blood cell count	Erythrocyte count	rs16998073	intergenic (*PRDM8*;*FGF5*)	rs10857147, rs12509595	0.985, 0.985
**Flt3L**	Monocyte, myeloid white cell & white blood cell counts; Granulocyte, monocyte percentages	Leukocyte, monocyte, myeloid white cell counts; Granulocyte, monocyte percentages	rs145096717, rs111595024	intronic (*PAN3*), intronic (*PAN3*)	rs76428106	1, 0.886
**IL-12B**	Blood protein levels	Blood protein measurements	rs10043720	ncRNA_intronic (LOC285626)	rs4921484	1
**IL-18R1**	Blood protein levels; Lymphocyte counts; Lymphocyte percentages; Serum protein levels	Blood protein, IL-18R1, serum ST2 measurements; Lymphocyte counts and percentages	rs10190555, rs12999517	intronic (*IL18R1*), intronic (*IL1RL1*)	rs1420106, rs2001461, rs2058622, rs990171, rs2058660, rs12999542, rs13014644	1, 1, 0.990, 0.954, 0.954, 1, 1,

**MCP-1***	Granulocyte percentages; Monocyte counts; CCL2 levels	Granulocyte percentages; Monocyte counts; CCL2 measurements	**rs1800024**	ncRNA_intronic (*LOC102724297*)	rs1800024, rs62246129, rs1799864, rs62242983	1, 0.865, 0.977, 0.977
**MCP-2**	Blood protein levels	Blood protein, CCL7, CCL8 measurements	**rs1133763**, rs74832623	missense (*CCL8*), intergenic (*LINC01989;CCL2*)	rs1133763, rs3138036, rs34202026	1, 1, 1
**MCP-4**	Blood protein levels; Basophil percentages; Monocyte, myeloid white cell, white blood cell counts; Eotaxin, IL-8, CCL2 levels	Blood protein, CCL2, eotaxin, IL-8 measurements; Basophil, leukocyte, monocyte, myeloid white cell counts; Basophil percentages;	**rs12075**	missense (*ACKR1*)	rs12075	1
**TNFSF14**	Granulocyte, monocyte percentages; Monocyte counts	Granulocyte, monocyte percentages; Monocyte counts	rs2291668 (secondary)	synonymous (*TNFSF14*)	rs1077667, rs12461821	0.879, 1
**VEGF-A**	Blood protein levels, IL-10, IL-12p70, IL-13, IL-5, IL-7 levels & VEGF levels	Blood protein, IL-5, IL-7, IL-10, IL-12, IL-13, VEGF measurements	**rs6921438**	intergenic (*LINC01512*; *LOC101929705*)	rs4513773, rs6921438, rs11757903, rs4349809, rs9472168, rs7767396, rs4320361	0.933, 1, 0.892, 0.933, 0.933, 0.933, 0.933

Figures

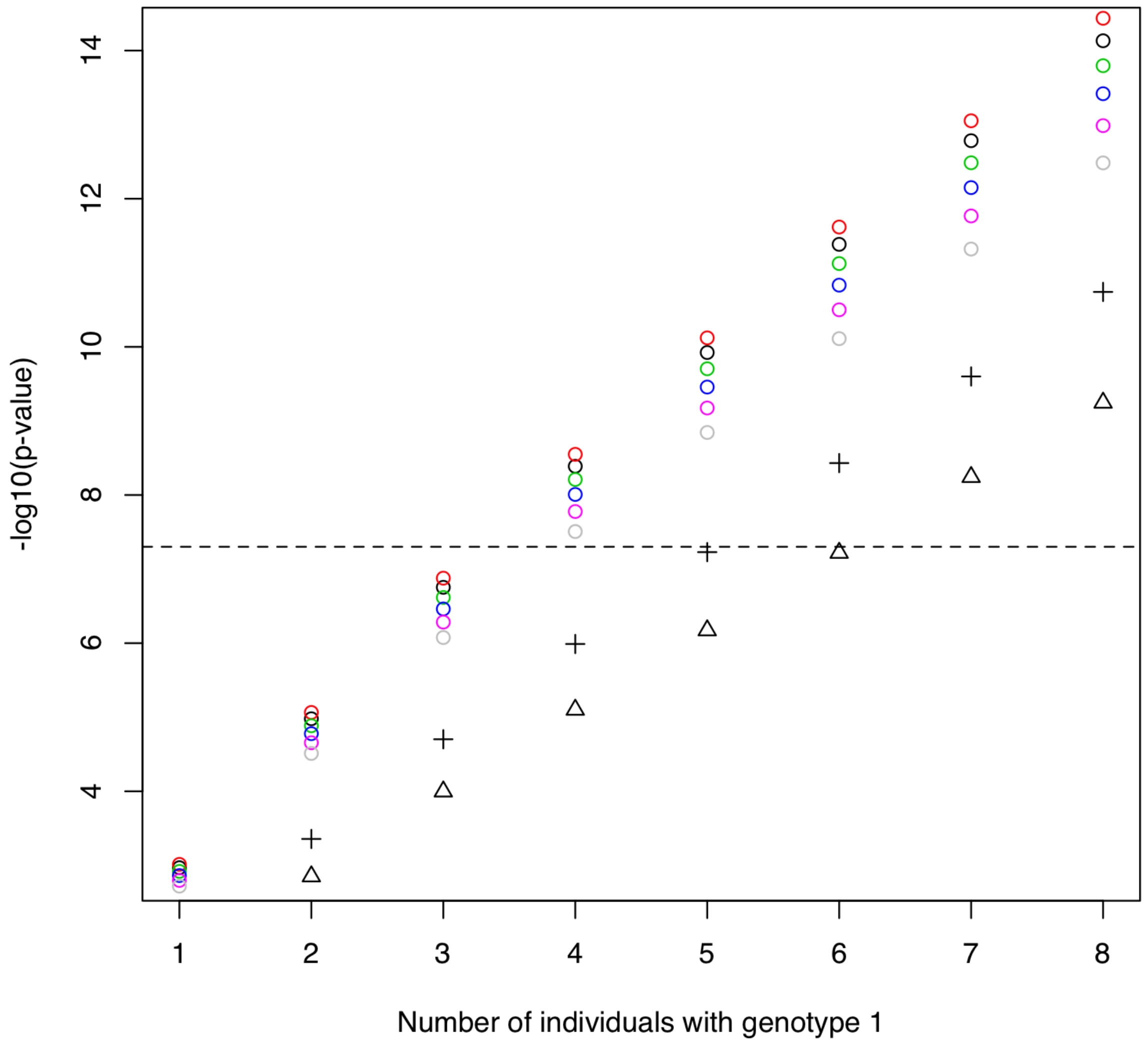

Supplementary Figure S1. Plot of the simulation to find a suitable MAF cutoff. The p-values (-log10) are plotted against the number of individuals with genotype 1, i.e. a genotype giving rise to an extreme biomarker concentration. The rest of the individuals are assumed to have genotype 0 and the biomarker levels are assumed to be rank-transformed. The total number of individuals are coded as follows: red – 1000, black – 900, green – 800, blue – 700, magenta – 600 and grey – 500. The standard genome-wide significance of $5x10^{-8}$ is represented by the dashed line. The plus signs (+) and the triangles (Δ) represents cases with n=900 when the individuals with genotype 1 does not have the most extreme concentrations. The number of individuals in this study with WGS data is 1021 of which up to 957 in ONC_CVD and 893 in INF have biomarker data. A MAF of 0.15% (3 alleles in 1021 individuals) was therefore used since 4 alleles with extreme biomarker concentrations are detectable in down to 800 individuals with our genome-wide significance of $1.62x10^{-8}$.

Supplementary Figures S2-S20. Locuszoom plots for the loci where at least one significant hit was found with WGS data but not with genotyped/imputed data. The regional plots to the right are for the WGS data where the ones only denoted "regional plot" is the INF panel. The ones denoted "regional plot technical replicate" is the ONC_CVD panel. All graphs have colored dots based on the LD data information from 1000G nov 2014 EUR dataset. Grey dots depict when no usable LD information could be found for the reference SNP or other SNPs in relation to the reference SNP.

Supplementary Figure S2. Regional association plots for ADA. Imputed data to the left and WGS data to the right. The lead variant (rs11555566) is in high LD with other variants in the 1000G data (red points in the plot). However, rs11555566 has a slightly lower frequency (1.9%) in our cohort compared to the 1000G cohorts 6.6%) and the LD with other SNVs is much lower, which could explain the less accurate imputation of rs11555566 and the lack of other SVNs with genome wide significant P-values in the region.

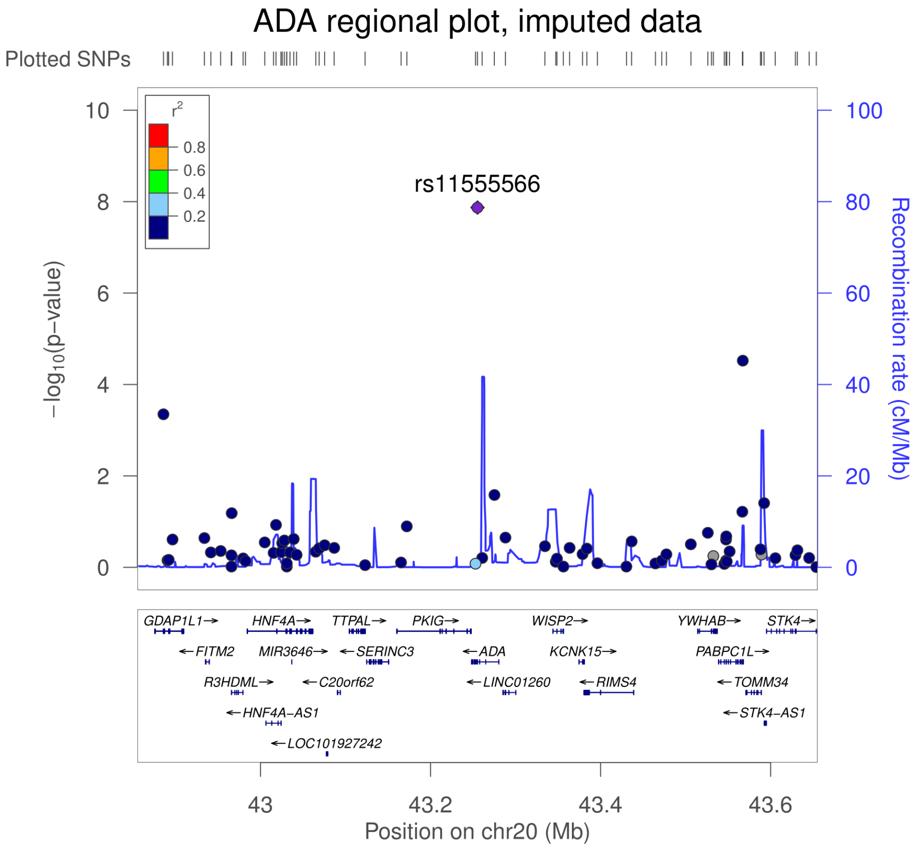

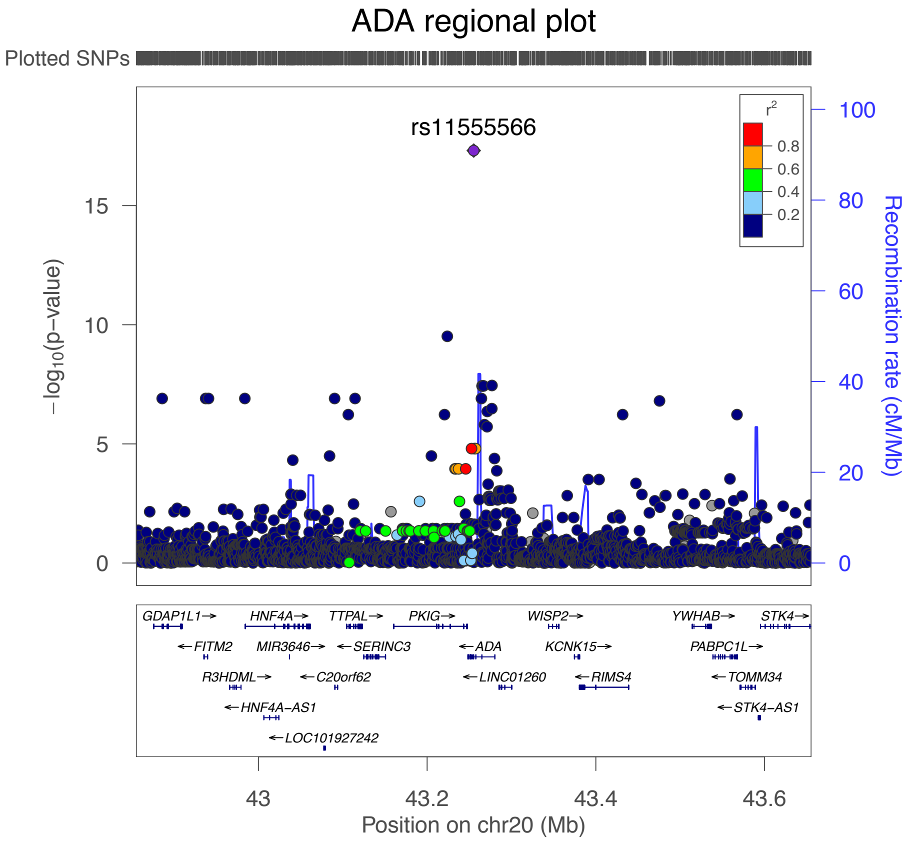

Supplementary Figure S3. Regional association plots for CASP-8. Imputed data to the left, WGS data from INF in the middle and from ONC_CVD, the technical replicate, to the right.

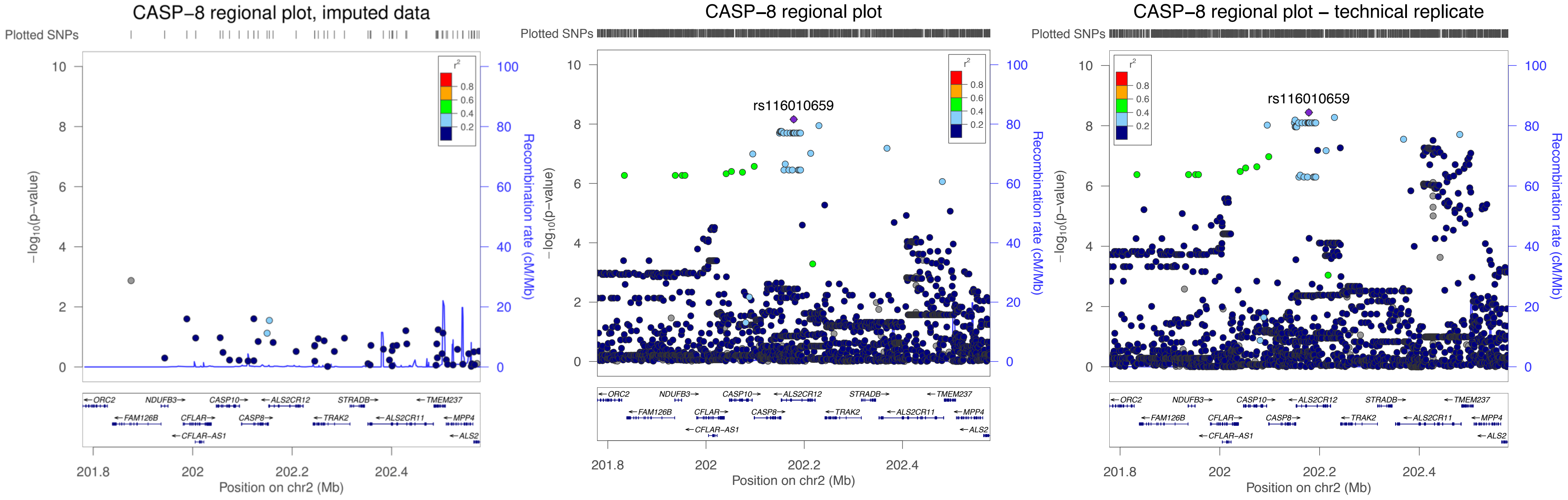

Supplementary Figure S4. Regional association plots for CCL11. Imputed data to the left and WGS data to the right.

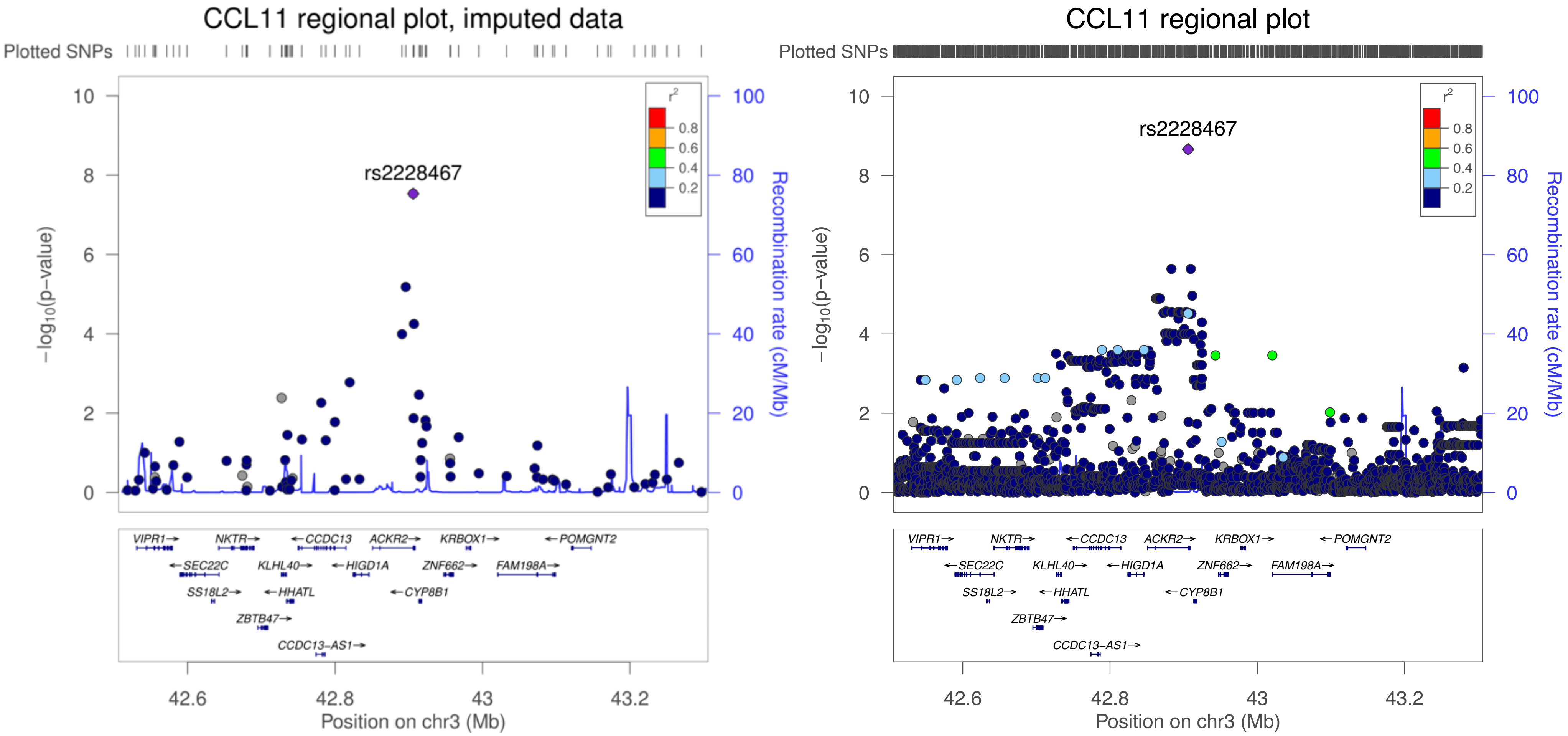

Supplementary Figure S5. Regional association plots for CCL20. Imputed data to the left and WGS data to the right.

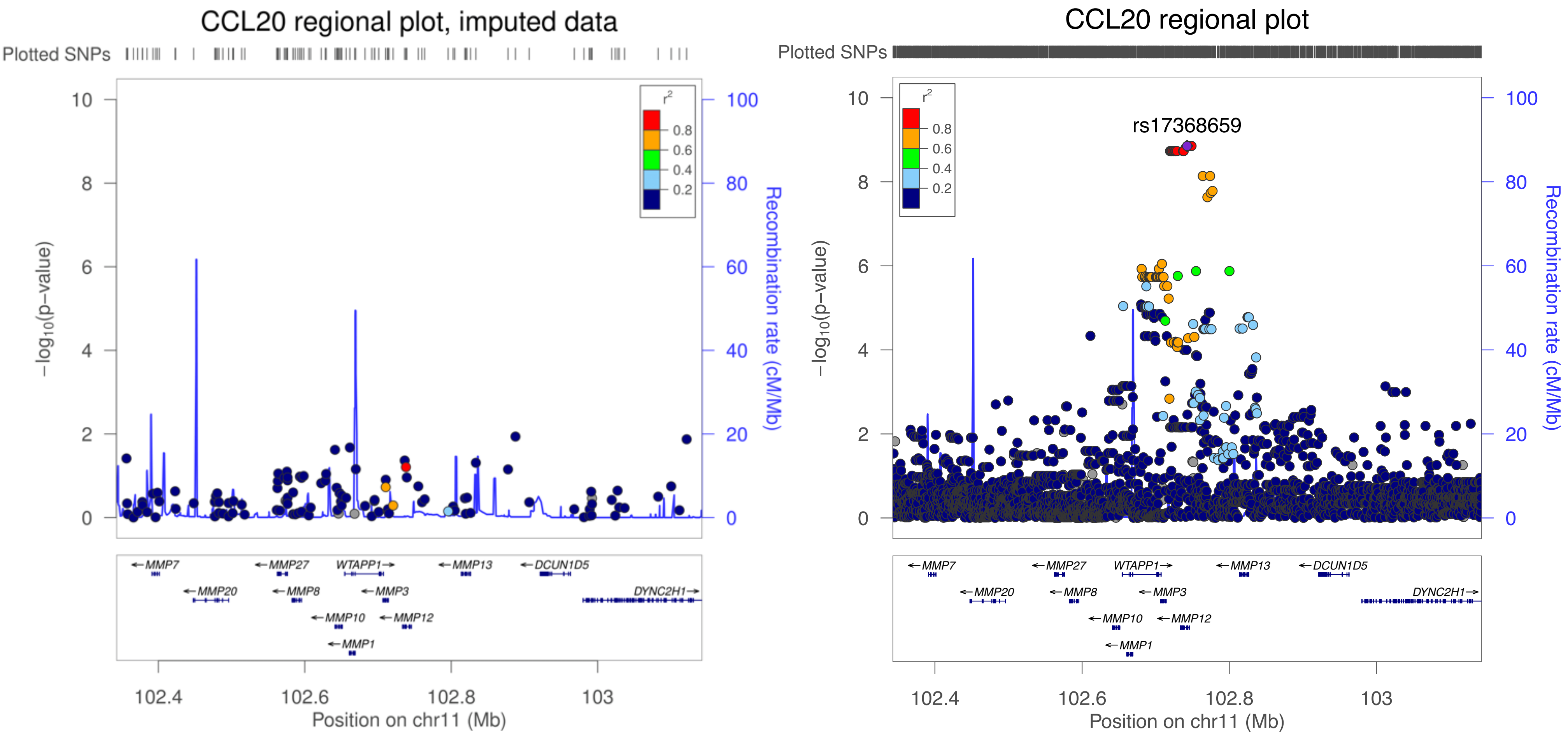

Supplementary Figure S6. Regional association plots for CCL23. Imputed data to the left and WGS data to the right. When analyzing the imputed data, CCL23 only had a significant association (shown in the plot below) in the smaller sub-cohort (recruited in 2009) and not in the other sub-cohort (recruited in 2006). This biomarker was therefore note used in the replication step[2] and was not counted as a significant association.

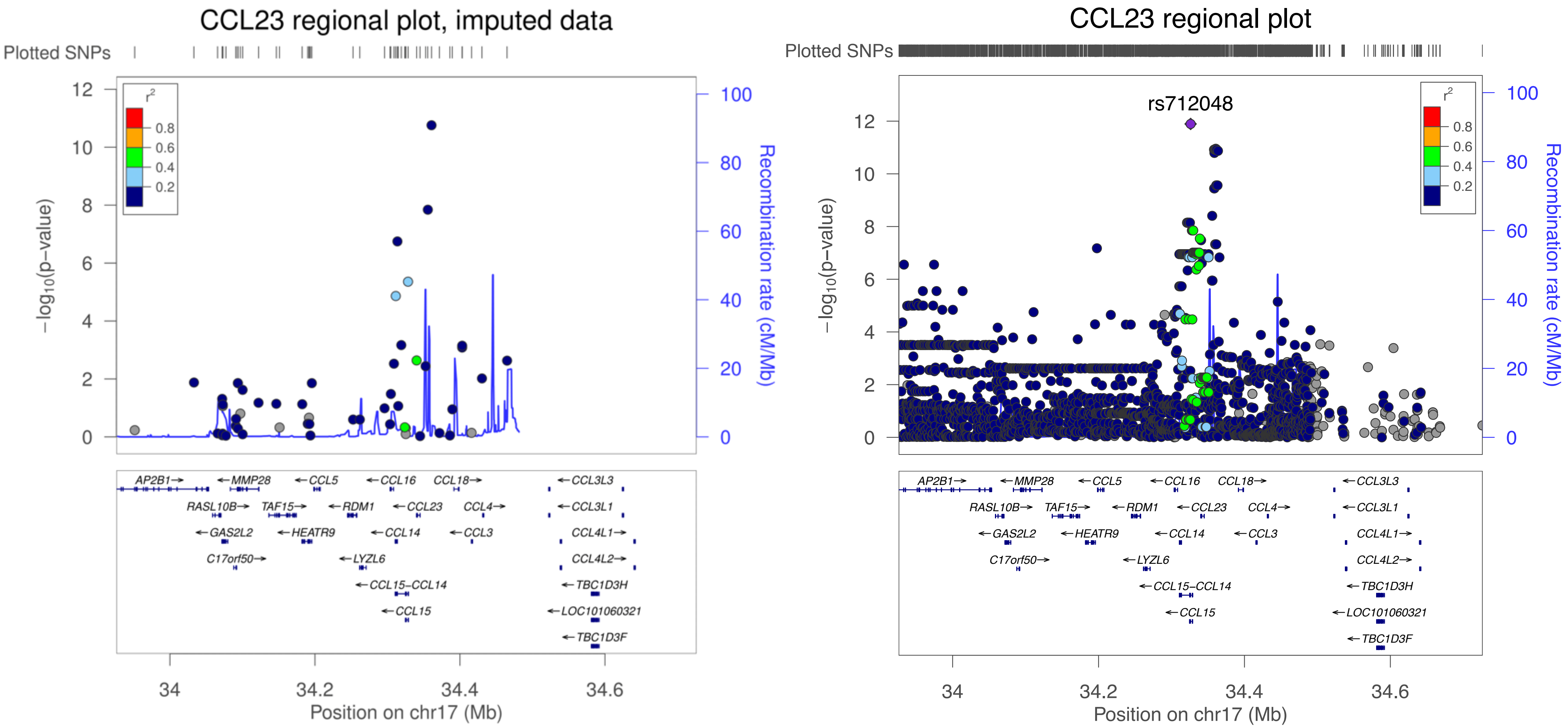

Supplementary Figure S7. Regional association plots for CD244. Imputed data to the left and WGS data to the right. No usable LD information could be loaded for the reference SNP.

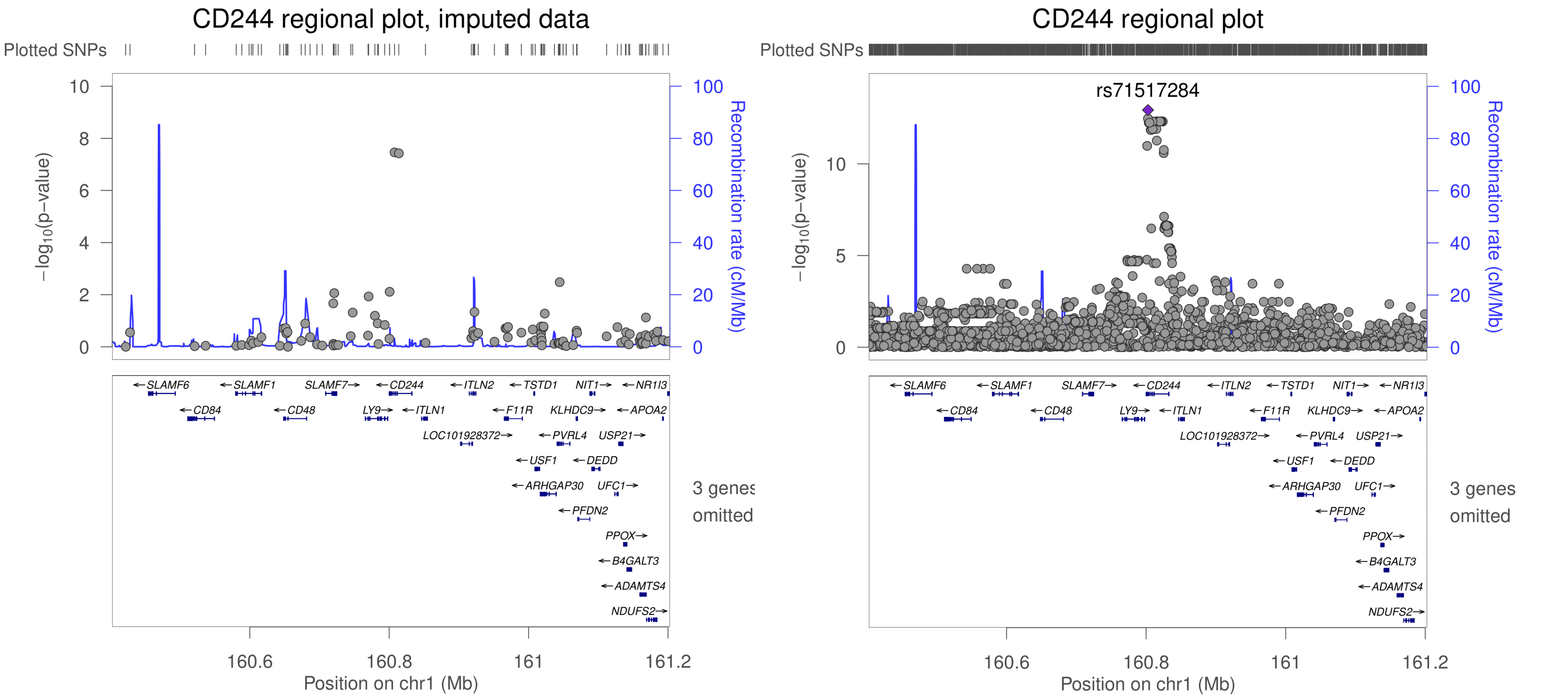

Supplementary Figure S8. Regional association plots for CDCP1. Imputed data to the left and WGS data to the right.

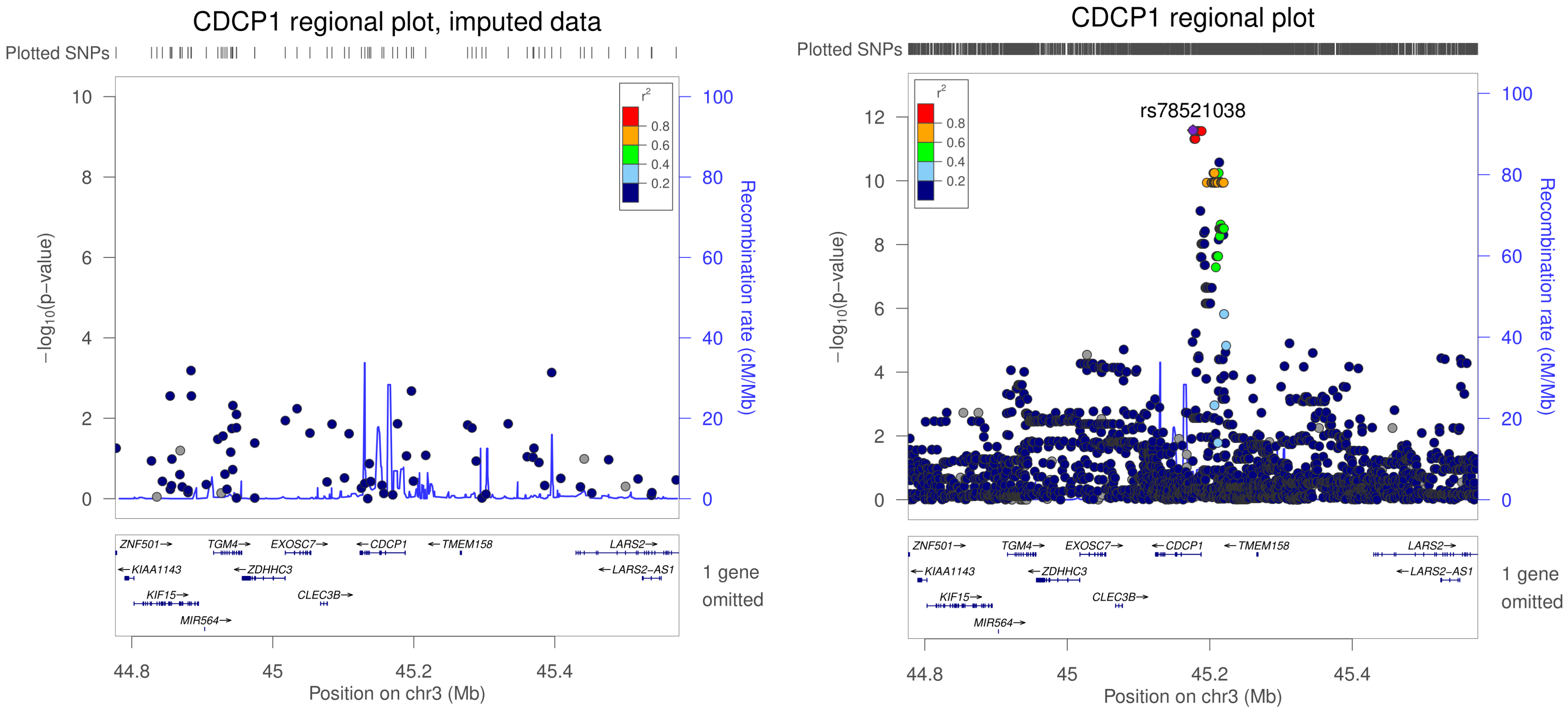

Supplementary Figure S9. Regional association plots for CX3CL1. Imputed data to the left and WGS data to the right.

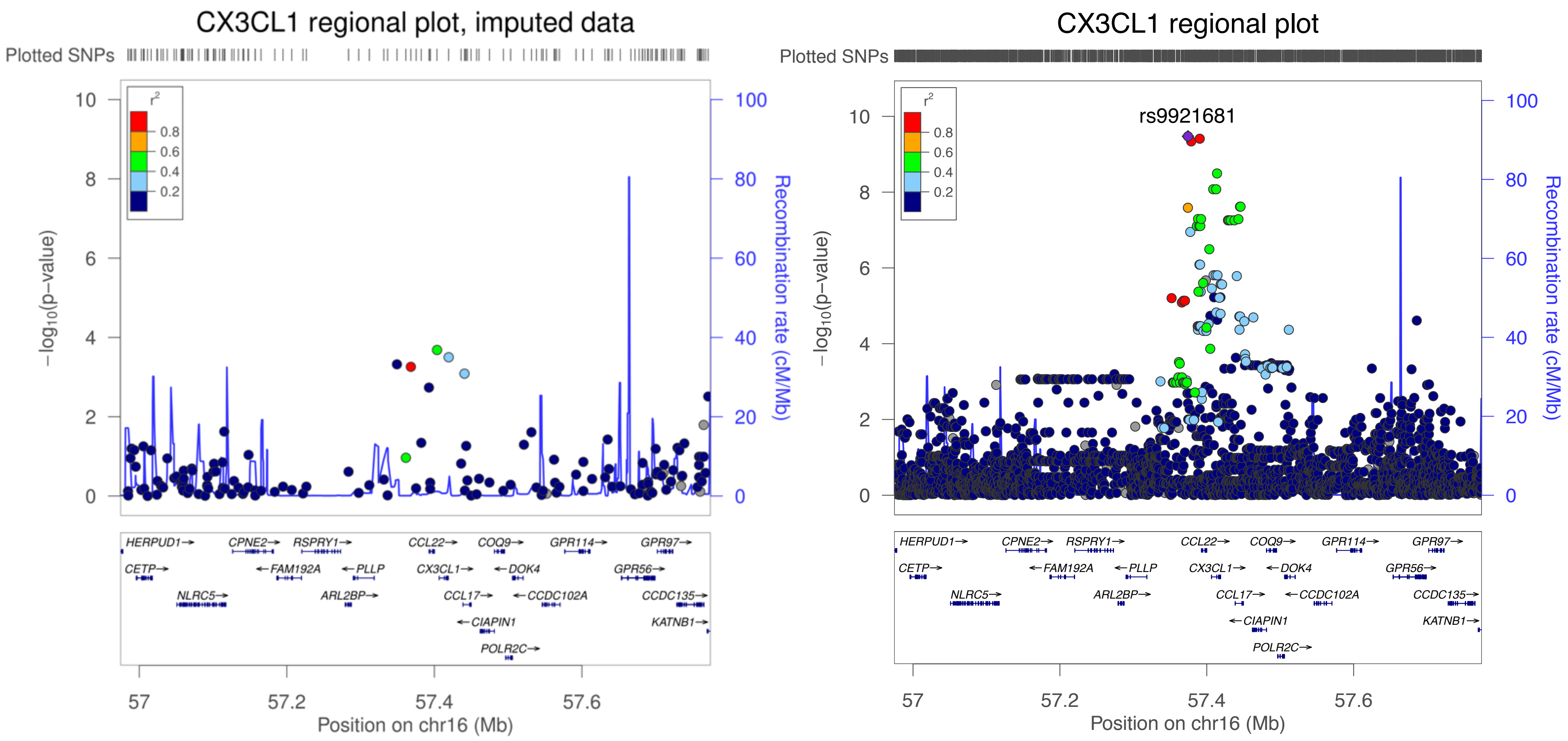

Supplementary Figure S10. Regional association plots for CXCL1. Imputed data to the left, WGS data from INF in the middle and from ONC_CVD, the technical replicate, to the right.

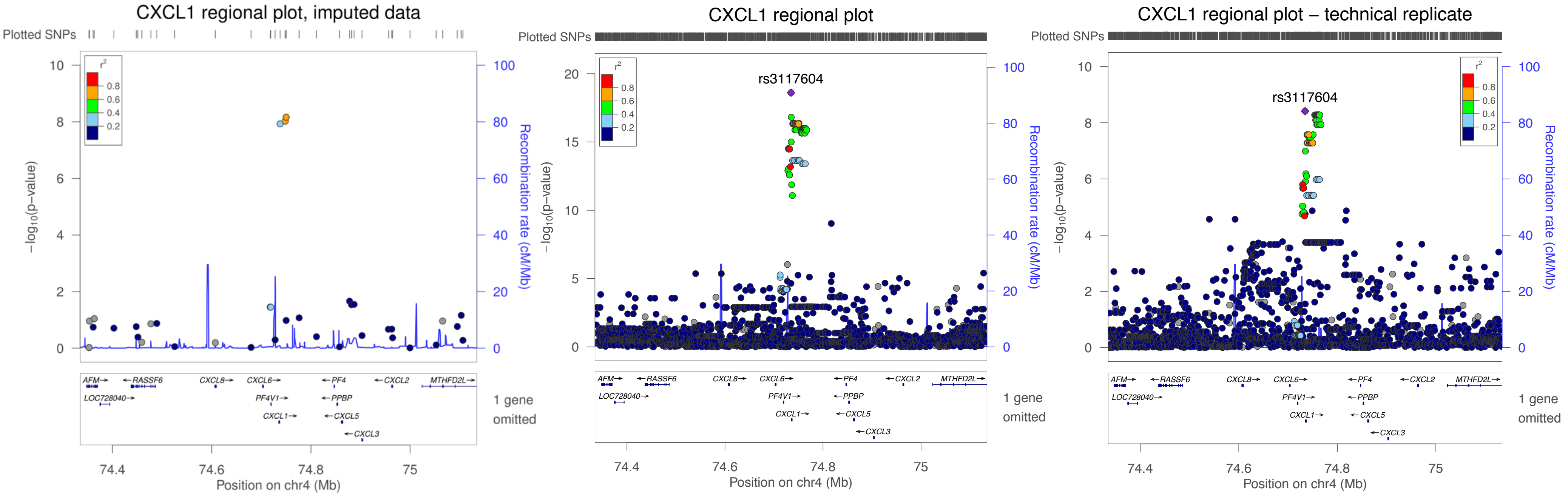

Supplementary Figure S11. Regional association plots for CXCL9. Imputed data to the left and WGS data to the right. As discussed below, rs11548618 is most likely a false positive association and has not been considered a novel finding in this study.

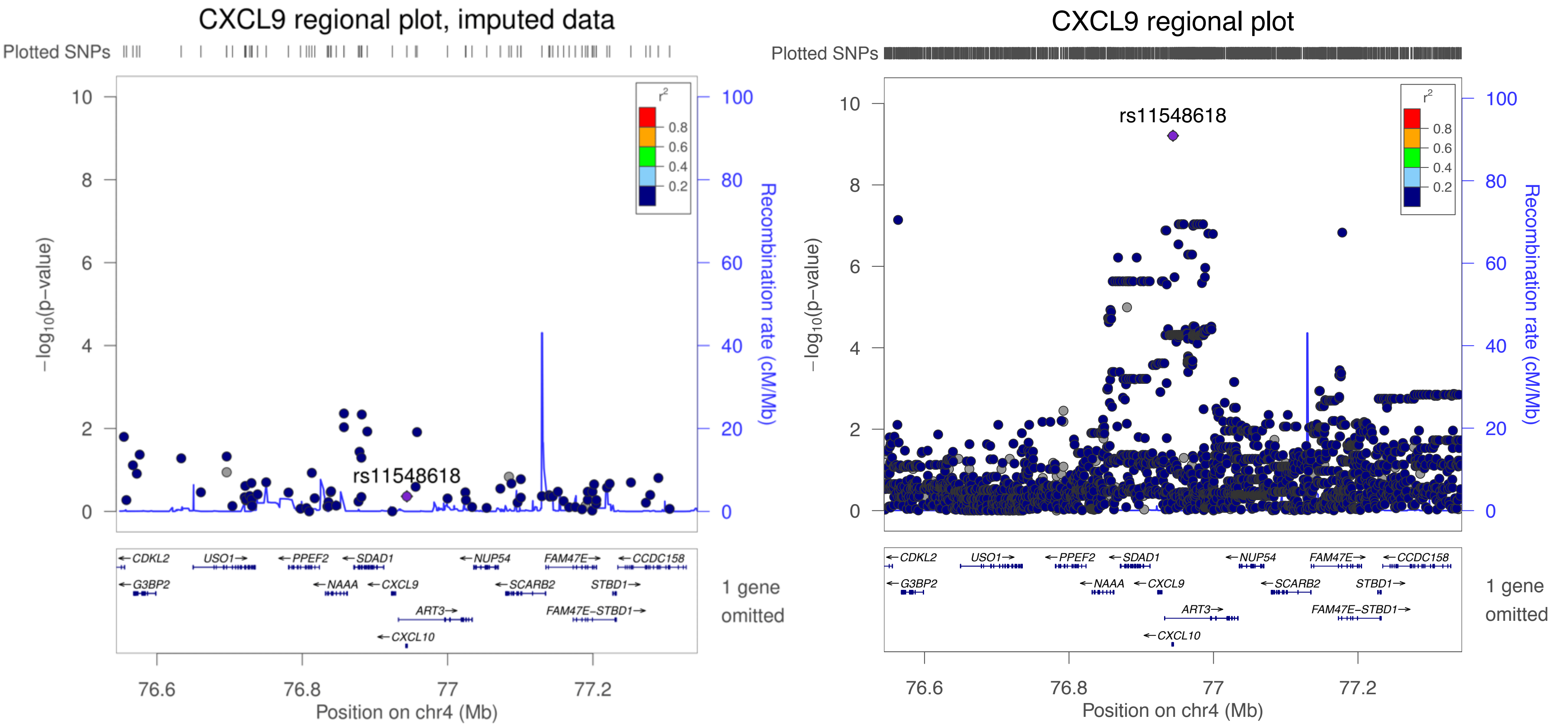

Supplementary Figure S12. Regional association plots for CXCL11. Imputed data to the left and WGS data to the right. As discussed below, rs11548618 is most likely a false positive association and has not been considered a novel finding in this study.

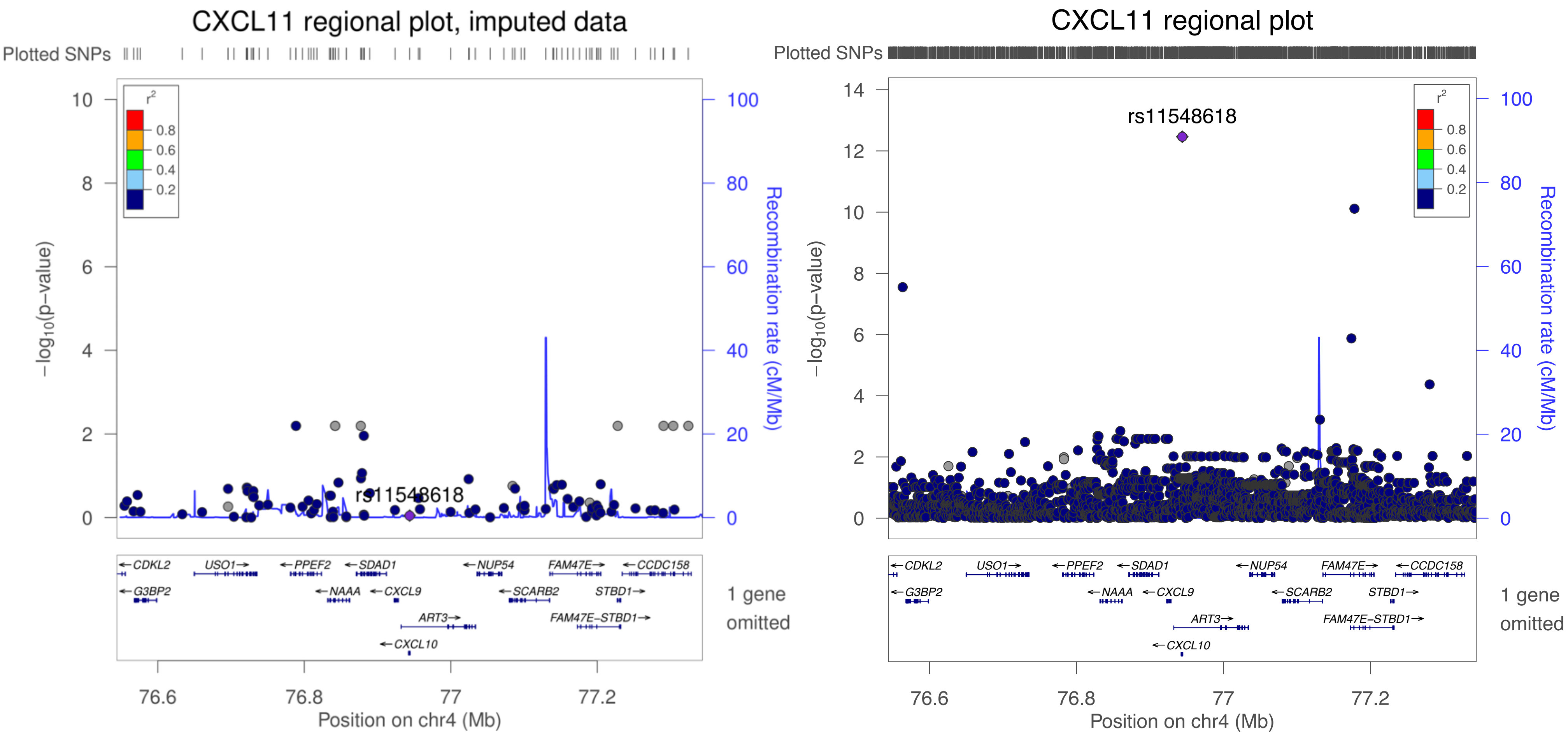

Supplementary Figure S13. Regional association plots for FGF-5. Imputed data to the left and WGS data to the right.

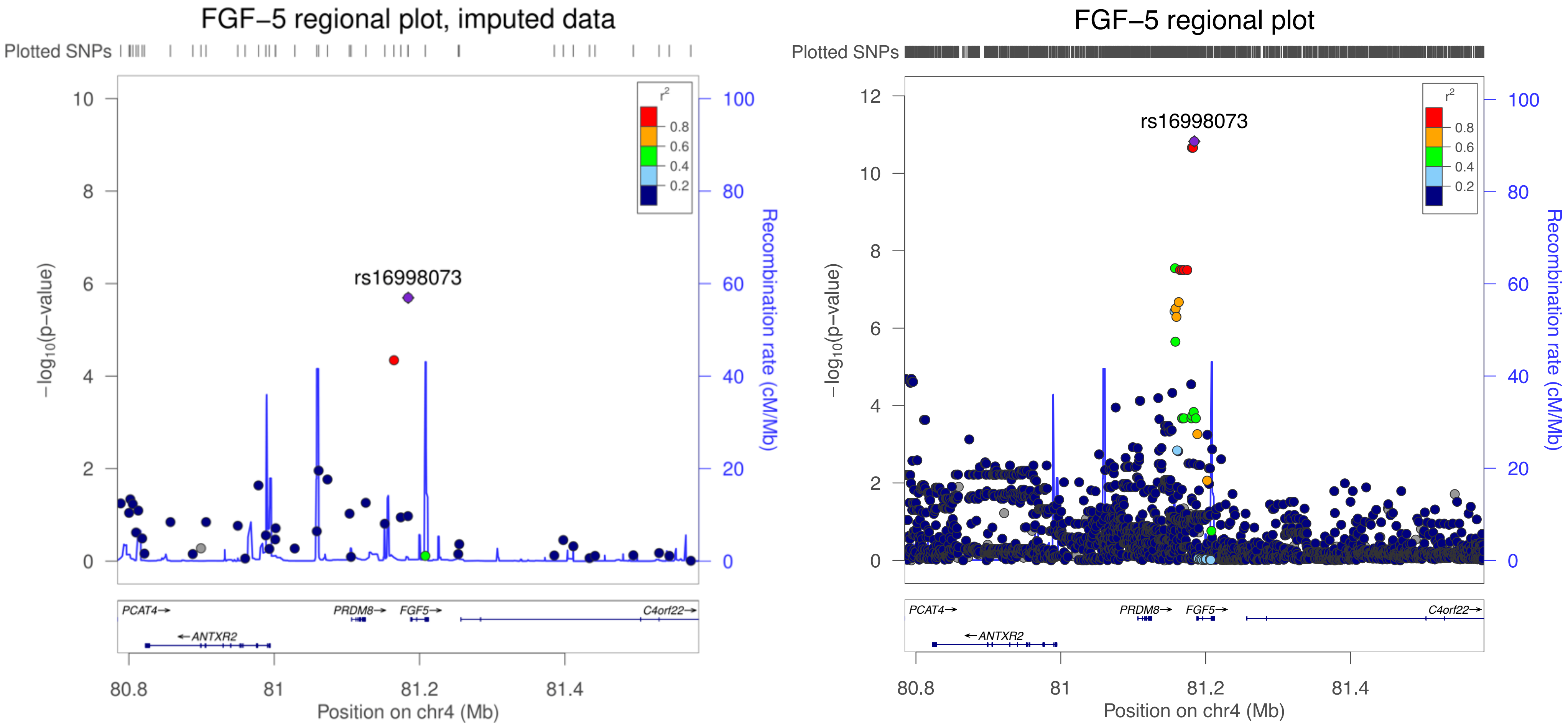

Supplementary Figure S14. Regional association plots for MCP-3. Imputed data to the left and WGS data to the right.

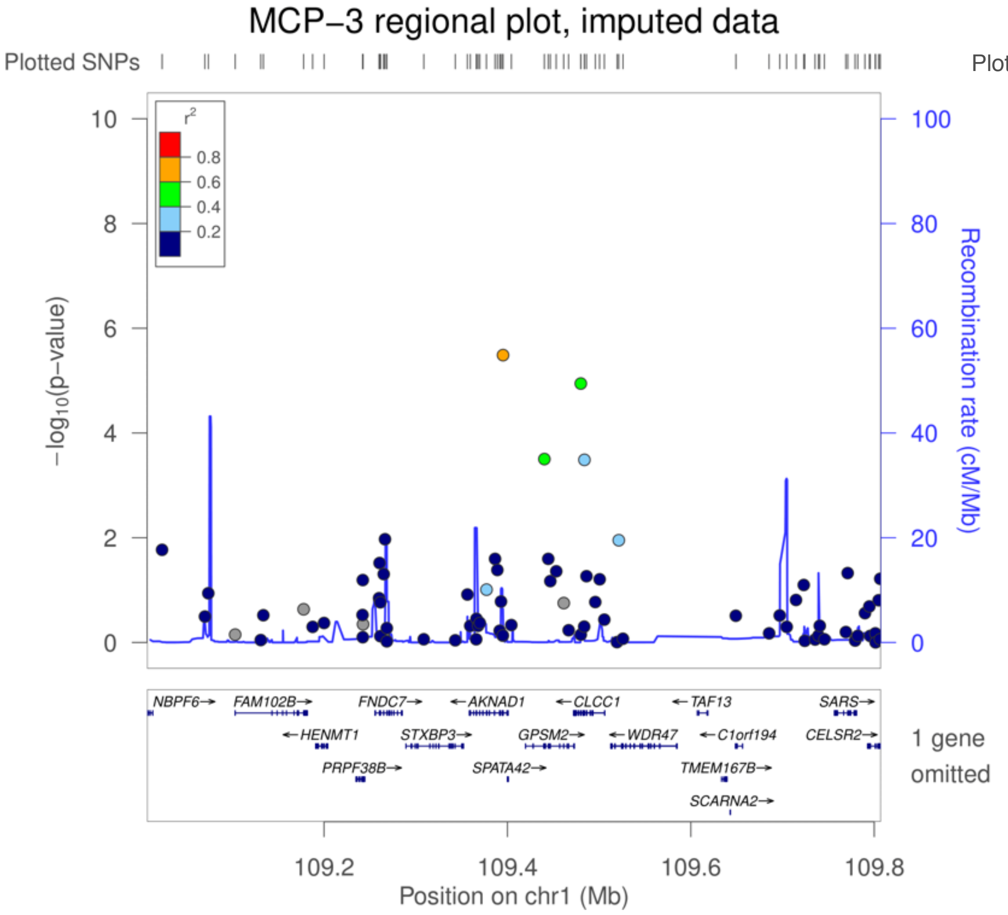

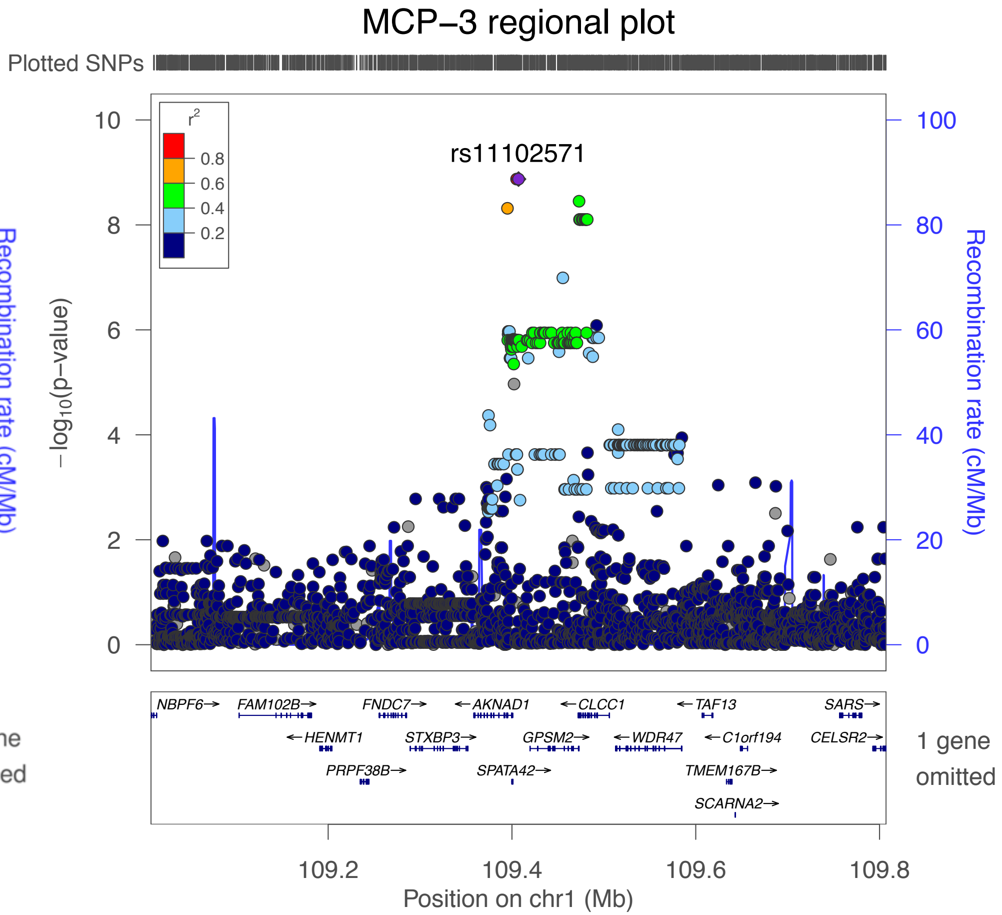

Supplementary Figure S15. Regional association plots for ST1A1. Imputed data to the left and WGS data to the right.

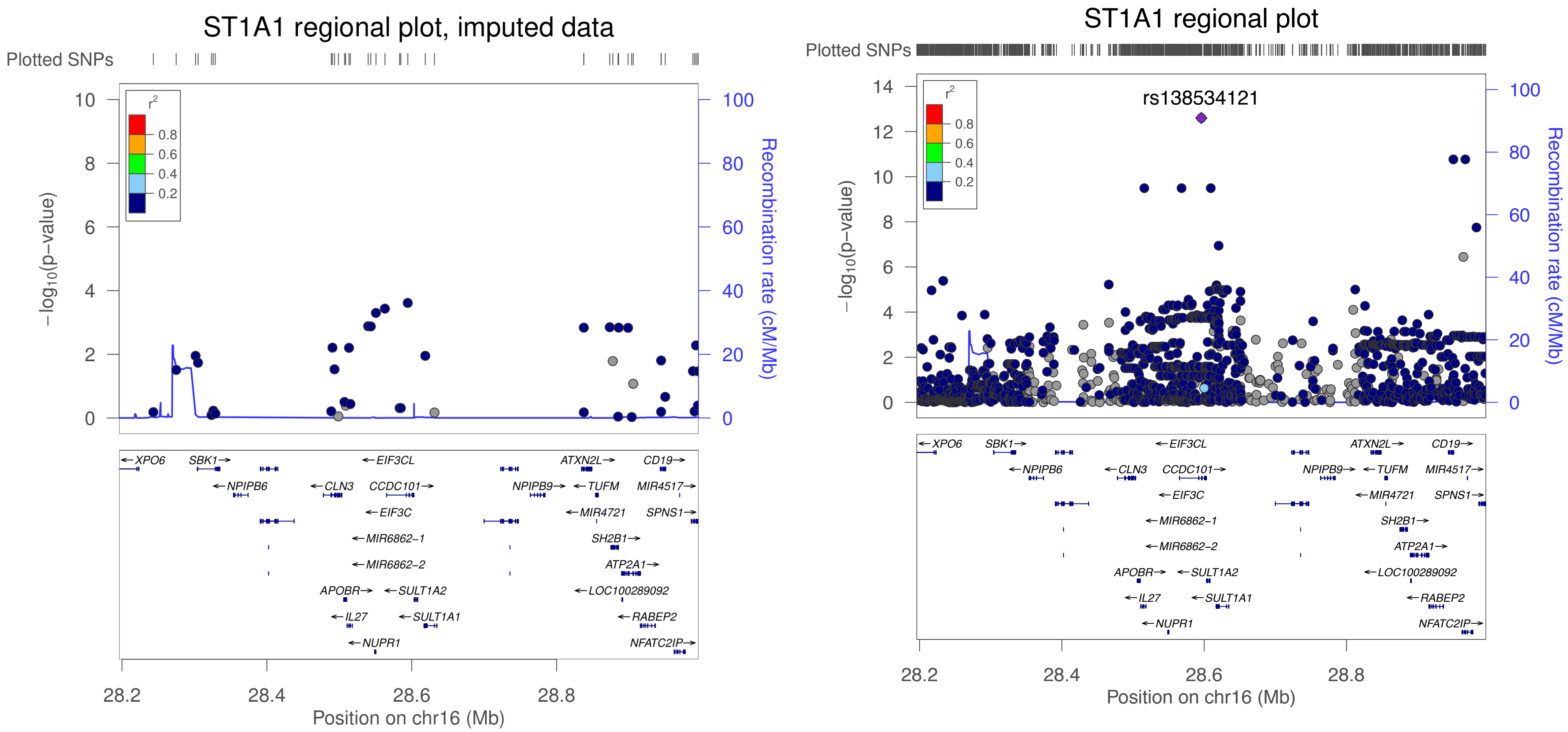

Supplementary Figure S16. Regional association plots for STAMBP. Imputed data to the left and WGS data to the right. No usable LD information could be loaded for the reference SNP. The top variant (1:53206258) could not be plotted. The variant with the second lowest p-value is highlighted instead.

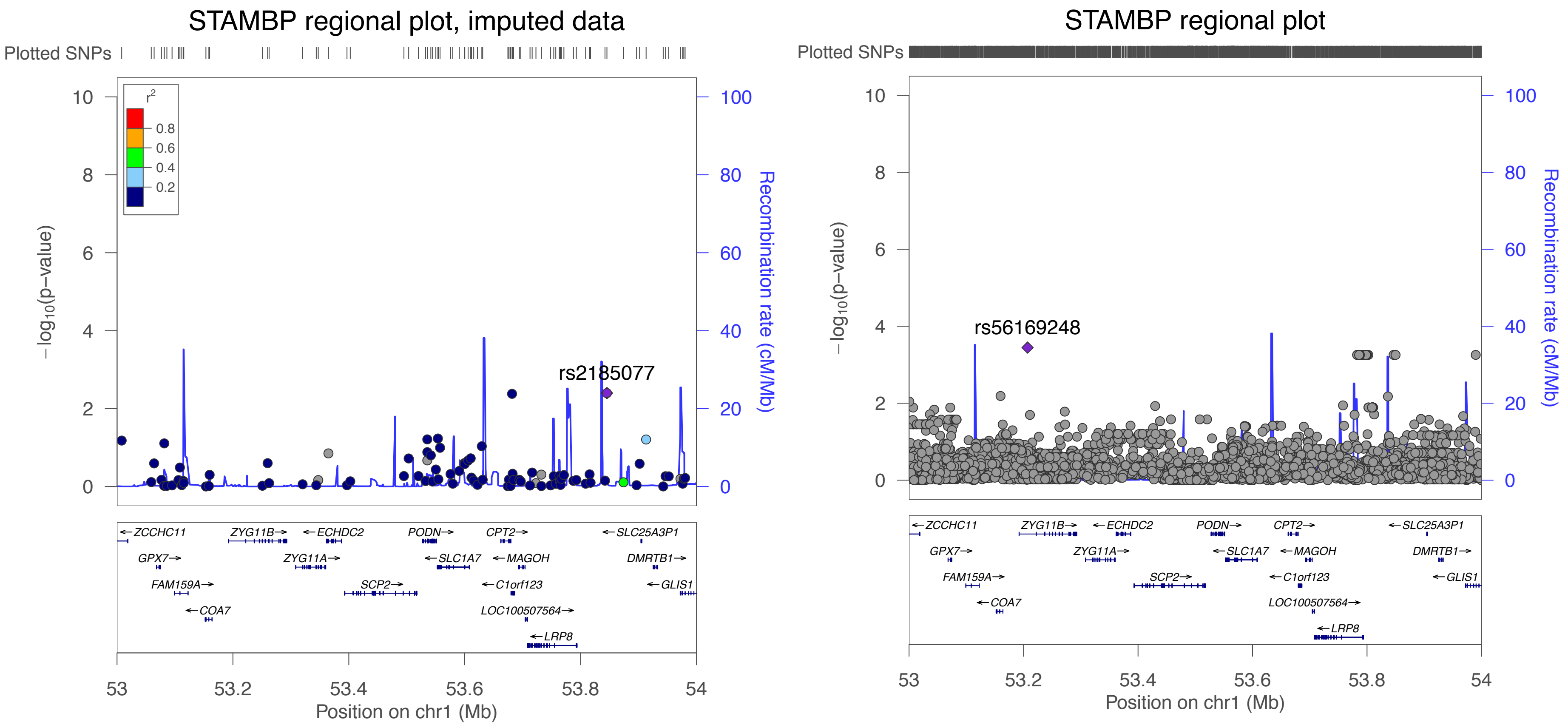

Supplementary Figure S17. Regional association plots for TGFB1. Imputed data to the left, WGS data from INF in the middle and from ONC_CVD, the technical replicate, to the right.

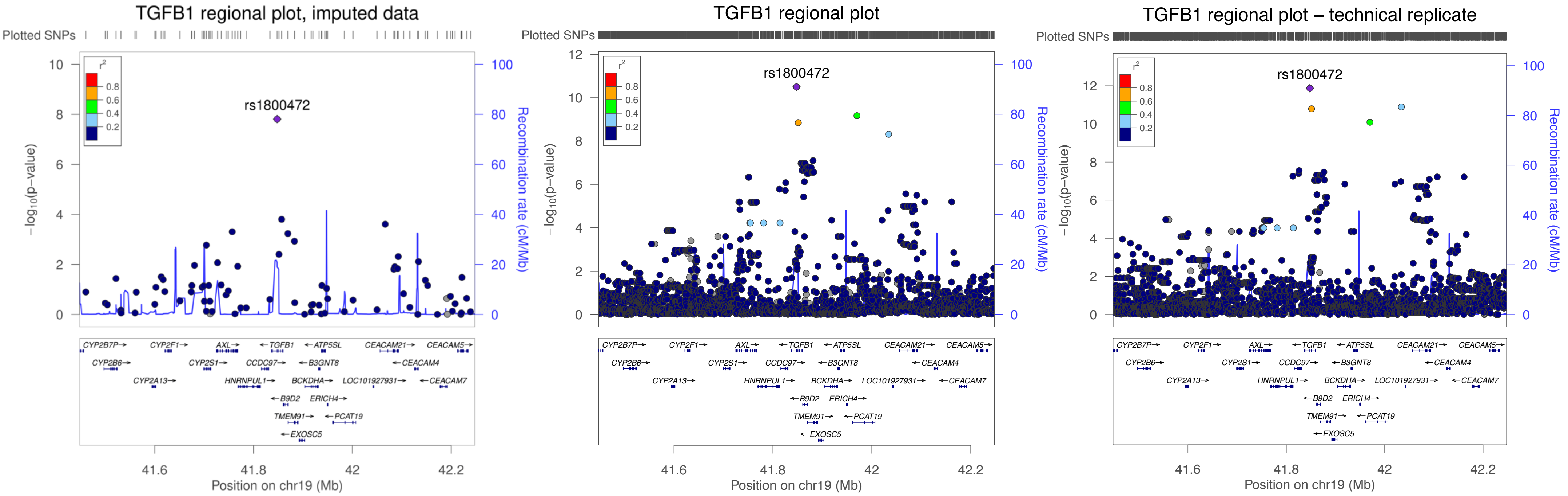

Supplementary Figure S18. Regional association plots for TNFB. Imputed data to the left and WGS data to the right. When analyzing the imputed data, TNFB only had a significant (shown in the plot below) association in the smaller sub-cohort (recruited in 2009) and not in the other sub-cohort (recruited in 2006). This biomarker was therefore note used in the replication step[2] and was not reported as a significant association in the previous study.

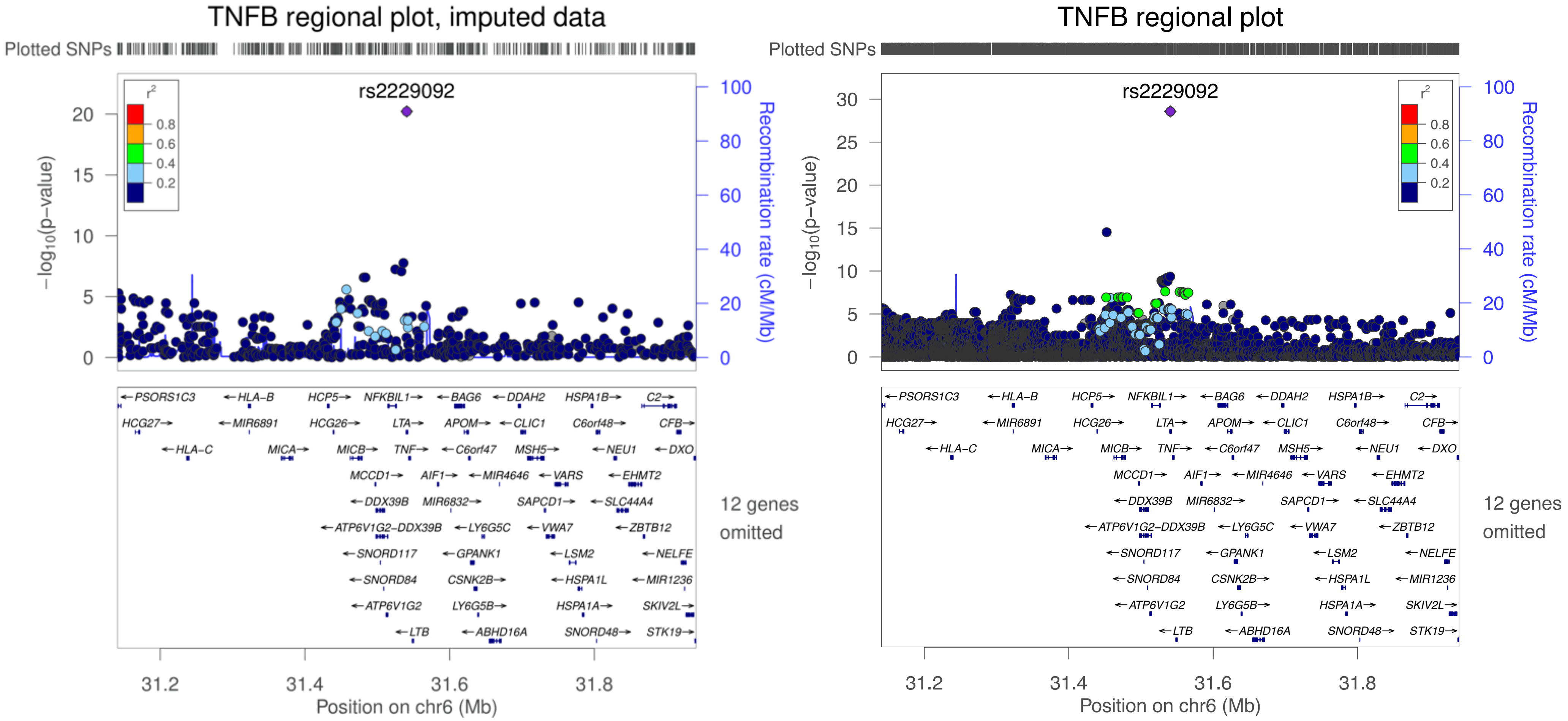

Supplementary Figure S19. Regional association plots for TNFSF14. Imputed data to the left and WGS data to the right.

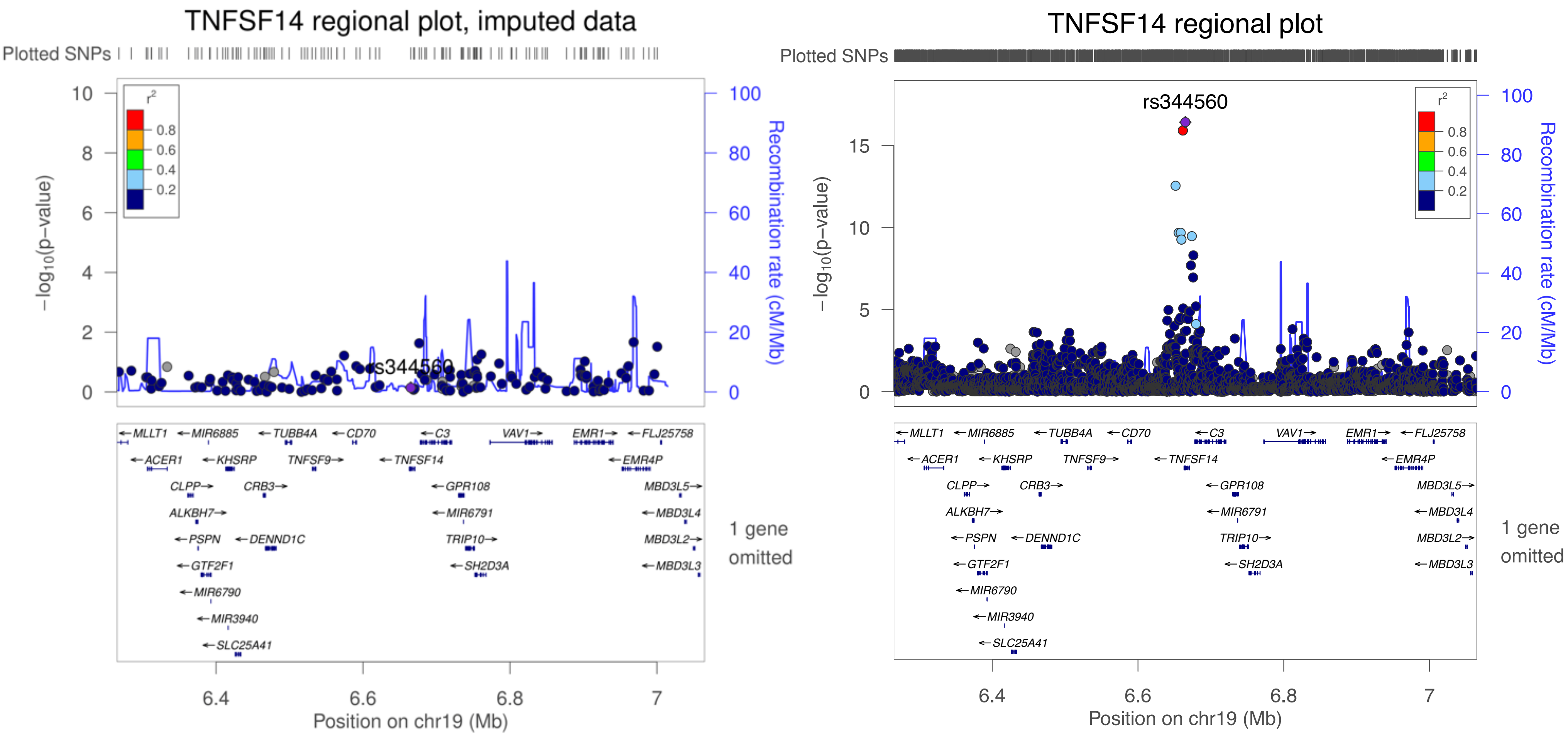

Supplementary Figure S20. Regional association plots for uPA. Imputed data to the left and WGS data to the right.

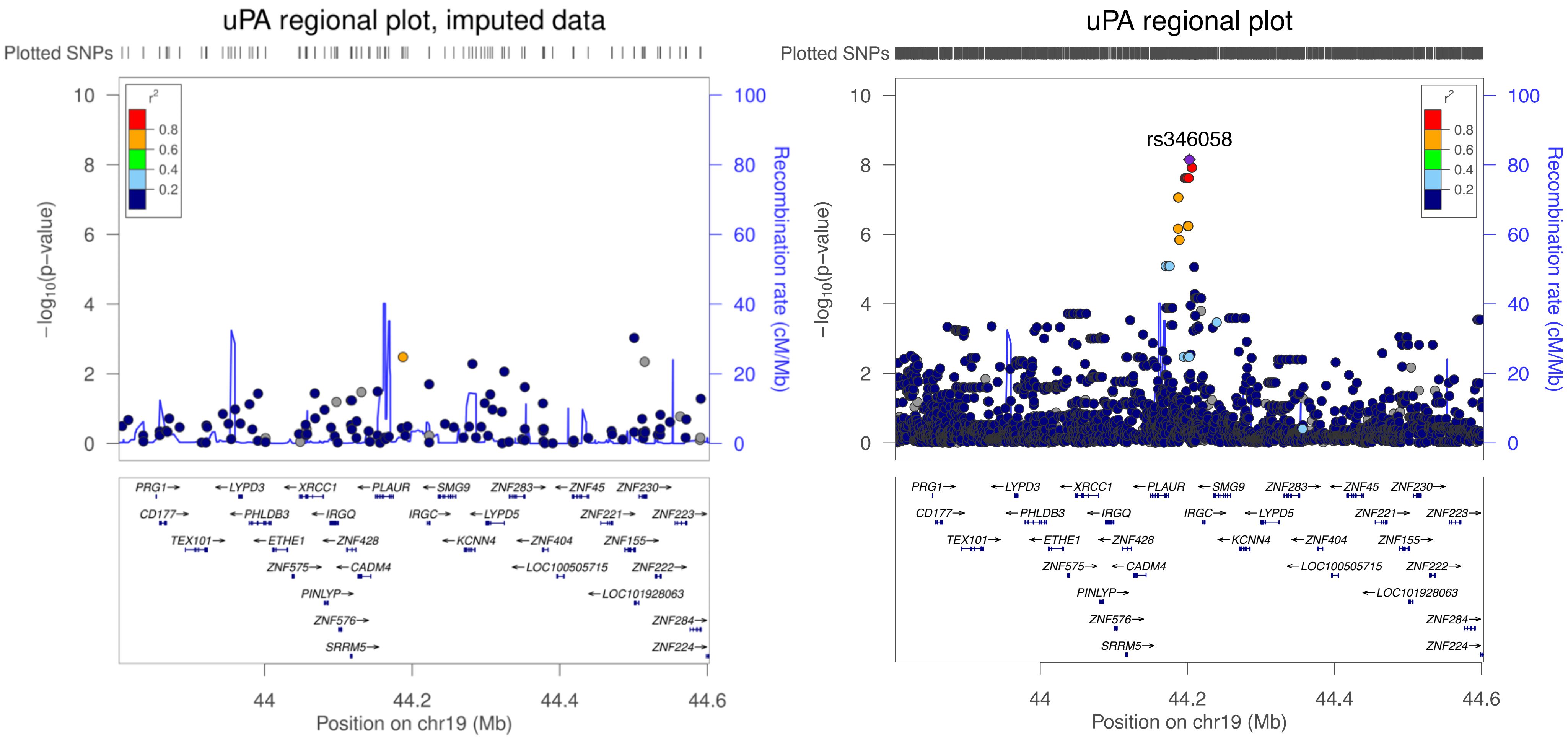

CXCL9/10/11

CXCL10, also known as interferon gamma-induced protein type 10 have previously been associated with blood protein measurements, where the variant rs11548618 has been the top SNV [2,4–6]. In this study, rs11548618 was replicated as the top variant. The beta coefficient has previously been reported as positive [1,2,4]. However, in one of the overlapping measurements, ONC_CVD, the same variant showed a significant association with the nearby chemokines *CXCL9* and *CXCL11* with the opposite direction. This association is not apparent in the INF measurement, where the SNV (rs11548618) does not reach nominal significance (CXCL9: p=0.78, CXCL11: p=0.68). All variants that are significantly associated with *CXCL9* or *CXCL11* in ONC_CVD are associated with *CXCL10* in INF, with a positive beta coefficient in INF and a negative in ONC_CVD. The variants associated with *CXCL9* and *CXCL11* in ONC_CVD are also associated with *CXCL10*, with a more significant association. The genes encoding these are all closely located at chromosome 4. The consequence of the minor allele is a missense mutation resulting in an amino acid change from arginine to cysteine. It is possible that this results in a change in antibody affinity when measuring the blood plasma proteins. The PEA is dependent on proximity antibody probe pairs, which binds to their specific antigens and then hybridize. By adding DNA polymerase, and extension and joining of the oligonucleotides is produces and this forms a PCR template [7]. When carrying the allele that leads to a missense mutation, it might be that the antibodies normally binding to *CXCL9* and *CXCL11* have a higher probability of binding to *CXCL10*. The hybridization at *CXCL9* and *CXCL11* cannot form if not both antibodies bind. This then results in a seemingly lower concentration of *CXCL9* and *CXCL11*, when in reality, the protein concentrations could not be accurately measured. Since ONC_CVD have been measured in the cohort on an earlier timepoint than INF, the panels have been refined and the antibody binding have become more precise. rs115485618 has been significantly associated with *CXCL10* blood plasma measurements before, thus it is more plausible that this is the true association. The previous studies from the same cohort have used genotyped/imputed data [1,2,6]. There were no associations to those biomarkers that passed genome-wide significance but it might just be due to power issues, when comparing results from genotyped/imputed sequence data to whole genome sequence data.

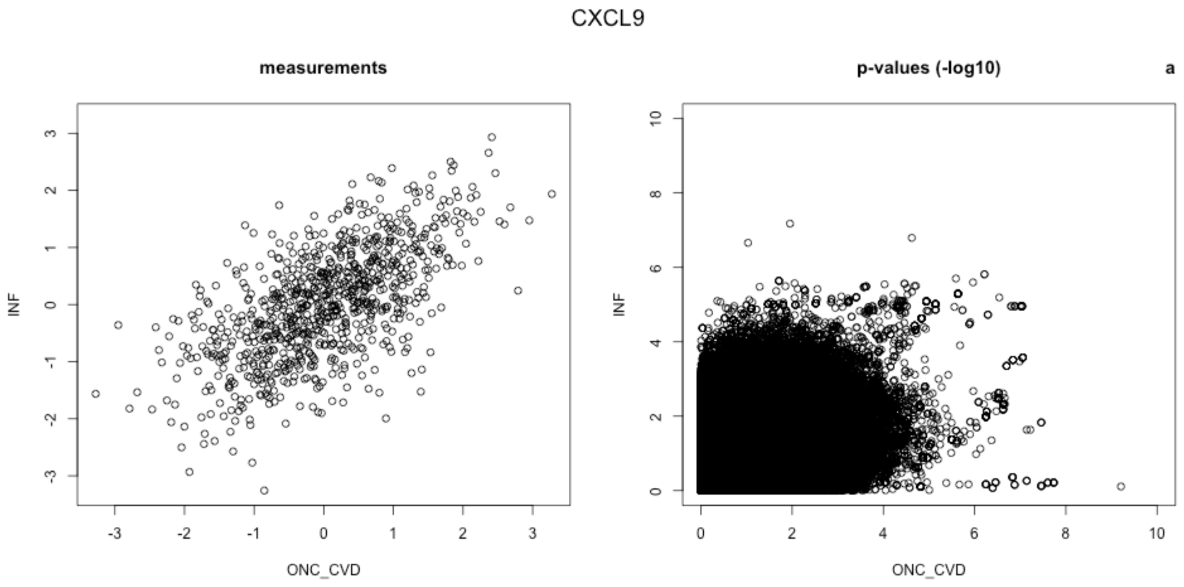

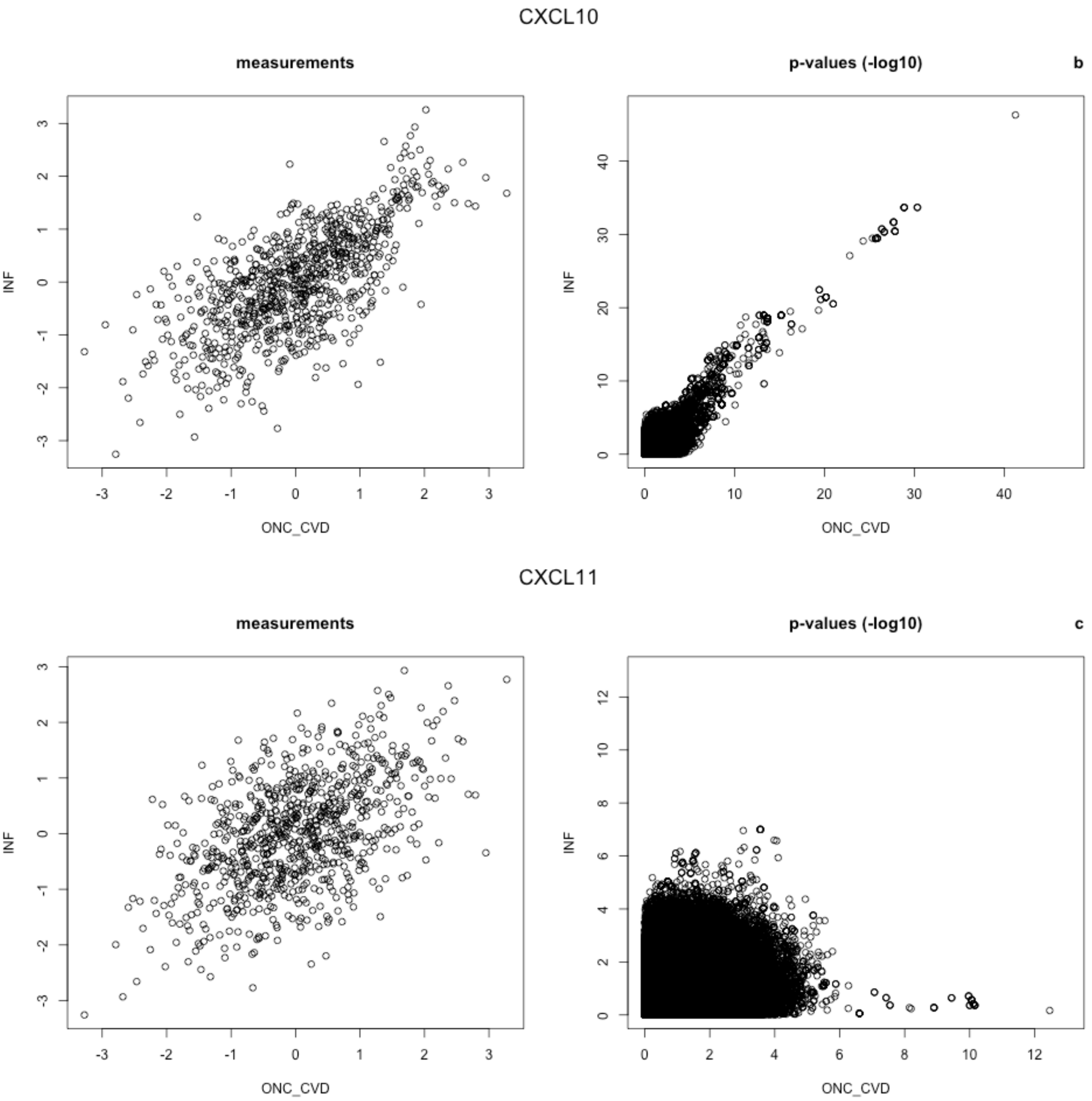

Supplementary Figure S21. Left: correlation of plasma protein level measurements between ONC_CVD (x axis) and INF (y axis). Right: correlation of nominal p-values from the GWA analysis. Results from ONC_CVD on the y axis and INF on the x axis. **A**) Measurements from CXCL9. **B**) Measurements from CXCL10. **C**) Measurements from CXCL11.

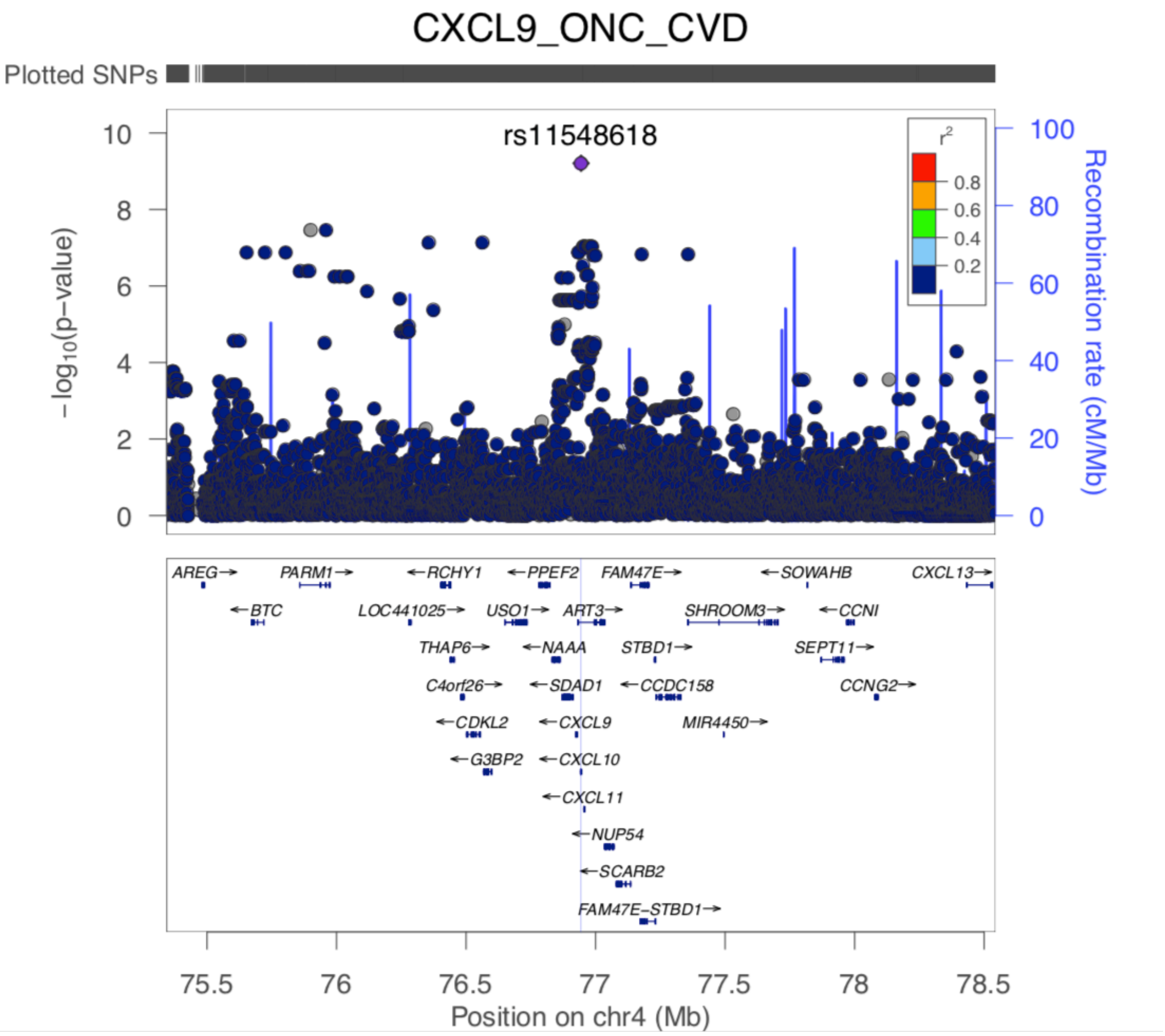

Supplementary Figure S22. Regional association plot of SNV rs11548618 for the association with CXCL9 in ONC_CVD. The highlight is covering the variant ±50bp.

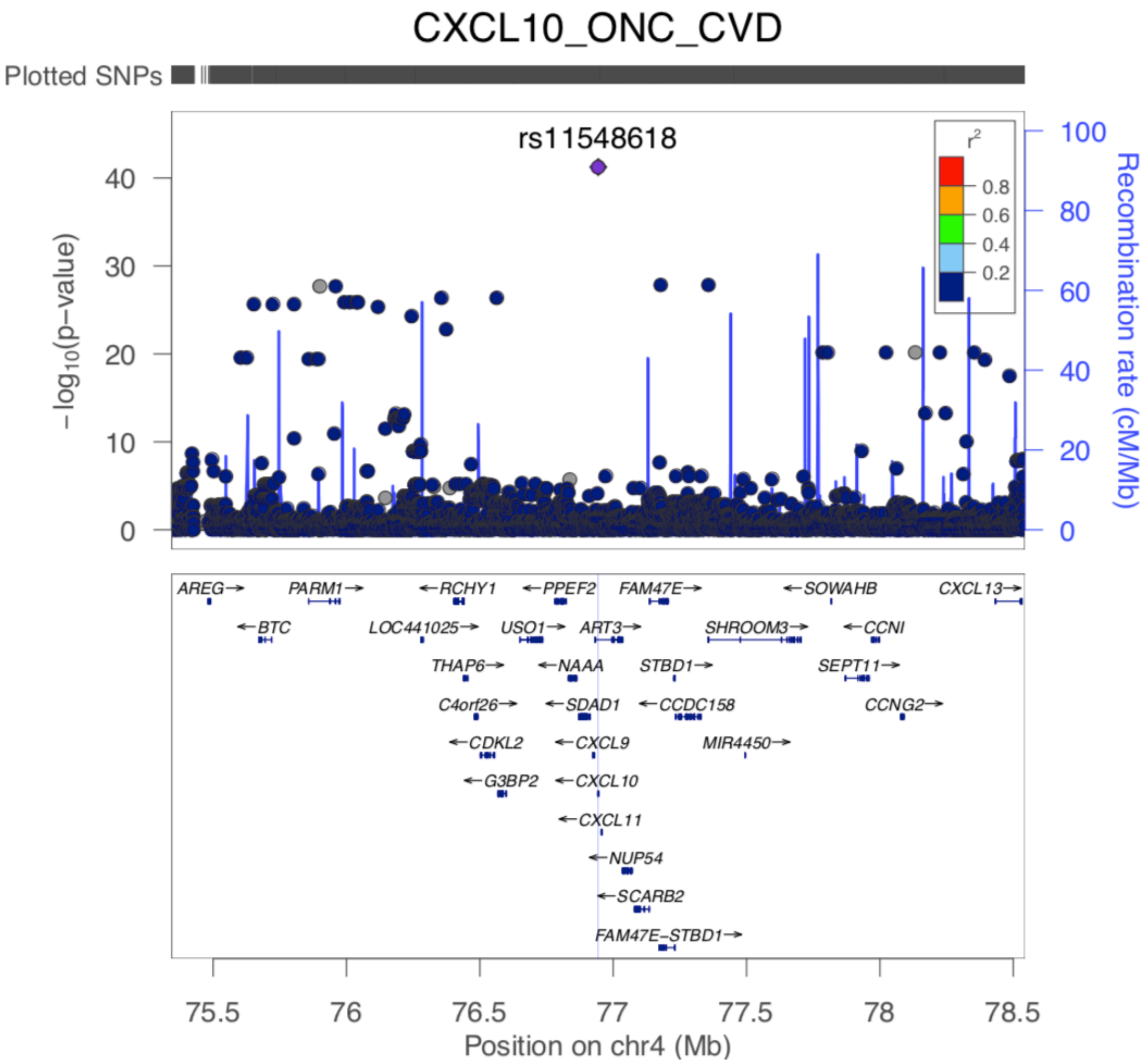

Supplementary Figure S23. Regional association plot of SNV rs11548618 for the association with CXCL10 in ONC_CVD. The highlight is covering the variant ±50bp.

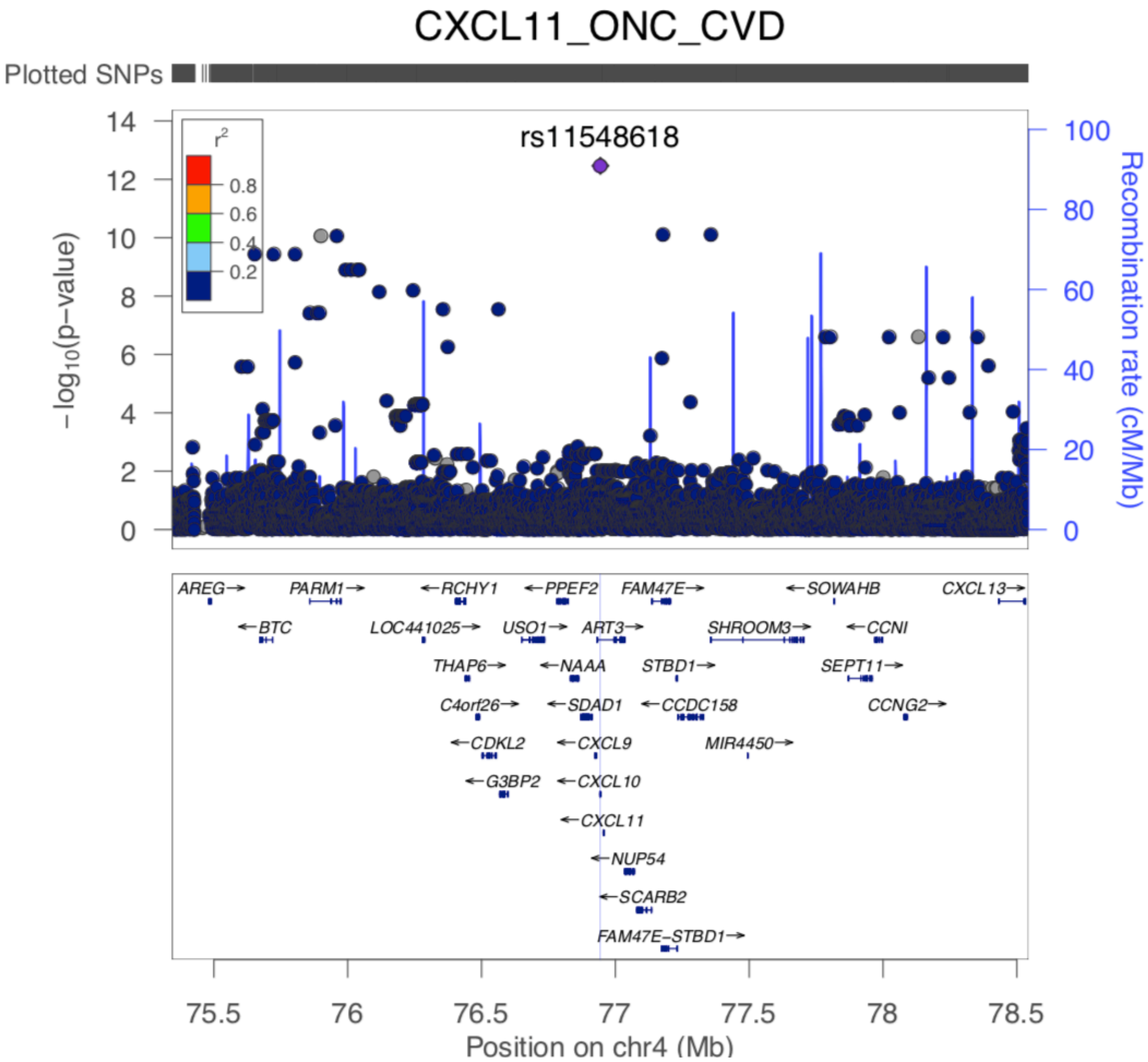

Supplementary Figure S24. Regional association plot of SNV rs11548618 for the association with CXCL11 in ONC_CVD. The highlight is covering the variant ±50bp.

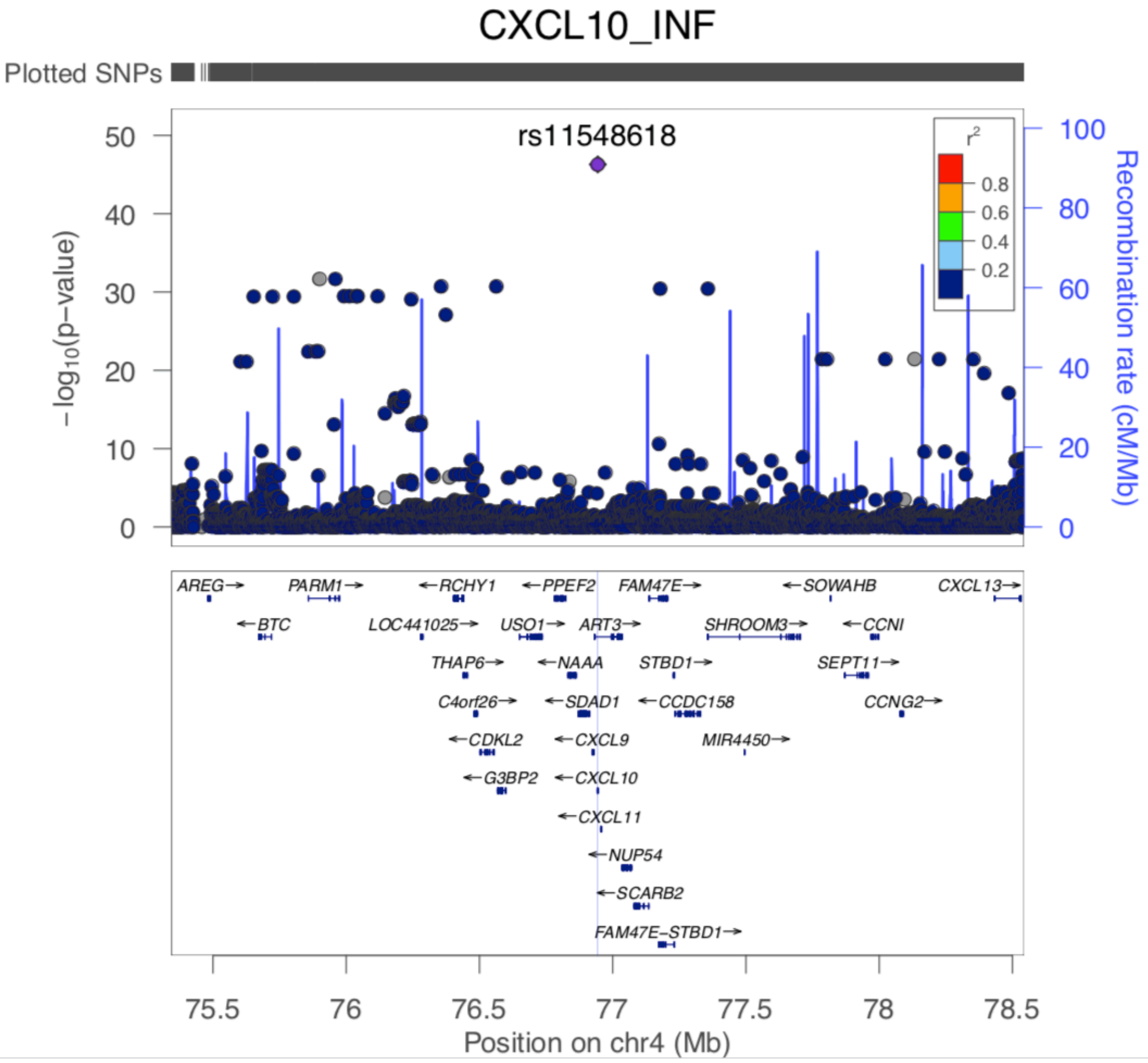

Supplementary Figure S25. Regional association plot of SNV rs11548618 for the association with CXCL10 in INF. The highlight is covering the variant ±50bp.

Supplementary Figures S26-S30. Locus zoom plots for the loci where at least one significant hit was found with WGS data and with genotyped/imputed data but the top variants are in LD < 0.8. All graphs have colored dots based on the LD data information from 1000G nov 2014 EUR dataset. Grey dots depict when no usable LD information could be found for the reference SNP or other SNPs in relation to the reference SNP.

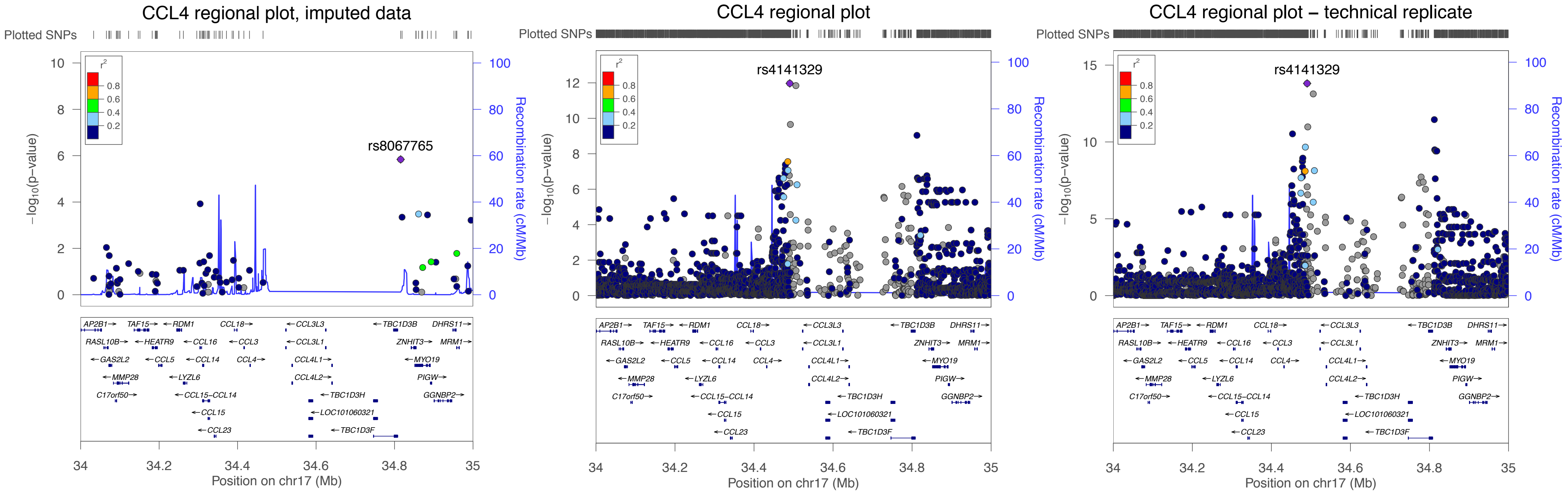

Supplementary Figure S26. Regional association plots for CCL4. Imputed data to the left, WGS data from INF in the middle and from ONC_CVD, the technical replicate, to the right.

Supplementary Figure S27. Regional association plots for CCL19. Imputed data to the left and WGS data to the right.

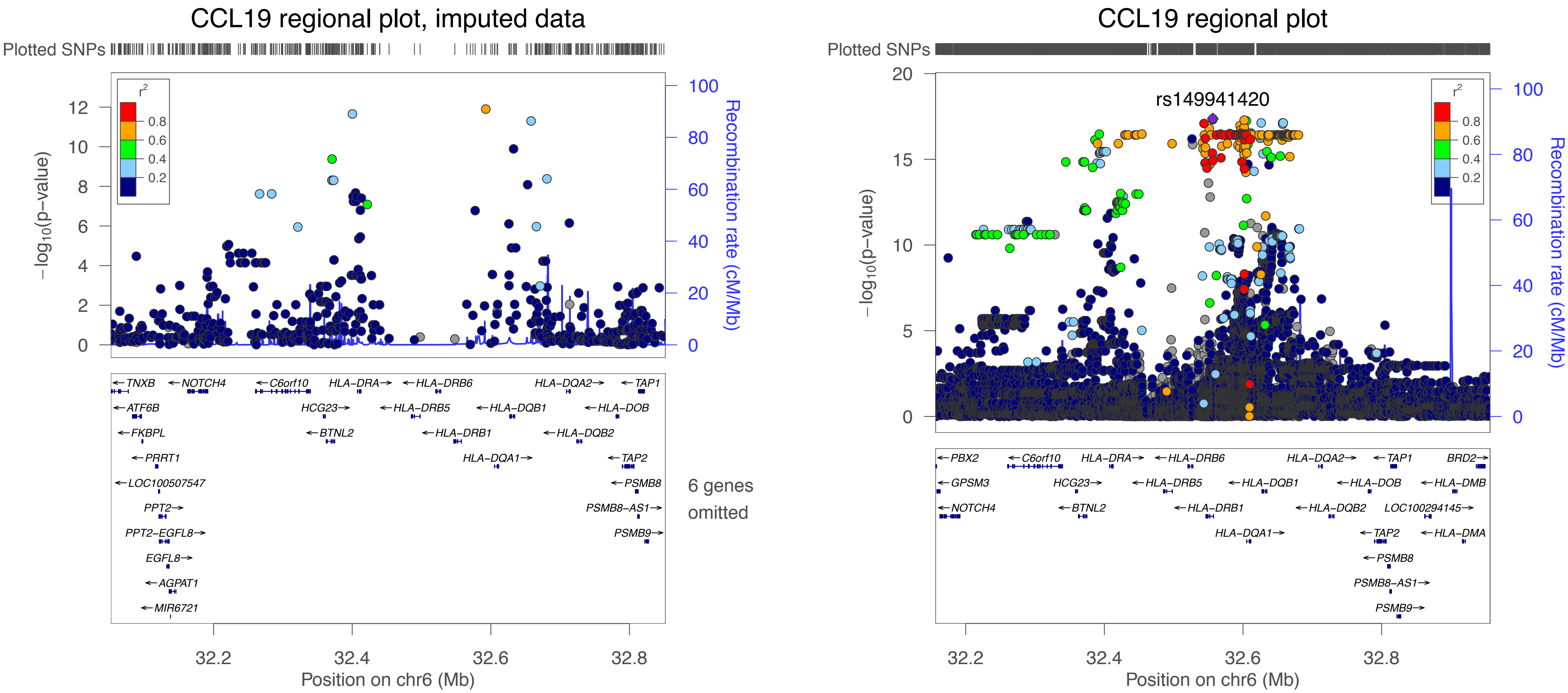

Supplementary Figure S28. Regional association plots for CD6. Imputed data to the left and WGS data to the right.

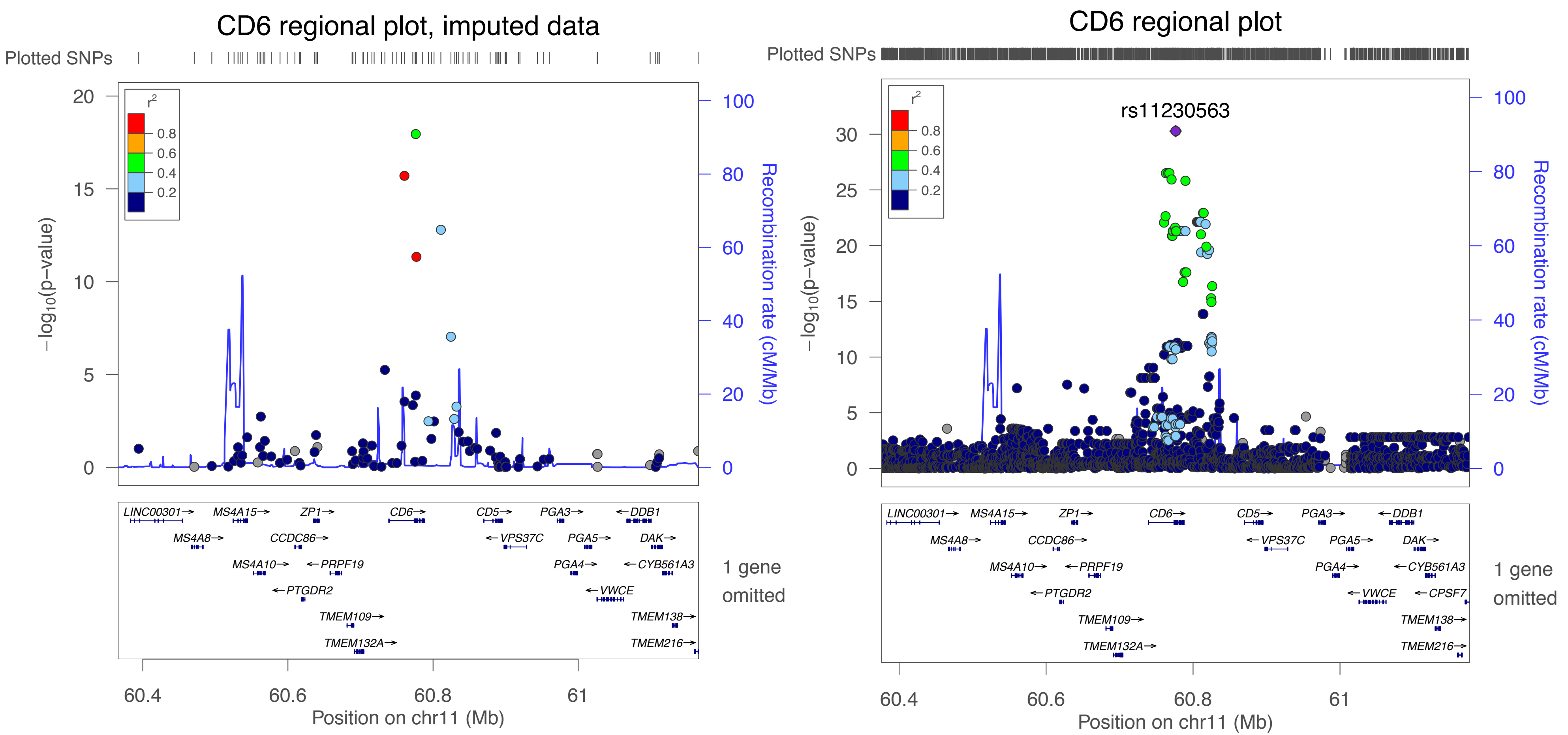

Supplementary Figure S29. Regional association plots for CXCL5. Imputed data around rs10740118 to the upper left, for rs7088799 to the lower left and WGS data from INF on the top right and from ONC_CVD, the technical replicate, on the lower right.

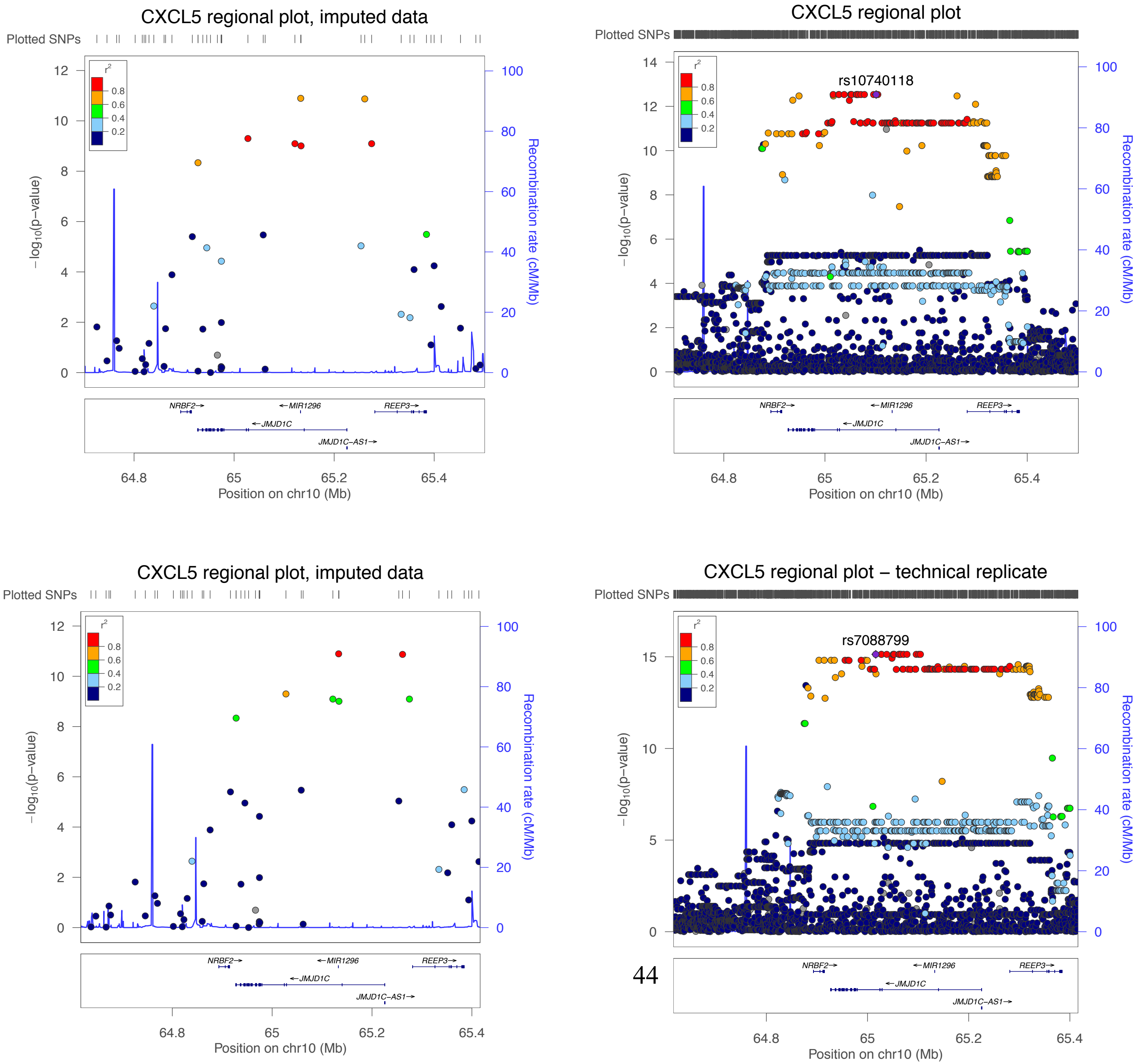

Supplementary Figure S30. Regional association plots for MMP-10. Imputed data to the left and WGS data to the right.

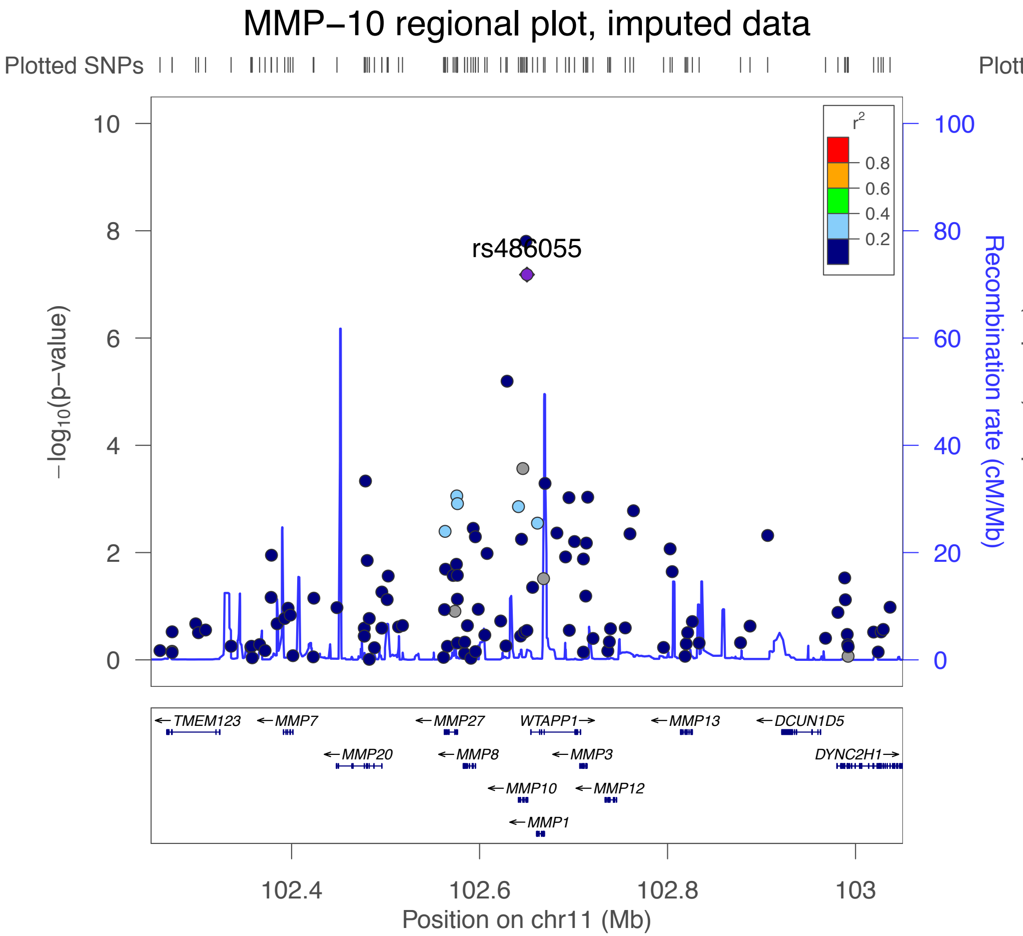

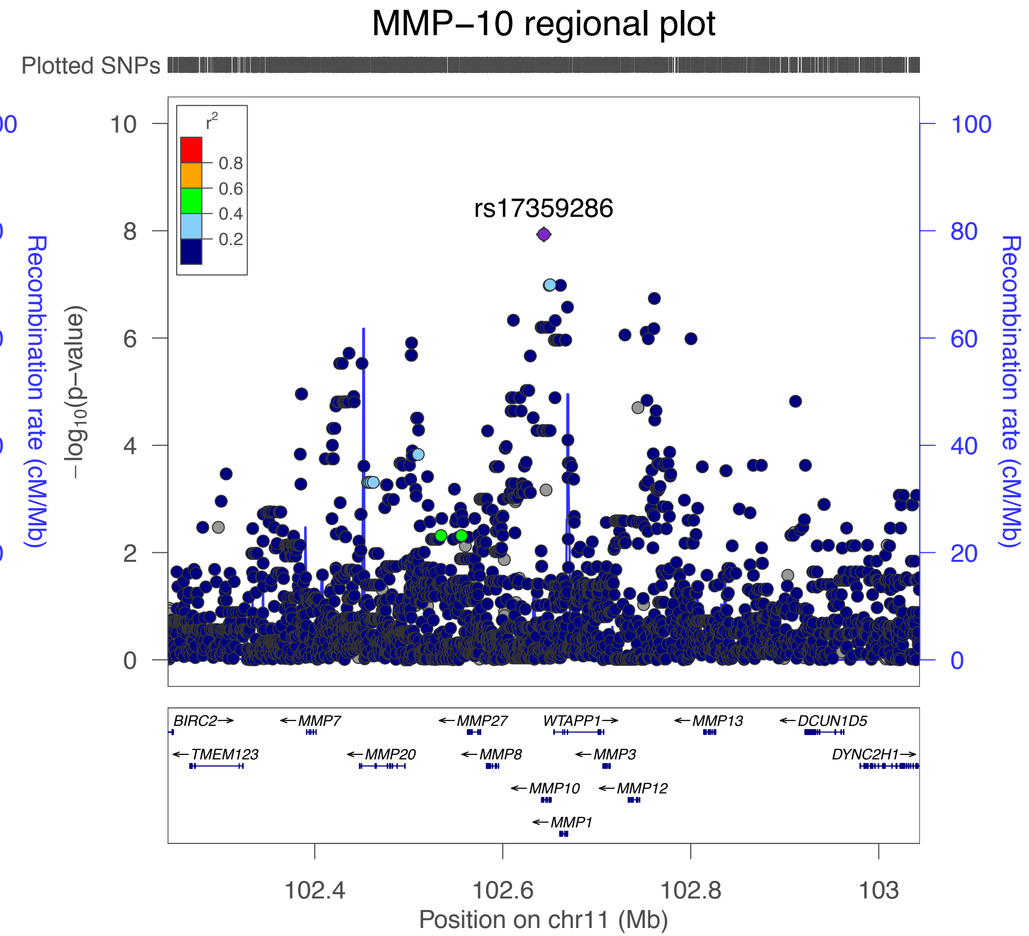

References

1. Ahsan, M. *et al.* The relative contribution of DNA methylation and genetic variants on protein biomarkers for human diseases. *PLoS Genet.* **13**, 1–24 (2017).
2. Enroth, S. *et al.* Systemic and specific effects of antihypertensive and lipid-lowering medication on plasma protein biomarkers for cardiovascular diseases. *Sci. Rep.* **8**, 1–10 (2018).
3. Li, H. & Wren, J. Toward better understanding of artifacts in variant calling from high-coverage samples. *Bioinformatics* **30**, 2843–2851 (2014).
4. Suhre, K. *et al.* Connecting genetic risk to disease end points through the human blood plasma proteome. *Nat. Commun.* **8**, (2017).
5. Sun, B. B. *et al.* Genomic atlas of the human plasma proteome. *Nature* **558**, 73–79 (2018).
6. Enroth, S., Johansson, Å., Enroth, S. B. & Gyllensten, U. Strong effects of genetic and lifestyle factors on biomarker variation and use of personalized cutoffs. *Nat. Commun.* **5**, (2014).
7. Stenvang, J. *et al.* Homogenous 96-Plex PEA Immunoassay Exhibiting High Sensitivity, Specificity, and Excellent Scalability. *PLoS One* **9**, e95192 (2014).
